# Adaptive Trial Designs for Assessing Predictiveness of a Biomarker in Early Phase Drug Development

**DOI:** 10.1002/sim.70719

**Published:** 2026-09-08

**Authors:** Libby Daniells, Julia Geronimi, Hugo Hadjur, Sandrine Guilleminot, Pavel Mozgunov

**Affiliations:** ^1^ MRC Biostatistics Unit University of Cambridge UK; ^2^ Translational Statistics Department Institut de Recherches Internationales Servier Gif‐sur‐Yvette France; ^3^ Department of Statistical Methodology Saryga France

**Keywords:** adaptive design, biomarker subgroups, biomarkers, personalised medicine, small sample size

## Abstract

Biomarker identification plays a crucial role in precision medicine, where treatments are tailored to patients' intrinsic factors. Predictive biomarkers can be used to identify patients who are more likely to benefit from a treatment. However, identifying and validating such biomarkers pose substantial statistical and practical challenges, especially in the small sample setting. Several methods have been proposed for assessing whether a biomarker has a predictive effect, including the Average Kolmogorov‐Smirnov Approach (AKSA), which has been shown to have improved operating characteristics for inference compared to alternative approaches. Yet power remains limited when sample sizes are small and unbalanced across treatment arms. Power can be improved through adaptive enrichment of the sample size, especially when treatment arms are small and unbalanced. We propose an adaptive design to improve power for detecting a predictive effect when sample sizes are small. Existing adaptive biomarker designs largely focus on enrichment of the trial, for example, adjusting enrolment criteria based on an assessment of the treatment‐biomarker interaction at interim, or refining the biomarker threshold used to segregate the population into biomarker positive or negative (i.e., those patients more or less likely to respond to the treatment). Few designs explicitly address the task of formally declaring a predictive effect of a biomarker using an interim analysis. The interim analysis framework enables a more reliable assessment of a biomarker's predictive effect by introducing a pre‐planned evaluation at the originally intended sample size of a study which may otherwise be underpowered due to the limited number of patients. Based on the interim results, the trial may (i) already have sufficient evidence to declare a biomarker as predictive, (ii) be terminated early due to a clear lack of evidence for a predictive effect, or (iii) enter an “expansion phase”, where additional cohort of patients are recruited to improve the power for final analysis. The proposed framework addresses key methodological challenges, including interim decision rules, determining the magnitude and allocation of post‐interim sample size increases, controlling the Type I error rate, and achieving meaningful gains in statistical power at the final analysis. The impact of an unknown biomarker distribution is also considered. These challenges are explored through simulation studies, where properties of the interim and final analyses are presented under various design configurations (where the decision bounds and size of the expansion are altered) and scenarios, in which the true predictive and prognostic effects of the biomarker are varied. Evaluated metrics include statistical power, Type I error control, and interim decision characteristics. The results inform the design of trials incorporating interim analyses to assess the predictive effect of a biomarker, enabling statistically efficient and well‐calibrated decision‐making.

## 1 Introduction

Progress in understanding underlying disease mechanisms has propelled the concept of precision medicine to the forefront of clinical trial design. Clinical trials have traditionally assumed that all patients respond similarly to treatment [1], however, it is now well established that treatment response varies substantially across patients due to differing biological and clinical characteristics. Biomarkers—that is, measurable biological indicators—play a crucial role in identifying patient subgroups more likely to benefit from a treatment. Where validated biomarkers are identified prior to the trial, they can be incorporated into the design and inclusion criteria to enrol only those expected to benefit. This is known as a biomarker‐enrichment design [2], which can improve trial efficiency and power. However, relevant biomarkers are often unknown prior to the trial and only identified through post hoc analysis, where classification and validation is particularly challenging due to small data sets common in early‐phase trials [3].

Biomarkers can be broadly classified as either prognostic or predictive. Prognostic biomarkers are associated with the disease outcome irrespective of treatment, whereas, predictive biomarkers can be used to identify a population who are more or less likely to benefit from treatment [4]. A threshold can be derived from a predictive biomarker to segregate the population into those more (biomarker positive, BMK+) or less (biomarker negative, BMK‐) likely to benefit from the treatment. Identifying predictive biomarkers requires a randomised control trial (RCT), including patients with a range or biomarker values [5]. Without a control arm, the predictive and prognostic effects cannot be distinguished [6].

Serra et al. [6] compare several methods for establishing whether a biomarker has a predictive effect, including the novel Average Kolmogorov‐Smirnov approach (AKSA), which models the relationship between the outcome and biomarker by computing the average difference in the estimated treatment effect (the difference between the experimental and control arm) across biomarker values. In small sample settings, across scenarios varying treatment effects, biomarker effects and biomarker distributions, AKSA demonstrated an improved ability to detect truly predictive biomarkers compared to existing methods, while controlling the Type I error rate and achieving high power for strong predictive signals, regardless of the biomarker distribution. However, more moderate predictive effects remained difficult to detect under the small and unbalanced sample sizes considered (40 patients in the experimental arm and 20 in the control arm). While more power could be attained through larger or more balanced samples, such expansions are not routinely justified in early phase drug development without supporting evidence. Adaptive trial designs offer a principled framework for efficiently augmenting the study based on accumulating evidence.

Adaptive clinical trials allow pre‐planned modifications based on accumulating data [7]. Adaptive designs are well‐established for primary endpoint analysis. Their acceptability is reinforced by the ICH E20 guidance [8], which provides a framework for implementing adaptive designs for confirmatory trials, detailing pre‐planned modifications that preserve trial integrity and statistical validity. Several adaptive designs incorporate biomarkers. Adaptive enrichment designs use interim data to modify the recruited population based on evidence of a treatment‐biomarker interaction [9, 10]. Thresholds for defining BMK+ and biomarker BMK− populations can be similarly refined at interim [11], and response‐adaptive randomisation can adjust the treatment allocation based on biomarker status [12]. Such designs typically guide enrolment or final analysis in later‐phase studies with larger sample sizes [13, 14, 15], however, there has been a recent shift towards biomarker‐driven designs in earlier phases of drug development (Phase I/II studies) [16, 17]. Evaluating and validating biomarkers in the early phases of drug development, allows them to guide decision‐making and refine target populations in later‐phase studies [18], with the potential to increase trial success rates.

In practice, hypotheses regarding a biomarker's predictive effect are often biologically motivated or generated from pre‐clinical or real‐world data. They and are typically first tested in a proof‐of‐concept (PoC) study, whose evidence frequently informs whether a biomarker‐driven strategy is carried forward, potentially directly to a Phase III trial. An early, reliable assessment of the predictive effect is therefore crucial, as false predictive effect conclusions can have substantial downstream consequences for trial design, patient selection and resource management in later phase studies. However, Phase I//II trials are typically driven by the primary efficacy endpoint rather than biomarker predictiveness, limiting sensitivity to detect predictive effects precisely. Further statistical challenges also arise from small, unbalanced sample sizes, including sparse biomarker data that may not reflect the whole population, difficulty distinguishing prognostic from predictive effects and issues with biomarker cut‐off identification.

While adaptive enrichment and alternative adaptive designs focus on modifying enrolment and biomarker identification, few formally address declaring a predictive effect at interim, particularly in early‐phase trials. To our knowledge, approaches for assessing biomarker predictiveness have not been formalised for Phase I/II trials with small and potentially unbalanced sample sizes. The proposed methodology bridges this gap, introducing an interim analysis framework for assessing the predictive effect of a biomarker in the early‐phase, small sample setting. The framework can be viewed as a cohort expansion with a formal interim analysis. The interim analysis allows for an early assessment of the predictive effect based on the accumulating evidence and informs whether an expansion phase is required. In settings with small sample sizes, increasing the number of patients represents a natural strategy to enhance statistical power. However, due to financial and time constraints, trial expansion is most appropriately considered when supported by accumulating evidence. Thus, the developed interim framework allows for early termination where evidence against a predictive effect is sufficient, early declaration where evidence is already sufficient in its favour, and expansion only where further information is needed to make a reliable conclusion. This extends prior approaches, which assessed predictiveness only at trial completion, enabling earlier identification of a predictive effect while addressing the lack of power for the main analysis. This supports clinical decision‐making and informs subsequent trials.

Expansion cohorts are now frequently used in Phase I oncology trials, often to explore, safety or dosage of a treatment, alongside preliminary efficacy findings. However, as stated by Ezzalfani et al. [19], despite the widespread use of expansion cohorts, the sample sizes of the expansion cohorts are rarely statistically justified and are selected based on practical considerations rather than explicitly to optimise operating characteristics of the trial. In fact, in a recent review by Herrero Colomina et al. [20] into expansion cohorts in Phase I oncology trials, across 479 studies they found that 44% of trials failed to even state an objective of the expansion, with just 24% including a justified statistical plan. This builds on earlier work by Norris et al. [21] and Manji et al. [22], who found a similar lack of detail and justification in early‐phase expansion cohort studies. This review demonstrates that current practice for expansion cohorts is largely ad hoc, which contrasts pre‐planned two‐stage designs such as Simon's two‐stage design [23], where interim decision rules are formally specified prior to the trial. However, designs such as Simon's two‐stage are typically driven by the trial's primary objective, most commonly overall efficacy, rather than for assessing the predictive effect of a biomarker, and therefore do not necessarily provide a statistically justified approach to the biomarker question. The proposed framework aims to bring the pre‐planned, statistically justified approach to expansion cohorts for assessing the predictive effect of a biomarker, moving away from ad hoc expansions whose sample sizes and decision rules are rarely optimised in terms of the trials operating characteristics.

Conducting an interim analysis for declaring a predictive effect introduces key methodological challenges: assessing predictiveness with limited data at an interim time point, particularly in the early‐phase setting; assessing predictiveness with unbalanced sample sizes across treatment arms (early phase studies typically focus on gaining information on the experimental arm, for example, for safety, tolerability and a preliminary efficacy analysis, rather than comparison to the control); controlling Type I error rate inflation (probability of declaring a non‐predictive biomarker as predictive) from multiple looks at the data; determining optimal interim and expansion sample sizes; selecting an approach to declare predictiveness at each analysis point; and defining interim decision rules. Other challenges include an unknown biomarker distribution and the presence of a prognostic effect. The work presented in this manuscript explicitly addresses each of these concerns, providing practical guidance for future trial applications. To illustrate the proposed interim analysis framework, the early‐phase trial for Non‐Small Cell Lung Cancer (NSCLC) conducted by Servier is used as a motivating example.

This manuscript is structured as follows: Section 2 outlines the motivating NSCLC Servier trial, including settings, objectives and notation. Section 3 describes the AKSA method used to assess whether a biomarker has a predictive effect. Section 3 also outlines the interim analysis framework for assessing predictiveness, including the decision bounds and sample size implications. In Section 4, a thorough simulation study is conducted based on the motivating example. The simulations explore different considerations/concerns surrounding interim analyses and expansion cohorts. Finally in Section 4.7 a sensitivity analysis is conducted, exploring the effect of the biomarker distribution, and initial sample sizes and allocation ratios.

## 2 Motivating Example

### 2.1 Trial Setting

As in the work by Serra et al. [6], this work is motivated by the Phase I/II randomised Non‐Small Cell Lung Cancer (NSCLC) trial conducted by Servier. In this trial, an enzyme promoting immune suppression is considered as a biomarker. The primary objective of the study is to assess the overall treatment effect (i.e., difference in response between the experimental and control arm) regardless of biomarker status, however, it is believed that the biomarker has a potential predictive effect of a patients overall response to treatment. Thus, a secondary objective of the study is to assess the predictive effect of the biomarker.

The planned total sample size of the initial PoC study is 60 patients randomised between an experimental and control arm. A 2:1 allocation ratio will be implemented in favour of the experimental arm, leading to small and unbalanced sample sizes. Such an allocation ratio was selected based on the primary analysis strategy, where Bayesian dynamic borrowing will be applied to leverage information from historic control data. This thereby allowed the resources to be focused on the experimental arm within the study. Therefore, although this design choice was beneficial for the primary analysis of the PoC study given the availability of the historic data, it is to the detriment of assessing the predictive effect of the biomarker. The assessment of the predictive effect was not a primary objective when designing the trial but has now emerged as a major consideration.

In this trial setting, Serra et al. [16] explored several approaches for assessing the predictive effect of a biomarker, including their novel Average Kolmogorov‐Smirnov approach (AKSA). When applied at the end of the initial PoC study (i.e., using the full PoC sample size of 60 patients with a 2:1 allocation), the results showed that 80% power to detect a predictive effect could only be achieved in the presence of a very strong predictive signal. For more moderate predictive signals, less than 50% power was observed regardless of the biomarker distribution and prognostic effect. These findings indicate that the initial PoC sample size provides limited ability to reliably assess the predictive effect of the biomarker.

This motivates the consideration of a PoC expansion cohort, to gain further data if and only if, there is sufficient evidence to suggest a predictive signal may be present but is not yet conclusive. Within the proposed interim analysis framework, the interim analysis corresponds to the analysis conducted at the end of the initial PoC phase, while the final analysis is performed after the additional patients from the expansion cohort.

### 2.2 Notation

Binary outcomes are assumed for the trial and as such the overall response rate (ORR) in both the experimental and control arm are modelled through a binomial distribution: $Y_{k}∼\text{Binomial}(n_{k},p_{k})$, where k=0 for the control arm and k=1 for the experimental arm. The sample size in the experimental and control arms are denoted $n_{1}$ and $n_{0}$ respectively, with total sample size of the study being $N=n_{0}+n_{1}$. The total sample sizes per arm, $n_{0}$ and $n_{1}$, can be split into pre and post interim/expansion sample sizes. For example, $n_{0}=n_{0,1}+n_{0,2}$ and $n_{1}=n_{1,1}+n_{1,2}$. Given the setting outlined in Section 2.1, the original trial without expansion consisted of 40 patients on the experimental arm and 20 on the control arm, thus, $n_{0,1}=20$ and $n_{1,1}=40$ are considered as our interim sample sizes. We explore the impact of adding the expansion cohort $n_{0,2}$ and $n_{1,2}$, should the interim data (from the $n_{0,1}$ and $n_{1,1}$ patients) suggest it is required for declaring a predictive effect. Denote this interim data as $\Omega_{0}$. The final data‐set includes all patients throughout the trial (both before and after interim) and is denoted as $\Omega_{1}$. The unknown response rates are $p_{1}$ and $p_{0}$ for the experimental and control arm respectively.

The biomarker, X, is assumed to arise from a continuous distribution, $\phi_{X}(x)$. To be specific, the biomarker is assumed to be Gamma distributed:

$$X∼\Gamma (r,s)\;\text{with}\;\phi_{X}(x)=\frac{r^{s}}{\Gamma (s)}x^{s-1}\exp(-rx)$$

where r is the rate, s is the shape and Γ(·) is the gamma function. The choice of a Gamma distribution is motivated by the biological evidence that biomarkers from gene expression data arise from a skewed rather than a symmetrical distribution, such as the normal distribution [24, 25]. Therefore a skewed distribution, such as the Gamma distribution, better reflects the underlying biological process, in particular in the outlier biomarker values and the tails of the distributions. To reflect this, the shape parameter, s, of the Gamma distribution is selected to be a function of the rate parameter, r, such that $s=650.54r^{2}$, with r=0.083 chosen to create a less‐skewed Gamma distribution with the most weight concentrated around the central biomarker values.

The probability of response, $p_{k}$, for each arm is modelled through a logistic relationship between the biomarker value, X, and treatment indicator $T_{k}$, where $T_{1}=1$ indicates the experimental treatment and $T_{0}=0$ indicates the control arm:

(1) $$p_{k}=\frac{\exp(b_{0}+b_{1}T_{k}+b_{2}X\times T_{k}+b_{3}X)}{1+\exp(b_{0}+b_{1}T_{k}+b_{2}X\times T_{k}+b_{3}X)},$$

where the set of coefficients are denoted $b=\{b_{0},b_{1},b_{2},b_{3}\}$. The predictive effect is captured through the $b_{2}$ coefficient, that is, the interaction between the biomarker and the treatment, while $b_{3}$ reflects the prognostic effect of the biomarker in the control arm. The biomarker effect in the experimental arm is influenced by the predictive effect, $b_{2}$. The treatment effect is encapsulated by both $b_{1}$ and the predictive effect, $b_{2}$.

## 3 Interim Analysis Framework

### 3.1 Background Methodology

Serra et al. [16] outlined several approaches for assessing the predictive effect of a biomarker. This included their novel AKSA approach, which was inspired by the Kolmogorov‐Smirnov distance, taking pairs of biomarker values to compare their treatment response curves, while accounting for uncertainty in order to establish the probability that the treatment effect is positive across the biomarker range. This probability is used to determine whether or not a biomarker has a predictive effect. In detail, AKSA is implemented as follows:

1. Model the biomarker response as a regression model Y∼g(T,X,T×X) and estimate the difference in response between the experimental and control arms as
$${\hat{D}}_{X}(x)=g((T=1),x,(T=1)\times x)-g((T=0),x,(T=0)\times x),$$
  and its standard error $ŝ(x)=\sqrt{se{\left(g((T=1),x,(T=1)\times x)\right)}^{2}+se{\left(g((T=0),x,(T=0)\times x)\right)}^{2}}.$
2. Sample B pairs of biomarker values $\left(x_{1},x_{2}\right)$, with $x_{1}<x_{2}$. For each pair compute $\mu_{i}={\hat{D}}_{X}(x_{2})-{\hat{D}}_{X}(x_{1})$ and $\sigma_{i}=\sqrt{ŝ{\left(x_{1}\right)}^{2}+ŝ{\left(x_{2}\right)}^{2}}$, with i=1,…,B.
3. For each of the B biomarker pairs, sample a value from $Z∼N(\mu_{i},\sigma_{i})$. Denote this value $d_{i}$.
4. Compute the probability that the difference in biomarker‐response is greater than 0: $P(D_{X}>0)=\sum_{i=1}^{B}{𝕀}_{d_{i}>0}/B$, where ${𝕀}_{d_{i}>0}=1$ if $d_{i}>0$ and 0 otherwise.
5. The biomarker is declared predictive if $P(D_{X}>0)>\eta$.

The threshold η for declaring the biomarker as predictive, is pre‐defined in order to control the Type I error rate to a nominal level under specified data scenarios.

### 3.2 AKSA With Interim Analysis

The focus of this work is to specify an adaptive framework for assessing the predictive effect of a biomarker on the treatment outcome. This is achieved through a formal interim analysis, from which, a decision is made regarding whether an expansion cohort is required in order to have sufficient power for declaring that a predictive effect is present. At interim, the AKSA approach—as outlined in Section 3.1—is implemented to make such a decision. As such, the posterior AKSA probabilities at interim ($P(D_{X}>0|\Omega_{0})$) and final analysis ($P(D_{X}>0|\Omega_{1})$) are utilised as follows:

- If $P(D_{X}>0|\Omega_{0})<\eta_{0}$ at interim, the biomarker is said to not show a sufficient predictive effect based on the interim data. Given the clear lack of a predictive signal, no expansion is required and no further patients are recruited. The biomarker is declared as non‐predictive.
- If $P(D_{X}>0|\Omega_{0})>\eta_{1}$ at interim, the biomarker is declared to have a predictive effect based on the interim data. Given that the interim sample sizes ($n_{0,1},\;n_{1,1}$) are sufficient for demonstrating a predictive effect, no further patients are required, thus no expansion occurs and the trial terminates.
- If $\eta_{0}\leq P(D_{X}>0|\Omega_{0})\leq \eta_{1}$ at interim, there is insufficient evidence to declare the biomarker as predictive or non‐predictive at this stage. Thus, a larger sample size is required to provide sufficient evidence, so the trial enters an expansion phase with a further $n_{0,2}$ and $n_{1,2}$ patients recruited. A final analysis is then conducted with all accrued data both before and after interim analysis. At final analysis, if $P(D_{X}>0|\Omega_{1})>\eta_{2}$, the biomarker is declared predictive.

By only requiring the expansion when the evidence of a predictive effect is insufficient, resources are better managed. For example, when $P(D_{X}>0|\Omega_{0})$ is large enough to declare a predictive effect, not entering an expansion phase protects against recruiting further patients to the study when the interim data is already sufficient for declaring the biomarker as predictive. Similarly, when $P(D_{X}>0|\Omega_{0})$ is small, by not expanding the trial, the resources put into testing a non‐predictive biomarker are conserved, with patients potentially protected from ineffective treatments.

**FIGURE 1:**
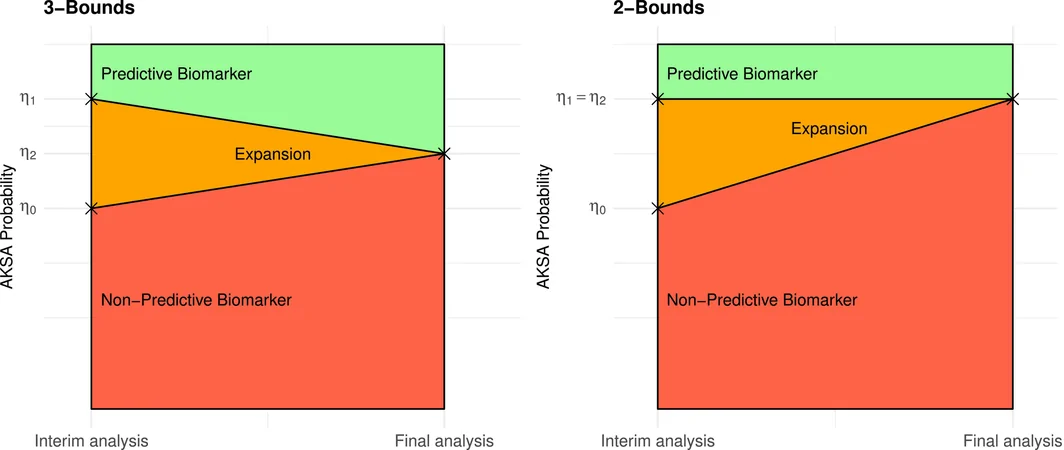
Decision windows based on the AKSA probabilities at interim and final analyses. These are defined by the decision bounds $\eta =\{\eta_{0},\eta_{1},\eta_{2}\}$, which are used to decide whether an expansion phase is required. In the ‘3‐Bounds’ plot, each of $\eta_{0}$, $\eta_{1}$ and $\eta_{2}$ are distinct, whereas, in the ‘2‐Bounds’ plot, $\eta_{1}=\eta_{2}$, that is, the bound to declare a predictive effect is fixed to be equal at both interim and final analysis.

The three thresholds (also referred to as decision bounds), $\eta =\{\eta_{0},\eta_{1},\eta_{2}\}$, must be carefully pre‐specified in order to control the Type I error rate without compromising power. Therefore, the η parameters are calibrated to control the Type I error, under a specified scenario, for example, when the true predictive effect is null. The thresholds play a vital role in the design of the study: if $\eta_{0}$ is set too high, a truly predictive effect could be missed; if it is set too low, then more resources are spent on a non‐predictive biomarker. Similarly, setting $\eta_{1}$ or $\eta_{2}$ too low will make it easier to declare a predictive effect, increasing the power but at the cost of inflated Type I error rates. Bounds that are too high will protect the Type I error rate but reduce the power of the study. Two potential decision bound configurations are considered and presented in Figure 1. The first option (presented in the right‐hand side of Figure 1) sets the bounds for declaring a biomarker as predictive at both interim and final analysis as equal (i.e., $\eta_{1}=\eta_{2}$). The second option (presented in the left‐hand side of the Figure) allows these two bounds to differ [26]. Setting the bounds to be equal provides a simple and consistent decision rule across analyses, reducing calibration complexity while making it easier to justify, communicate and implement. That being said, requiring the same bound at interim and final analysis despite the substantially different sample size (and thus evidence) may be too stringent and result in more conservative bounds, thereby restricting power [27]. By introducing differing bounds at interim and final analysis, the design is more flexible and allows for more or less stringent decision rules for declaring a predictive effect at both interim and final analyses. This flexibility can be harnessed to improve the balance between Type I error control and power gain.

### 3.3 Sample Size Considerations

While the calibration of η plays a role in maintaining acceptable operating characteristics for declaring predictiveness, the size of the expansion cohort, as well as, the planned interim sample size, also plays an important role.

The interim sample sizes are often driven by the PoC study and are thus typically fixed. Should these sample sizes be too small, there will be insufficient power to detect the predictive effect, if larger, power would increase with more trials terminating at interim without requiring an expansion phase to declare a predictive effect, thereby reducing the impact of the interim analysis. Also, due to the early‐phase setting, the initial PoC sample sizes are unbalanced in order to focus on gaining information on the experimental arm rather than the control arm. These initial sample sizes ($n_{1,1}$, $n_{0,1}$) have been selected to power the primary analysis to assess the treatment effect. The expansion phase, however, is aimed at assessing the predictive effect. Assessing the predictive effect requires information from both the experimental and control arm, with power maximised when sample sizes are balanced between the two. Therefore, allowing $n_{0,2}$ and $n_{1,2}$ to be unequal to balance the final sample size after expansion (such that $n_{0}=n_{1}$), will likely be the optimal approach. However, this balanced total sample size approach requires unequal allocation in the expansion phase in favour of the control arm, which can cause patients concern and ethical issues, limiting participation.

The sample size of the expansion cohort is also key. Trials that enter the expansion phase have shown insufficient evidence to claim the biomarker is predictive, however, there is also insufficient evidence to support that it is non‐predictive. This implies more data is required to make a decision regarding the predictive effect. By adjusting the expansion sample sizes $n_{1,2}$ and $n_{0,2}$, the total sample size of the trial, N will also change. A larger expansion phase will result in higher power to declare a predictive effect due to the increased total sample size. The decision bounds also play a part here, as the larger expansion sample sizes means that $\eta_{2}$ can be relaxed, making it easier to claim a predictive effect at final analysis. The decision bounds at interim can also be selected such that more trials enter the expansion phase (i.e., by increasing $\eta_{1}$ and decreasing $\eta_{0}$), this would increase the impact of the expansion cohort sample size of the final analysis.

Although the benefits of increasing the number of patients in the expansion phase are clear in terms of statistical power, this comes with financial burdens and resource constraints. Therefore, a smaller expansion phase may be required, leading to a reduction in power for identifying the predictive effect. This reduction in power at final analysis can be mitigated by ensuring more trials conclude the biomarker as predictive at interim (by decreasing $\eta_{1}$), which will increase the overall power of the study (i.e., detecting the predictive effect at *both* interim and final analysis), but could inflate the Type I error rate.

## 4 Simulation Study

### 4.1 Setting

In this section, several simulation studies are conducted to explore various interim analysis settings, including the choice of bounds, η, and the sample size configurations both before and after interim analysis. The purpose of these simulations is to demonstrate how each design choice impacts operating characteristics in relation to declaring predictiveness. This can be used to inform future designs.

The simulation studies are motivated by the setting described in Section 2, where patients outcomes are binary and the goal is to detect the predictive effect of the biomarker. The biomarker is assumed to be Gamma distributed with rate r=0.083 and shape $s=650.54r^{2}$. In the non‐adaptive motivating example, patients are randomised in a 2:1 allocation ratio in favour of the experimental arm, with a total of N=60 patients enrolled, thus, there are $n_{1}=40$ patients on the experimental arm and $n_{0}=20$ on the control arm. We now explore the impact of adding an interim analysis and potential expansion cohort to this study.

### 4.2 Simulation Scenarios & Data Generation

Simulations are conducted under a range of scenarios that vary the levels of prognostic, predictive and treatment effects. As in Serra et al. [6], the coefficients $b=\{b_{0},b_{1},b_{2},b_{3}\}$ in Equation (1) are selected to construct such scenarios. Given a biomarker value, X, response outcome Y and treatment indicator T, the predictive effect (defined as the difference in response between the experimental and control arm above and below a certain threshold, c) is computed as:

$$\begin{array}{ll} PE(b,c) & =\left[P(Y=1|T=1,X>c)-P(Y=1|T=0,X>c)\right]\\ & \;-\left[P(Y=1|T=1,X\leq c)-P(Y=1|T=0,X\leq c)\right], \end{array}$$

where c is the biomarker associated with the intersection between the response curves of the experimental and treatment arm. The response curves are constructed using the coefficients b. The equation PE(b,c) is then solved for various different levels of predictive effect, assuming the biomarker X ranges from 0 to 100.

In this work, the naming convention for each scenario follows that outlined in Serra et al. [6], where the predictive effect take values of 50%, 40%, 30% or 0%, these are labelled as H (high), M (medium), L (low) and N (none) predictive effects respectively. Thus, for a scenario with predictive effect of P%, the set of coefficients b, along with their associated cut‐off value c, are determined such that PE(b,c)=P%.

The prognostic effect is the difference in response rate above and below a threshold, c, within the control group. Thus, it is computed as follows:

PR(b,c)=P(Y=1|T=0,X>c)−P(Y=1|T=0,X≤c).

The prognostic effects are also denoted H high, M medium and N none and are fixed with respect to the predictive effect. The prognostic effect under the control is captured by the $b_{3}$ parameter in Equation (1), with $b_{2}$ reflecting the predictive effect. Thus, under H prognostic scenarios, $b_{3}$ is fixed such that $b_{3}=0.5b_{2}$, for the M prognostic scenarios, $b_{3}=0.2b_{2}$, and finally, for the N prognostic scenarios, $b_{3}=0$. When no treatment effect is present, $b_{1}$ is set to 0. The combination of b parameters for each simulation scenario are presented in Table 1 in Section 1 of the Supporting Information.

Note that in scenarios with no predictive but a prognostic effect, the response curves for the experimental and control arms do not intersect. In this case, the biomarker cut‐off value, c, cannot be defined as the intersection point and is therefore fixed at c=17 to correspond to an ORR of around 40% and 60% in the control and experimental arm respectively.

**FIGURE 2:**
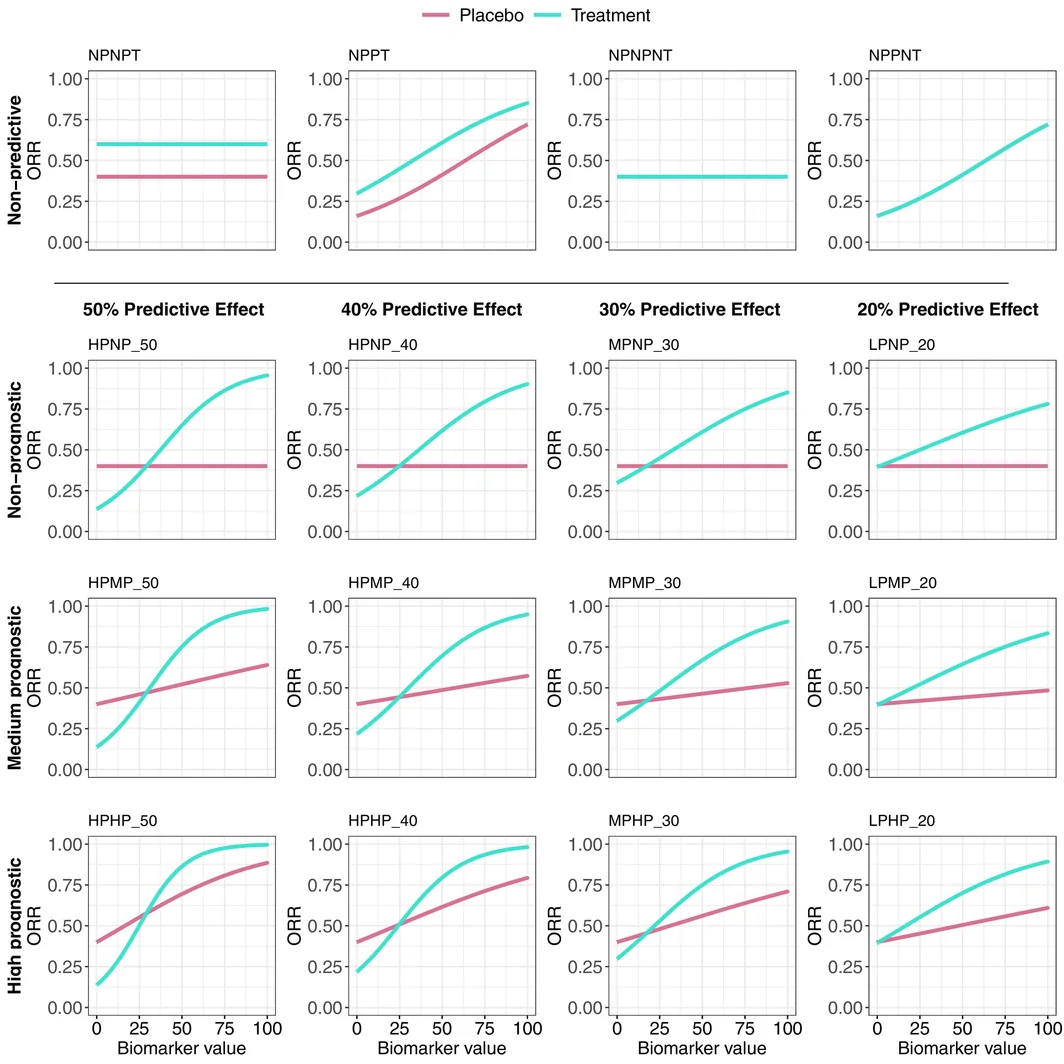
Simulation scenarios with varying predictive, prognostic and treatment effects. The first row of plots are the null scenarios in which no predictive effect is present. These scenarios are denoted: NPNPT (non‐predictive non‐prognostic with treatment effect), NPPT (non‐predictive prognostic with treatment effect), NPNPNT (non‐predictive non‐prognostic without treatment effect) and NPPNT (non‐predictive prognostic without treatment effect). The second row are scenarios with no prognostic effects, where columns 1–4 have a declining predictive effect from 50% down to 20%. Similarly, the third and fourth rows are scenarios with medium and high prognostic effects respectively with varied predictive effects.

The response curves for each of the considered simulation scenarios are presented in Figure 2. The four null scenarios, where there is no predictive effect are denoted as: NPNPT (non‐predictive non‐prognostic with treatment effect), NPPT (non‐predictive prognostic with treatment effect), NPNPNT (non‐predictive non‐prognostic without treatment effect) and NPPNT (non‐predictive prognostic without treatment effect). Other scenarios with a predictive effect are similarly named, for example, HPHP_50 denotes the high‐predictive high‐prognostic scenario with predictive effect of 50%, MPNP_30 denotes the medium‐predictive non‐prognostic scenario with predictive effect of 30% and LPMP_20 denotes the low‐predictive medium‐prognostic scenario with predictive effect of 20%.

For each scenario, once the parameters b have been obtained for the specified predictive and prognostic effects, data is generated by substituting b into Equation (1) where X is drawn from the pre‐specified biomarker distribution $\phi_{X}(x)$. The resulting data is then used to assess whether the biomarker demonstrated a predictive effect. Data generation is replicated R=5000 times for each simulation scenario and used to compute the trials operating characteristics. All simulations are conducted using R version 4.1.1 [28]. Results in the main text will focus on the null scenarios and the high predictive scenarios only, with simulation results for medium and low predictive scenarios presented in Section 3 of the Supporting Information.

### 4.3 Decision Bound Calibration

In order to make decisions regarding the predictive effect of a biomarker at both interim and final analysis, the AKSA probabilities are compared to three decision bounds: $\eta =\{\eta_{0},\eta_{1},\eta_{2}\}$. In this work, only a 3‐Bound configuration is considered (left‐hand side of Figure 1), where all three bounds, $\eta_{0},\;\eta_{1}$ and $\eta_{2}$ are distinct, allowing for different bounds for declaring a predictive effect at interim and final analysis. Despite the additional complexity of including an additional decision parameter, the 3‐Bound configuration allows for greater flexibility, permitting more or less stringent decision rules for declaring a predictive effect at both the interim and final analyses. Each decision bound is calibrated separately to control the trial's operating characteristics. Simulation results under a 2‐Bound configuration are provided in the Supporting Information.

The interim bound, $\eta_{0}$, is calibrated such that in at least 70%, 75%, or 80% of simulation runs, the biomarker is deemed non‐predictive at interim under the four null scenarios. These $\eta_{0}$ calibration rules are evaluated independently.

The Type I error rate is split equally across the interim and final analysis. This equal split was motivated by the operating characteristics observed under a 2‐Bound configuration, where a greater weight was placed on the Type I error at interim compared to final analysis (approximately 10% of the overall 15% Type I error spent at interim), which resulted in lower power. Different weights can be considered but they will yield different trade‐offs in terms of the operating characteristics. Allocating a greater proportion of the Type I error to the final analysis requires more stringent interim decision criteria, reducing the probability of early declarations of the predictive effect and increasing the number of trials proceeding to the expansion phase. Although this demands greater resources, this approach provides more reliable estimates of the predictive effect due to the larger sample size at the final analysis compared to the interim, while permitting less stringent decision criteria to declare success after expansion. Conversely, allocating a greater proportion of the Type I error to the interim analysis reduces the probability of requiring an expansion phase, and as a result, reduces the resources required. However, this places a greater reliance on the sparse interim data, which is fundamentally limited by the small sample size and risks erroneous conclusions. Therefore, $\eta_{1}$ is calibrated such that approximately 7.5% of simulation runs declare the biomarker as predictive at interim across the four null scenarios.

Once $\eta_{0}$ and $\eta_{1}$ have been calibrated, $\eta_{2}$ is calibrated under the “worst” null, that is, the scenario where Type I error is greatest, to ensure that the maximum Type I error rate under the four null scenarios remains at or below 15%.

Such calibrations are conducted separately for each trial setting considered. By calibrating the parameters to meet pre‐specified interim rules, the impact of the sample size on the trials operating characteristics can be isolated from the decision rules, allowing for a fair comparison between the designs.

### 4.4 Expansion Phase Sample Size Settings

Several different sample size settings are considered and evaluated through different simulation studies. In all settings, the interim sample sizes are fixed at those from the motivating example, with $n_{0,1}=20$ and $n_{1,1}=40$, while the expansion sample sizes are varied.

In the first set of simulations, an equal allocation ratio (1:1) to the experimental and control arm is implemented in the expansion phase such that $n_{0,2}=n_{1,2}$. Several expansion sample sizes are considered, each reflecting an earlier or later final analysis:

(2) $$n_{0,2}=n_{1,2}=\{10,15,20,25,30\}.$$

This balances the expansion sample sizes, however, the final sample sizes remain unbalanced, with more patients on the experimental arm.

In the second set of simulations, an unequal allocation ratio is considered, where the expansion sample sizes are selected to balance the total sample sizes such that $n_{0}=n_{1}$. The following expansion phase allocation ratios are considered:

0:20,5:25,10:30,15:35,20:40,

such that the total final sample sizes are:

N={80,90,100,110,120}.

For each of these expansion sample sizes, the sixteen simulation scenarios in Figure 2 are explored under the three calibration rules (70%, 75%, and 80%). Calibration occurs for each expansion phase sample size separately.

### 4.5 Simulation Metrics

In order to evaluate the trial's operating characteristics, under each of the design settings outlined in Section 4.4, several metrics are computed. These are computed under all 16 scenarios presented in Figure 2:

- Type I Error Rate: the percentage of simulation runs for which a non‐predictive biomarker is incorrectly declared as predictive at either interim or final analysis. This metric only applies for the null scenarios where the biomarker is set to be non‐predictive.
- Power: the percentage of simulation runs for which a truly predictive biomarker is declared as predictive at either interim or final analysis. This metric only applies for the non‐null scenarios, where the biomarker is set to have a high, medium or low predictive effect.
- % SNP: the percentage of simulation runs where the trial stopped at interim due to a lack of activity to demonstrate a predictive effect. The biomarker was declared as non‐predictive.
- % SP: the percentage of simulation runs where the trial stopped at interim due to sufficient activity demonstrating a predictive effect. The biomarker was declared as predictive without requiring an expansion phase.
- Average N: the average total sample size of the trial.
- PoS post: the probability of declaring a biomarker as predictive given that the trial continued to final analysis through the expansion phase [29]:
$$\text{PoS post}=\frac{\begin{array}{l} P(\eta_{0}\leq P(D_{X}>0|\Omega_{0})\leq \eta_{1}\cap \;P(D_{X}>0|\Omega_{1})>\eta_{2}) \end{array}}{P(\eta_{0}\leq P(D_{X}>0|\Omega_{0})\leq \eta_{1})}.$$

The goal is to maximise power and PoS post, while minimising Average N. Note that power can be written as:

$$\begin{array}{ll} \text{Power} & =\text{\% Declared predictive at interim analysis}\\ & \;+\text{\% Declared predictive at final analysis}\\ & =\text{\% SP + (\% Continue to expansion}\;\times \;\text{PoS post)}. \end{array}$$

The Type I error rate is controlled through calibration such that the maximum Type I error rate is 15% across the four null scenarios with no predictive effect. Similarly, under the null scenarios, % SNP will also be controlled through the pre‐specified calibration rules, such that the trial is stopped at interim and the biomarker declared non‐predictive in either: 70%, 75%, or 80% of cases. The % SP metric is also controlled at interim under the null scenarios to 7.5%. In the non‐null scenarios the goal is to minimise % SNP and maximise % SP, both of which contribute to the power.

These metrics have been selected to provide an overview of the trials behaviour both at interim and final analysis. The Average N metric will demonstrate the benefits in terms of resource management in implementing an interim analysis compared to a design with the same total sample size, N, without an interim applied. This is also reflected in the % SNP and % SP metrics. The concept of PoS post was proposed by Rondano et al. [29] to mitigate the tendencies for study teams to be too pessimistic or optimistic based on the interim evaluation. PoS post indicates the benefits that can be obtained through an expansion phase. If PoS post is too low, the expansion phase will do little to identify a predictive effect at final analysis. If PoS is high, then it quantifies that the expansion will identify a predictive effect with high certainty. This is important to consider when deciding whether an expansion phase will be beneficial, particularly when taking into account the resources required.

In addition, results for each setting are compared against two benchmarks: (i) the 40:20 sample size configuration (i.e., a trial without expansion based on the motivating example), and (ii) a study with the same total sample size given the expansion: $N=n_{0,1}+n_{1,1}+n_{0,2}+n_{1,2}$, but with no interim analysis conducted. While the latter benchmark may be less practical than expansion cohorts in real trials, which are usually triggered by interim results rather than planned automatically, it serves as a useful theoretical comparator for isolating the statistical impact of multiplicity introduced by the interim analyses. Paired with the above metrics, both benchmarks demonstrate the advantages and disadvantages of including the interim analysis and expansion cohort.

### 4.6 Simulation Results

Results under the four null scenarios and the high predictive scenarios are now provided for each of the settings outlined in Section 4.4. The results demonstrate how design choices impact inference in terms of declaring that a biomarker has a predictive effect at both interim and final analysis.

#### 4.6.1 Equal Allocation in Expansion Phase

The first set of simulation results implement an equal allocation in the expansion phase and consider various expansion sample sizes by setting and adjusting $n_{0,2}=n_{1,2}$. These sample sizes are varied from 10 up to 30. Also compared are the calibration rules, where the $\eta_{0}$ bound is calibrated such that % SNP at interim is either 70%, 75%, or 80% under the four null scenarios. The $\eta_{1}$ and $\eta_{2}$ bounds are then calibrated to ensure that the maximum of % SP across the same scenarios is 7.5%, with overall maximum Type I error rate of 15%, respectively.

**FIGURE 3:**
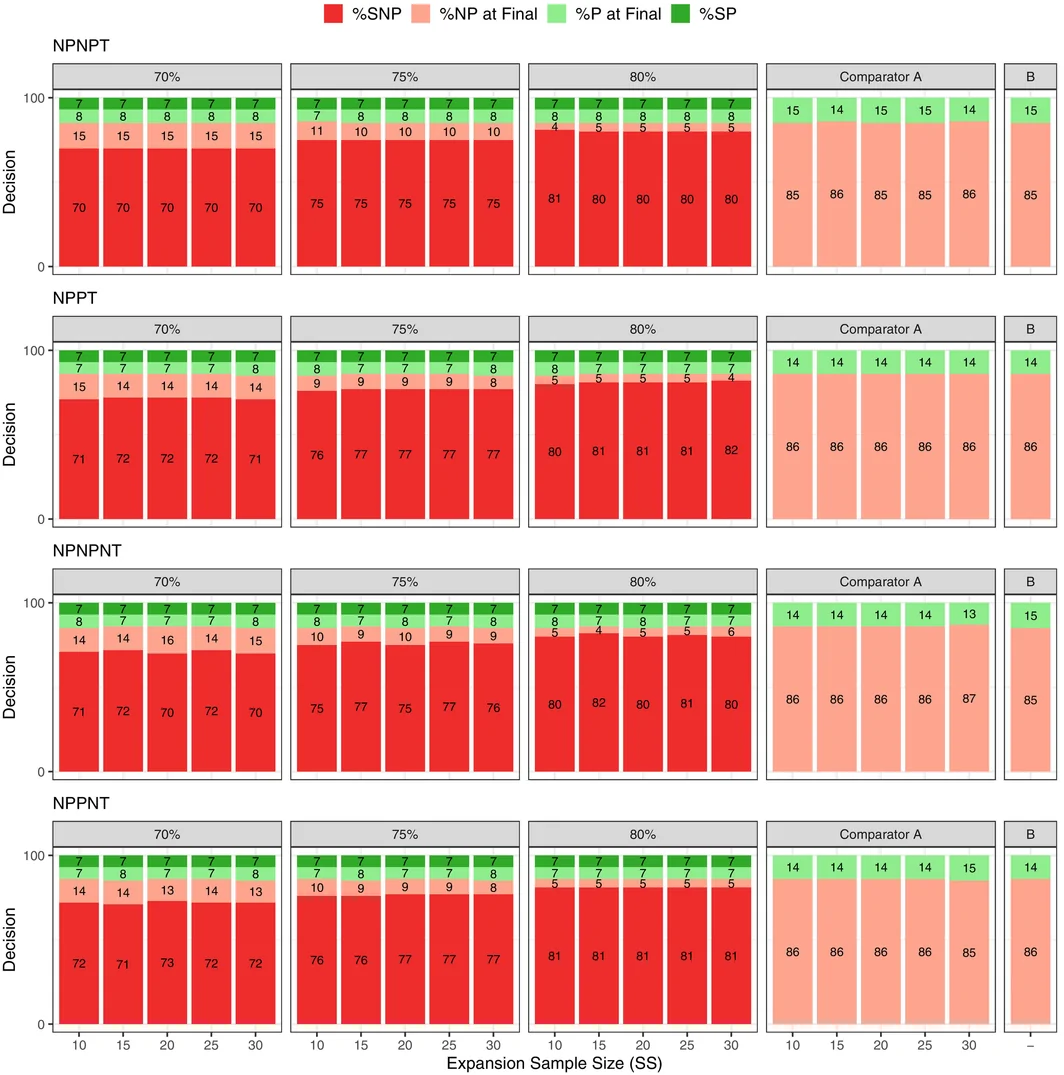
Percentage of simulations under the null scenarios (5000 replications) that resulted in each decision category: predictive (%SP, dark green) or non‐predictive (%SNP, dark red) at interim, or predictive (%P at Final, light green) or non‐predictive (%NP at Final, light red) at final analysis. Results are by expansion sample size (equal allocation) and calibration rule (70%, 75%, and 80%). Comparator A is the design with no‐interim but total sample size matched to the interim design. Comparator B is the no‐interim design with 40:20 allocation.

**FIGURE 4:**
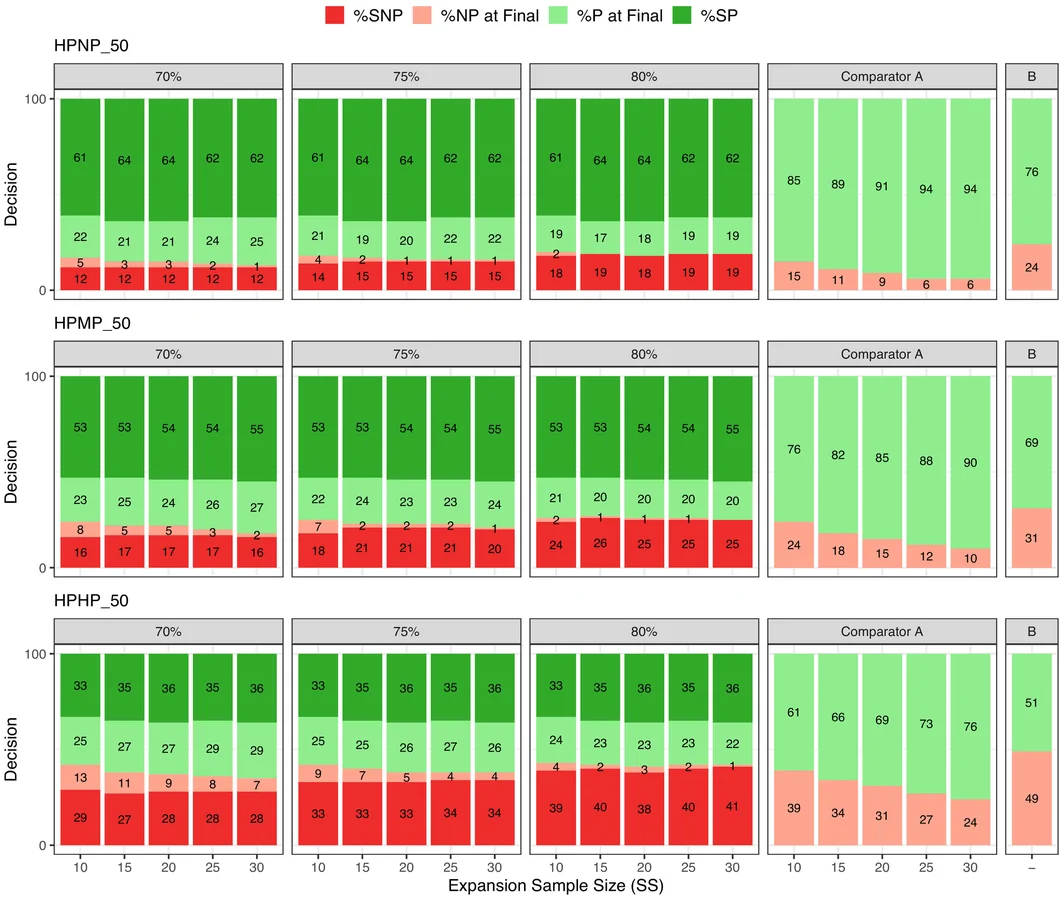
Percentage of simulations under the non‐null scenarios with 50% predictive effect (5000 replications) that resulted in each decision category: predictive (%SP, dark green) or non‐predictive (%SNP, dark red) at interim, or predictive (%P at Final, light green) or non‐predictive (%NP at Final, light red) at final analysis. Results are by expansion sample size (equal allocation) and calibration rule (70%, 75%, and 80%). Comparator A is the design with no‐interim but total sample size matched to the interim design. Comparator B is the no‐interim design with 40:20 allocation.

**FIGURE 5:**
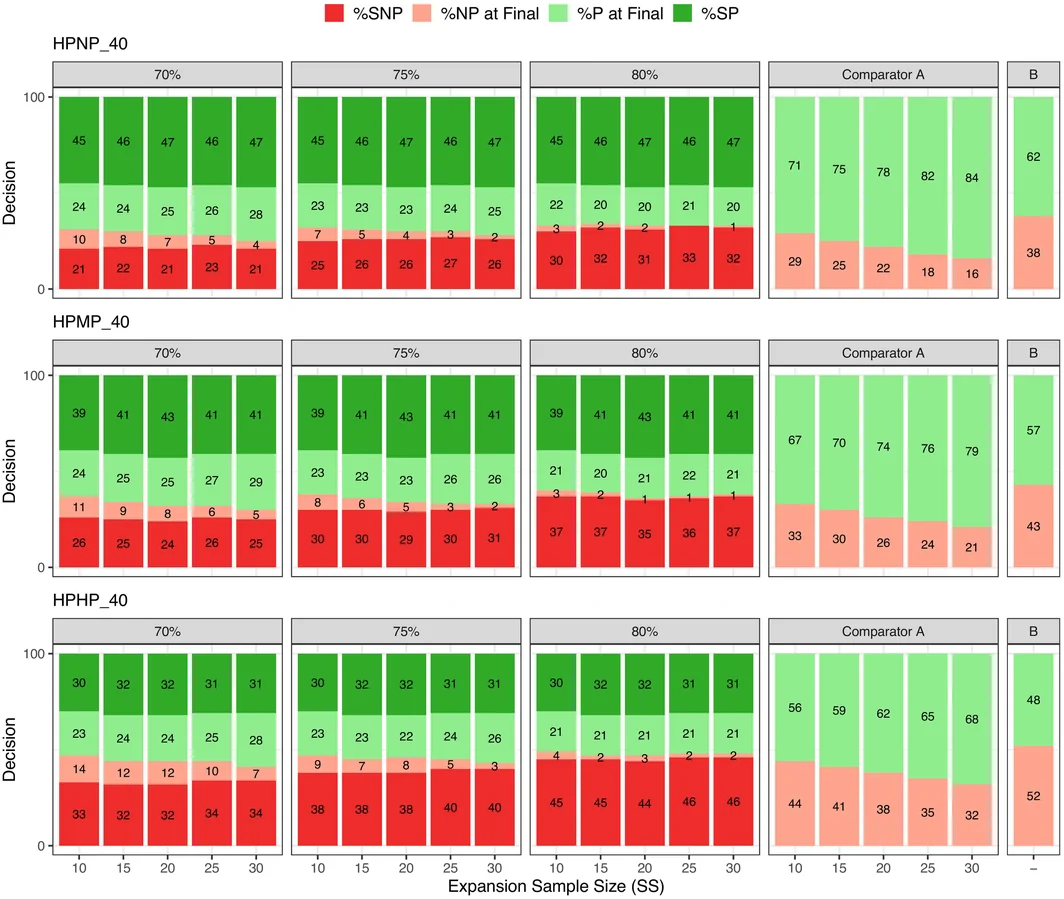
Percentage of simulations under the non‐null scenarios with 40% predictive effect (5000 replications) that resulted in each decision category: predictive (%SP, dark green) or non‐predictive (%SNP, dark red) at interim, or predictive (%P at Final, light green) or non‐predictive (%NP at Final, light red) at final analysis. Results are by expansion sample size (equal allocation) and calibration rule (70%, 75%, and 80%). Comparator A is the design with no‐interim but total sample size matched to the interim design. Comparator B is the no‐interim design with 40:20 allocation.

**FIGURE 6:**
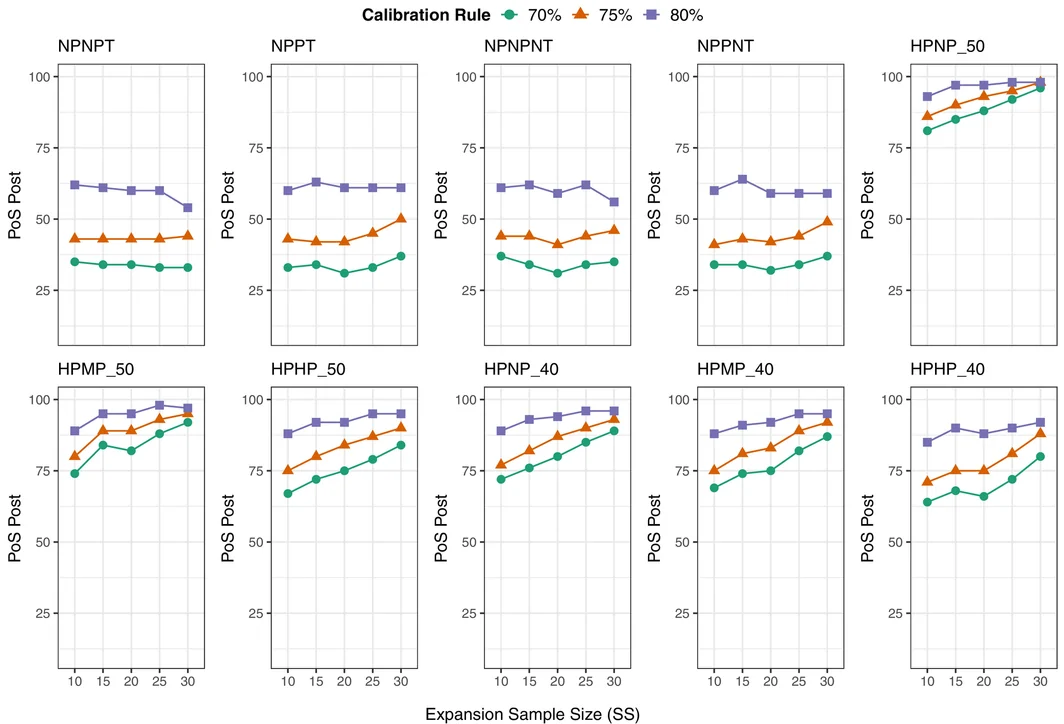
PoS post for the null and high predictive scenarios (5000 replications). Results are provided for three different calibration rules: 70%, 75%, and 80% and different expansion sample sizes (equal allocation): $n_{0,2}=n_{1,2}=10,15,20,25,30$.

The simulation results are provided in Figures 3, 4, and 5, which present the proportion of simulation runs that fall in each of the decision pathways: declaring a biomarker as predictive or non‐predictive at interim, or if the trial continues to expansion, declaring the biomarker as predictive or non‐predictive at final analysis (% P at Final and % NP at Final). This reflects the full range of possible trial outcomes across all design configurations considered. Each bar sums to 100%, providing an intuitive visualisation of how simulation runs are distributed across the decision pathways. When a true predictive effect exists, the sum of the green regions represents the power, that is, the probability of declaring a predictive effect, while the red zones represent a Type II error. When no predictive effect exists (i.e., the null scenarios), the sum of the green regions is the Type I error rate. Results are also provided for two designs with no interim analysis where (i) the total sample size is matched to that of the design with an interim analysis (Comparator A), and (ii) a 40:20 total sample size configuration (Comparator B). Figure 6 presents the ‘PoS post’ metric, while the average sample sizes are provided in the Supporting Information. Simulation results for the remaining medium and low predictive scenarios are presented in Section 3 of the Supporting Information, with calibrated decision bounds: $\eta_{0},\;\eta_{1}$ and $\eta_{2}$ provided in Section 2.

The decision bound, $\eta_{0}$, is calibrated for each combination of expansion sample size and % SNP calibration rule to ensure that the maximum Type I error rate is 15% for all designs under the four null scenarios. Consequently, in Figure 3, the Type I error remains at or below 15% under the null scenarios.

Under the non‐null scenarios in Figures 4 and 5 (which present results under the 50% and 40% predictive effect respectively), generally, the size of the expansion cohort has limited impact on power provided that the cohort has at least 15 patients in each arm. Power is highest when the expansion sample size is $n_{0,2}=n_{1,2}=25$, however, under a 70% calibration rule, across all high predictive scenarios, power varies by no more than 5% across the $n_{0,2}=n_{1,2}=15-30$ expansion sample sizes. This is likely due to the high % SP values, indicating that most of the power of the study arises from the interim analysis. For example, under the HPNP_50 scenario with a 70% calibration rule, 71%–75% of the total power comes from declaring the biomarker as predictive at interim. When considered alongside the % SNP values, only approximately 25% of simulations proceed to the expansion phase, this in itself restricts the influence of the expansion cohort size on overall power. The % SNP and % SP remain constant across the expansion sample sizes (with some variation from simulation noise), as the interim sample size is fixed at 40:20 for all designs. Nevertheless, power is noticeably reduced when $n_{0,2}=n_{1,2}=10$, where power is 3%–6% lower than the setting with $n_{0,2}=n_{1,2}=25$.

Power is consistently highest when there is no prognostic effect. When a prognostic is present, that is, when the biomarker influences the outcome regardless of the treatment, it becomes more difficult to distinguish the predictive effect from the baseline prognostic effect, thereby reducing the power of the study. Under the 50% predictive effect scenarios with a 70% calibration rule, the presence of a high prognostic effect leads to approximately 29% of simulation runs incorrectly declaring the biomarker as non‐predictive at the interim analysis, compared with around 12% when no prognostic effect is present. This can be attributed to greater standard errors of the interaction term when the prognostic effect is high, which make estimation of the predictive effect less accurate. For further details, see Section 3.4.4 of the Supporting Information.

The reduced power is accompanied by a reduction in the ability to detect a predictive effect at interim, with % SP decreasing from 64% to 35%. As expected, the magnitude of the predictive effect also impacts power, with a higher predictive effect of 50% being easier to detect compared to the smaller effect of 40%.

Under the non‐null scenarios in Figure 6, as the sample size of the expansion phase increases, so does the PoS post, reflecting the greater amount of information available once the trial continues into the expansion phase. This is reflected in the Average N metric, which naturally increases as the expansion becomes larger. Under the null scenarios, PoS post remains fairly consistent across expansion sample sizes. This is a consequence of the calibration procedure, where the decision bounds are chosen for each sample size to ensure that the Type I error rate does not exceed 15%. PoS post contributes to the Type I error, thus, influencing the calibration.

A more stringent calibration rule increases $\eta_{0}$, which in general lowers the power. As $\eta_{0}$ increases, % SNP also increases across all scenarios, implying that a larger proportion of truly predictive biomarkers are incorrectly classified as non‐predictive at interim. At the same time, the % SP is higher under more stringent calibration rules, This occurs because the decision bound $\eta_{2}$ do not need to be as conservative to retain the same Type I error under the null scenario (see Table 2 in Section 2 of the Supporting Information). As a consequence, the PoS post also increases as the calibration rule becomes more conservative, since fewer biomarkers with weak predictive signals make it beyond the interim analysis to the expansion phase. This in turn increases the probability of claiming a biomarker as predictive given that the trial continued to final analysis.

Compared to the design with no interim but with the same total sample size as the design *with* interim (for example, comparing the $n_{1}=50$ and $n_{2}=30$ design without interim to the $n_{0,2}=n_{1,2}=10$ expansion sample size setting), power is uniformly lower. This reduction arises because the interim analysis introduces an additional opportunity to incorrectly conclude that the biomarker is non‐predictive. At the interim stage, inference is based on smaller sample sizes, leading to greater uncertainty and consequently, a higher probability of erroneous decisions regarding the predictive effect than in a design where inference is made only once using the full data set. In contrast, when the interim design is compared to the 40:20 sample size configuration without an interim analysis, power is uniformly higher. This reflects the contribution of the additional cohort recruited in the expansion phase, which provides further information and strengthens the evidence for detecting a predictive effect.

As less stringent calibration rules have been shown to have higher power, going forward, only a 70% calibration rule is considered in the simulation studies. Results under alternative calibration rules are provided in the Supporting Information.

#### 4.6.2 Unequal Allocation in Expansion Phase—Balanced Total Sample Size

As demonstrated in the work by Serra et al. [6], balanced sample sizes across the experimental and control arm maximises the power to detect a predictive effect of a biomarker. However, in some trial settings it may not be ethical to allow equal allocation. In the previous simulation results, 40 patients were recruited to the experimental arm at the time of interim, with 20 on the control arm. Following the interim, equal randomisation was implemented between the experimental and control arms, which resulted in unbalanced final sample sizes, $n_{0}$ and $n_{1}$, for the control and experimental arms respectability. In the next set of simulations, the post‐interim allocation ratio is altered to ensure the final sample sizes are balanced, that is, $n_{0}=n_{1}$. To achieve this, unequal randomisation is required post‐interim analysis, with a greater proportion of patients allocated to the control arm during the expansion phase. Note that such a randomisation ratio favouring the control arm is unlikely to be acceptable to patients, however, it is considered as a scenario to isolate the effect of the expansion phase randomisation ratio and the final sample size balance on operating characteristics.

**FIGURE 7:**
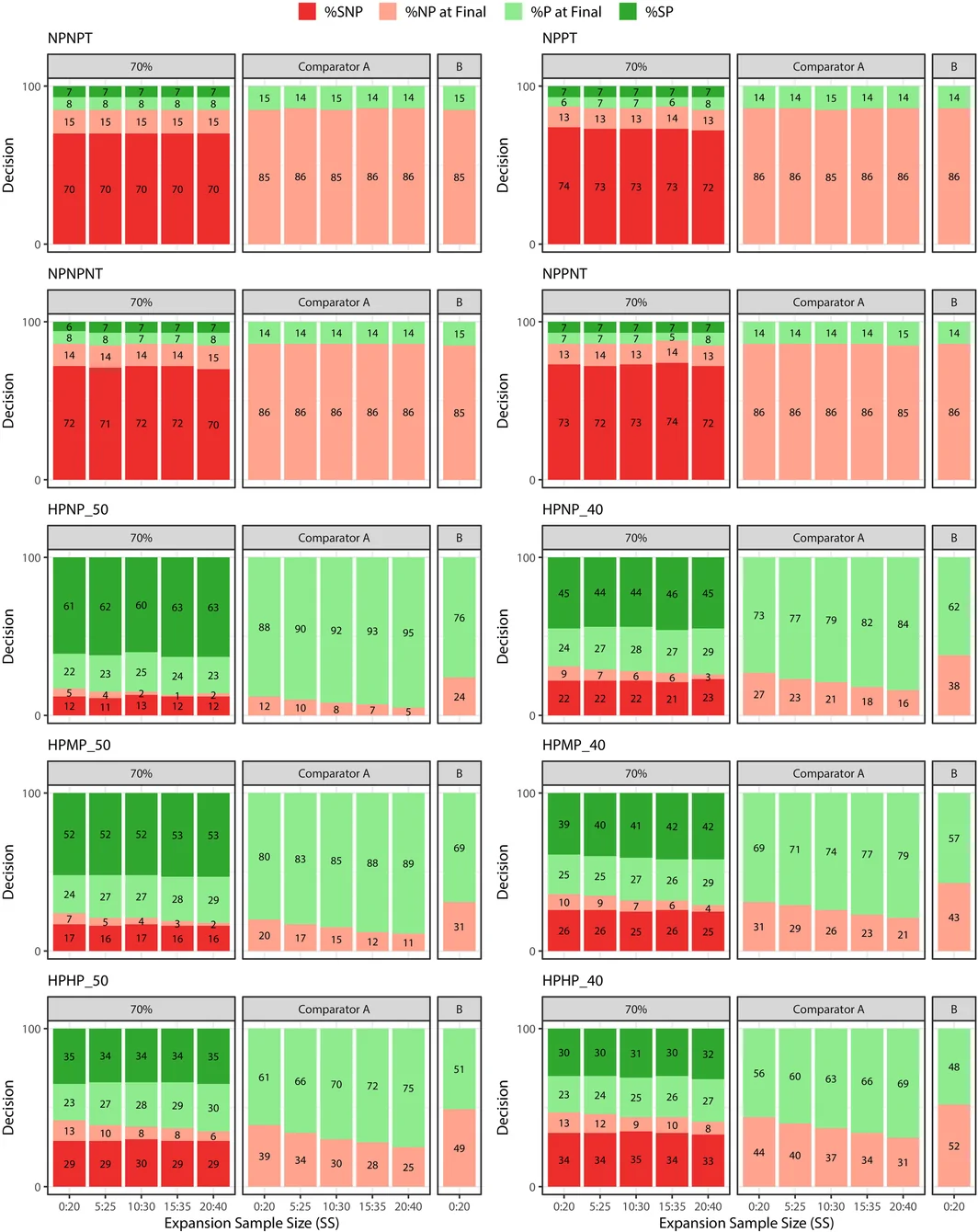
Percentage of simulations under unequal allocation (5000 replications) that resulted in each decision category: predictive (%SP, dark green) or non‐predictive (%SNP, dark red) at interim, or predictive (%P at Final, light green) or non‐predictive (%NP at Final, light red) at final analysis. Results are by expansion sample size, $n_{0,2}:n_{1,2}=0:20,10:30,15:35,20:40$, under a 70% calibration rule. Comparator A is the design with no‐interim but total sample size matched to the interim design. Comparator B is the no‐interim design with 40:20 allocation.

Results for power and Type I error under balanced final sample sizes and 70% calibration rule are provided in Figure 7, with results under alternative calibration rules provided in the Supporting Information. Alternative metrics are also provided in the Supporting Information. Compared to previous results with unbalanced total sample sizes, power is somewhat more sensitive to the expansion phase sample size, varying by up to 7% across the different total sample sizes in all high predictive scenarios. However, given that the total sample size varies by up to 40 patients, a more substantial difference may have been expected. This limited effect is largely due to the high values of % SP, which restricts the number of simulations proceeding to the expansion phase. For example, under HPNP_50, only 25%–27% of simulation runs continued to the expansion phase (with 61%–63% declaring a predictive effect at interim). This in itself limits the impact of the expansion sample size. Therefore, we expect minimal differences in operating characteristics between an equal and unequal allocation in the expansion phase.

#### 4.6.3 Overall Comparison

Comparisons are now drawn between the different settings: the three calibration rules and the unequal and equal expansion phase allocation ratio. Presented in Table 1 are the mean power, average N and PoS post taken across the six high predictive scenarios for each of the different considered settings. The mean Type I error across the four null scenarios are also provided for each setting. As the decision bounds: $\eta_{0}$, $\eta_{1}$ and $\eta_{2}$ have been calibrated separately for each setting, the Type I error rate is around 15%.

**TABLE 1:** Average power, total N, PoS post, % SNP and % SP metrics across the high predictive scenarios for both equal allocation and unequal allocation after expansion.

			Equal allocation ($n_{0,2}=n_{1,2}$)					Unequal allocation (N)				
Metric	Bounds	Calibration rule	10	15	20	25	30	80	90	100	110	120
Mean Type I error	3‐bound	80%	15	14	15	14	14	14	15	15	14	14
		75%	15	15	15	14	15	14	15	14	14	15
		70%	**15**	**15**	**14**	**14**	**15**	**14**	**15**	**14**	**14**	**15**
Mean power	3‐bound	80%	65	65	67	66	66	65	65	66	66	66
		75%	66	68	69	69	70	67	68	68	70	70
		70%	**67**	**70**	**70**	**71**	**73**	**67**	**69**	**70**	**71**	**73**
Mean average N	3‐bound	80%	65	67	69	71	73	65	67	69	71	73
		75%	66	68	71	74	76	66	69	71	74	76
		70%	**67**	**70**	**73**	**76**	**79**	**67**	**70**	**73**	**76**	**79**
Mean PoS post	3‐bound	80%	89	93	93	95	96	91	92	94	95	96
		75%	77	83	85	89	93	80	83	89	89	92
		70%	**71**	**77**	**78**	**83**	**88**	**72**	**77**	**82**	**83**	**87**
Mean % SNP	3‐bound	80%	32	33	32	33	33	33	33	33	33	33
		75%	27	27	27	28	28	28	27	28	28	27
		70%	**23**	**23**	**22**	**23**	**23**	**23**	**23**	**24**	**23**	**23**
Mean % SP	3‐bound	80%	44	45	46	45	45	44	44	44	45	45
		75%	44	45	46	45	45	44	44	44	45	45
		70%	**44**	**45**	**46**	**45**	**45**	**44**	**44**	**44**	**45**	**45**

*Note:* Mean Type I error rate under the null scenarios for the same settings. Three calibration rules are considered in which $\eta_{0}$ is calibrated such that 80%, 75%, or 70% of simulation runs under the null scenarios stopped at interim and declared the biomarker non‐predictive. The preferred approach is highlighted in bold, which balances power gain and an increase in PoS post‐interim.

Focusing on mean power, the results are largely insensitive to the post‐interim allocation ratio. Across studies with equal total sample sizes but different allocation ratios, the difference in mean power is no more than 6% across expansion sample sizes. This implies that the sample size at interim plays a far greater role in detecting a predictive effect than the final total sample size or allocation ratio. In other words, the interim analysis provides the majority of the evidence needed to establish a predictive effect, which explains the consistently high % SP across all simulations. Considering the % SNP and % SP values, the majority of simulation runs don't end up proceeding to final analysis, terminating at interim before expansion. This in itself limits the impact of the post‐interim allocation ratio. Nevertheless, additional patients recruited after interim do contribute information into the analysis which results in a small increase in power as the expansion sample size grows.

The results demonstrate that as the randomisation ratio has little impact on the operating characteristics of the proposed framework, there is no statistical justification for favouring the control group in the expansion phase. Instead, an ethically preferable equal allocation in the expansion phase can be implemented without compromising statistical power and Type I error control.

Calibration rules also have a notable impact on the trial's operating characteristics. More conservative calibrations increase the % SNP by approximately 10%, with no change in % SP. Consequently, power is reduced by up to 7% under a 80% calibration rule compared to a 70% calibration rule. This reduction in power occurs alongside only a modest increase in the average total sample size, with around 4 additional patients per trial. The mean PoS post is also substantially higher under more conservative calibration rules (8%‐18% higher under an 80% calibration rule vs. a 70% calibration rule). This aligns with the higher % SNP, where fewer trials with weaker predictive effects proceed to expansion, thereby increasing the probability of successfully declaring a predictive effect.

Based on the increases in mean PoS post and power, at least 30 patients in the expansion cohort is recommended (either 15:15 under equal allocation or 5:25 under unequal allocation). Given that a larger proportion of patients are assigned to the experimental arm with minimal impact on power, equal post‐interim allocation is preferred. In addition, a more relaxed 70% calibration rule enhances power and is thus recommended. This preferred setting is highlighted in bold in Table 1.

### 4.7 Sensitivity Analysis

In the previous set of simulation results, several assumptions were made regarding the setting. This includes both the interim sample sizes $n_{0,1}=20$ and $n_{1,1}=40$, the 2:1 initial randomisation ratio, and the distribution of the biomarker values: a ‘less skewed’ Gamma distribution. The sensitivity of the trials operating characteristics to these assumptions is now investigated.

#### 4.7.1 Interim Sample Sizes

In the previous simulations, the interim sample sizes were unbalanced in favour of the experimental arm (based on the motivating example). The purpose of this simulation study is to demonstrate that, while the expansion phase sample size had little to no impact on operating characteristics, the pre‐interim/initial sample size plays a major role. In this exploration, the interim sample size of the experimental arm remains fixed at $n_{1,1}=40$, while the interim sample size of the control arm is varied. Several options for $n_{0,1}$ are considered:

$$n_{0,1}=\{20,30,35,40,50\},$$

where $n_{0,1}=20$ is the previous setting considered. All other settings increase the sample size of the control arm, including $n_{0,1}=40$, where the sample size for the experimental and control arm are balanced at interim. Given the almost identical results observed under equal and unequal post‐interim allocation rations, for each of these interim sample sizes, each of the equal expansion sample sizes in (2) are explored based on ethical considerations. Similarly, a 70% calibration rule is implemented.

Average power, total sample size and PoS post across the high predictive scenarios are provided in the left‐hand side of Table 2 for different interim and expansion sample sizes. Mean power is highly sensitive to the choice of interim sample size for the control arm, increasing by 10%–12% when $n_{0,1}=20$ compared with $n_{0,1}=50$. When the interim sample size is balanced across the two arms, a substantial increase in power of 7%–10% is observed relative to the previously considered setting. However, the impact of the allocation ratio is not isolated from the impact of the total sample size. The balanced sample allocation across the two arms results in a total sample size of $n_{0}=90$, compared to the initial $n_{0}=60$. This drives the improvement in power as more reliable interim conclusions can be drawn based on the additional information.

**TABLE 2:** Average metrics across the high predictive scenarios under various interim sample sizes, $n_{0,1}$, interim allocation ratios, $n_{1,1}:n_{0,1}$, and biomarker distributions, with equal allocation in the expansion phase.

		Post‐interim SS						Post‐interim SS						Post‐interim SS				
Metric	$n_{0,1}$	10	15	20	25	30	Distribution	10	15	20	25	30	$n_{1,1}:n_{0,1}$	10	15	20	25	30
Mean power	20	67	70	70	71	73	Less‐skewed gamma	67	70	70	71	73	50:10	57	58	60	61	61
	30	74	74	77	77	78	Medium‐skewed gamma	69	70	73	74	75	40:20	67	70	70	71	73
	35	74	76	78	79	81	Skewed gamma	69	70	71	73	72	30:30	68	72	72	73	76
	40	77	80	79	80	80	Uniform	80	81	83	83	84	20:40	54	55	56	58	59
	50	78	81	81	83	83												
Mean average N	20	67	70	73	76	79	Less‐Skewed Gamma	67	70	73	76	79	50:10	66	70	73	77	79
	30	76	79	83	87	89	Medium‐skewed gamma	66	69	72	76	80	40:20	67	70	73	76	79
	35	82	84	88	92	95	Skewed gamma	66	69	71	75	76	30:30	68	71	75	80	83
	40	86	90	93	96	98	Uniform	65	68	70	73	77	20:40	67	70	74	78	81
	50	96	99	103	106	109												
Mean PoS post	20	71	77	78	83	88	Less‐skewed gamma	71	77	78	83	88	50:10	74	80	87	89	91
	30	73	76	80	83	88	Medium‐skewed gamma	72	78	82	86	89	40:20	71	77	78	83	88
	35	71	75	81	84	87	Skewed gamma	69	75	79	85	87	30:30	69	76	80	74	88
	40	71	79	79	83	84	Uniform	77	85	90	90	93	20:40	68	76	79	84	87
	50	70	79	80	85	88												
Mean % SNP	20	23	23	22	23	23	Less‐skewed gamma	23	23	22	23	23	50:10	32	33	33	32	32
	30	18	18	17	17	17	Medium‐skewed gamma	23	23	22	22	22	40:20	23	23	22	23	23
	35	16	16	15	15	14	Skewed gamma	22	22	23	22	24	30:30	20	19	20	21	19
	40	14	13	14	14	15	Uniform	14	14	14	14	14	20:40	33	34	34	33	34
	50	12	12	12	12	13												
Mean % SP	20	44	45	46	45	45	Less‐skewed gamma	44	45	46	45	45	50:10	36	35	34	35	36
	30	51	51	51	50	51	Medium‐skewed gamma	46	47	48	47	46	40:20	44	45	46	45	45
	35	50	53	52	51	52	Skewed gamma	49	49	49	48	49	30:30	43	44	43	41	42
	40	54	55	54	53	55	Uniform	60	59	60	60	58	20:40	33	33	31	32	32
	50	57	57	55	56	56												

Furthermore, as the interim sample size, $n_{0,1}$, increases, the proportion of simulation runs that identify the predictive effect at interim rises by approximately 12%, while the percentage of simulation runs incorrectly declaring the biomarker as non‐predictive nearly halves from 23% down to 12%. Despite this, minimal differences are observed in the mean PoS post, although it continues to increase with the expansion sample size, consistent with prior observations.

As in previous results, the impact of the expansion sample size remains minimal, particularly when the expansion consists of at least 30 patients. In this case power increases by no more than 5%. As seen previously, most of the power comes from the interim analysis, with high % SP, thus the post‐interim sample size has minimal impact.

While this sensitivity analysis focuses on explicitly increasing the information in the control arm at interim through increased sample sizes, the simulation results also highlight the potential impact of incorporating external or historic control data into the study as an alternative source of information at interim. Such borrowing could provide additional control information without increasing required recruitment, thereby limiting patient burden whilst still improving power.

#### 4.7.2 Interim Allocation Ratio

While the simulation study in Section 4.7.1 explored allocation to the treatment arms, the main purpose of the sensitivity analysis was to explore the impact of the pre‐interim/initial sample size. Thus, the total pre‐interim sample size was varied, meaning the impact of the allocation ratio could not be isolated. A further sensitivity analysis was conducted to isolate this impact.

In this exploration, the total interim sample size is fixed with $n_{0}=60$ patients, while both $n_{0,1}$ and $n_{1,1}$ (i.e., the interim sample size in the control and treatment arms respectively) are altered. Four different allocation ratios are considered:

$$n_{1,1}:n_{0,1}=\{50:10,\;40:20,\;30:30,\;20:40\}.$$

All other simulation settings are unchanged from the previous simulation studies, with varied but equal expansion sample sizes from 10 up to 30 and a 70% calibration rule. The 40:20 allocation ratio aligns with our previously considered setting in Section 4, 30:30 enforces equal allocation across both treatment arms, 50:10 places the majority of patients on the treatment arm, with few on the control arm, and finally, 20:40 is the less ethically appropriate setting that favours the control arm over the experimental arm.

The simulation results are presented in the right‐hand side of Table 2, which displays the average metrics across the high predictive scenarios. As expected, highest power is achieved when there is equal balance across the control and experimental arm, however, implementing a 2:1 allocation ratio in favour of the control arm results in only a 1%–3% reduction in power, while proving more favourable and attractive to patients. The 40:20 sample size also reduces the number of patients on the trial by up to 4 compared to the 30:30 allocation, as more trials are terminate at interim.

Favouring allocation to the control arm produces the lowest power, highest % SNP and lowest % SP, thus, would never be recommended from a statistical or ethical perspective. Similarly, allocating nearly all patients to the experimental arm (50:10 allocation) also reduces power by up to 15% compared to equal allocation and 12% compared to the 40:20 allocation implemented in the motivating example. The 50:10 allocation does have the highest mean PoS post as far more trials terminate at interim and incorrectly conclude a non‐predictive effect, meaning only trials with a high predictive signal proceed to final analysis.

Overall, these findings demonstrate that the allocation ratio prior to the interim does influence operating characteristics of the trial. While perfect balance between the treatment arm maximises power, the 40:20 allocation implemented in the motivating example and simulation studies offers a favourable compromise, sacrificing only a small amount of power, while reducing the average sample size and proving ethically favourable to patients. This is an important finding, as it suggests that when the allocation ratio is primarily driven by the primary endpoint, such as unbalanced allocation for the incorporation of external data, the impact on the predictiveness assessment remains limited provided the imbalance is not too extreme (e.g., a 50:10 allocation).

#### 4.7.3 Biomarker Distribution

In the previous simulations, the biomarker distribution, $\phi_{X}(x)$, was assumed to be Gamma with the shape and rate parameters selected such that distribution was less skewed, with more mass around central biomarker values: r=0.083, $s=650.54r^{2}$. Alternative biomarker distributions are now explored:

1. Gamma distribution: X∼Γ(r,s) with $\phi_{X}(x)=\frac{r^{s}}{\Gamma (s)}x^{s-1}\exp(-rx)$, where r is the rate, s is the shape and Γ(·) is the gamma function. The shape parameter, s, is selected to be a function of the rate parameter, r, such that $s=650.54r^{2}$. Several rate parameters were considered to provide different ‘shaped’ biomarker distributions.
  · Skewed Gamma: r=0.049.
  · Medium‐Skewed Gamma: r=0.069,
  · Less‐Skewed Gamma: r=0.083
2. Uniform distribution: X∼Uniform(a,b) with a=0 and b=100, where $\phi_{X}(x)=\frac{1}{b-a}$ for a≤x≤b and $\phi_{X}(x)=0$ otherwise.

**FIGURE 8:**
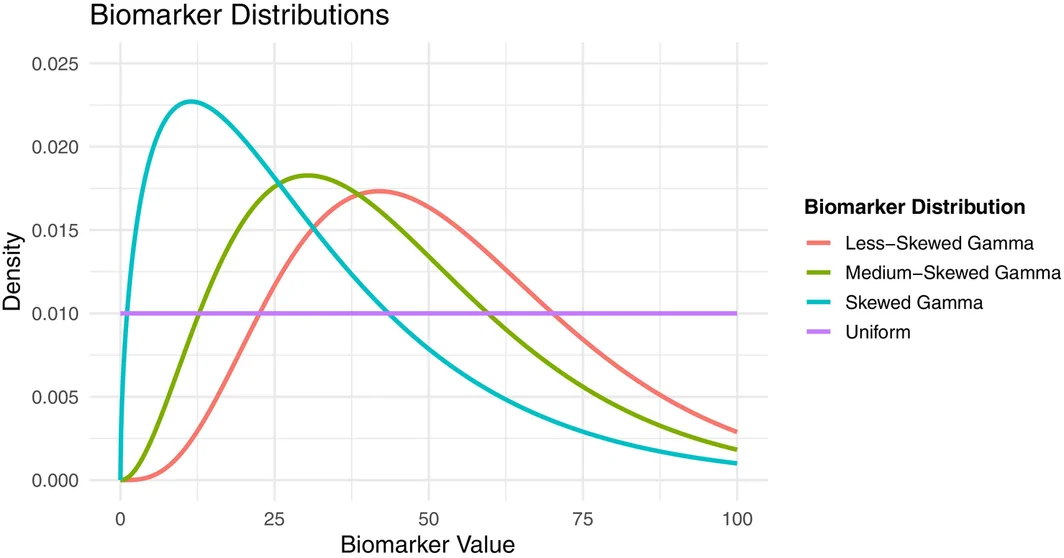
The Gamma and Uniform distributions considered for the continuous biomarker.

The skewed Gamma distribution puts more mass around smaller biomarker values compared to the less skewed distribution. The uniform distribution has each biomarker value equally likely between 0 and 100. Note that, while uniformly distributed biomarker values are less likely to occur in practice, evaluating the framework under this setting is important for methodological exploration. In early‐phase clinical trials, the true biomarker distribution is typically unknown. Because the uniform distribution represents a useful contrasting scenario to the assumed base‐case (Gamma distributed biomarker), it serves as a valuable benchmark to assess the robustness of the proposed framework, while isolating the impact of well‐spread versus concentrated biomarker values. All of the biomarker distributions considered in this Section are displayed in Figure 8.

The simulation study is repeated under a 70% calibration rule, where each of the biomarker distributions is evaluated in conjunction with each of the expansion sample sizes in (2). Average metrics across the high predictive scenarios are presented in the centre of Table 2. For the Gamma distributed biomarker, power is fairly insensitive to the degree of skewness of the distribution. However, more skewed distributions lead to up to a 5% increase in % SP, with a similar number of simulation runs incorrectly declaring the biomarker as non‐predictive at interim. With this, both the average total sample size and mean PoS post decrease slightly, as fewer simulations proceed to the expansion phase.

When the biomarker follows a uniform distribution, with values equally likely across the 0–100 range, power is considerably higher than for a Gamma distributed biomarker. Specifically, compared with a less‐skewed Gamma distribution, power increases by up to 13% for the uniform distributed biomarker. With this, mean PoS post increases by up to 12%, mean % SP increases by up to 15%, and % SNP decreases by 9%. The average sample size remains fairly stable across biomarker distributions. This pattern is expected: a uniform biomarker distribution provides information across the whole range of values, whereas a Gamma distributions leaves some regions of the biomarker range sparse. An accurate assessment of a predictive effect requires information spanning the full biomarker range, explaining the higher power and PoS post observed under the uniform distribution.

## 5 Discussion

In this work, a framework was proposed in order to make an assessment of the predictive effect of a continuous biomarker through an adaptive design, with a particular focus on the early‐phase setting where data is limited. This bridges an important methodological gap. While existing adaptive designs (such as adaptive enrichment or threshold refinement) primarily focusing on modifying the design based on observed biomarker data, the proposed framework explicitly targets formally evaluating the predictive effect of a biomarker. Such early‐stage evidence of predictive biomarkers is a critical component of efficient clinical development, as it directly informs subsequent trial design, patient selection strategies, and go/no‐go decisions, thereby reducing uncertainty and enabling more targeted and resource‐efficient development pathways.

The proposed interim framework allows for the inclusion of an expansion cohort, but only when the accumulated data indicates that additional information is required to reliably assess the predictive effect. In cases when the expansion is not required, that is, when there is already sufficient evidence for the presence of a predictive effect or when there is a clear lack of a predictive signal, the trial is completed without the additional patients recruited. As observed in the simulation results, it is expected that the majority of trials will stop at interim, without conducting an expansion phase. For example, under the null scenarios with no true predictive effect, with a 70% calibration rule and 3 distinct decision bounds, when the expansion sample sizes are equal with 15:15 allocation, 77%–79% of simulation runs completed the trial at interim, claiming either a predictive or non‐predictive effect, meaning only a minority (21%–23%) proceed to expansion. Only 7% of simulation runs incorrectly claim a predictive effect at interim under these scenarios. Similarly, under the same setting but with a high predictive signal, 62%–76% of simulation runs do not continue to the expansion phase, with 32%–64% of simulation runs correctly identifying the true predictive effect at interim. This highlights the efficiency of the interim analysis, as most decisions regarding the predictive effect of a biomarker can be made without the additional resources of the expansion phase. The interim analysis therefore provides important practical advantages in early‐phase biomarker studies, including improving power for identifying truly predictive or non‐predictive biomarkers through the optional expansion cohort, thereby allowing for better resource management and earlier evidence for adapting clinical strategy and development. This in turn will reduce patient burden when a predictive signal is not present (e.g., patients would not need a biopsy if the biomarker is not predictive enough to justify it), as the trial has approximately a 70%–73% probability of declaring the biomarker non‐predictive at the interim when no true predictive effect is present.

For trials that do proceed to the expansion phase, the added value of the expansion can viewed in terms of improved sensitivity to detect smaller predictive effects. In the setting with a 70% calibration rule, the interim sample size of 60 patients provides reasonable power to detect a high predictive effect when no prognostic effect is present, achieving approximately 64% power for a 50% predictive effect with a 15:15 expansion cohort. However, the same interim analysis is substantially underpowered for smaller predictive effect sizes, with 46% power to detect a 40% predictive effect, 35% power to detect a 30% predictive effect and just 21% power to detect a 20% predictive effect. The inclusion of an expansion cohort meaningfully addresses this limitation, increasing power to 70% (+24%), 58% (+19%) and 41% (+11%) for the three effect sizes, respectively. Therefore, the expansion enables detection of predictive effects that would otherwise be missed at interim. The interim analysis approach improves overall power of the study compared to designs without an expansion cohort, that is, a trial that stops recruiting at the initial 40:20 sample size. In contrast, a single‐stage design with matched total sample size as the 2‐stage design but with no interim was shown to maximise statistical power. This is because there is no risk of erroneously concluding a predictive biomarker as non‐predictive at interim. However, this approach does not yield the same efficiency advantages in terms of the early assessment of the predictive effect. As a result, the simulation studies under this design required, on average, 13–41 more patients across the different expansion sample sizes compared to the proposed 2‐stage design. This demonstrates the important trade‐off in power and operational efficiency. Furthermore, a single‐stage design is not practically realistic within our motivating PoC setting, where the interim analysis corresponds to the end of the initial ORR‐driven PoC study. The practical question is then whether to recruit additional patients to further evaluate the biomarker's predictive effect.

However, the feasibility of implementing an expansion cohort will depend on several clinical factors. For example, in some early‐phase studies, recruitment may be challenging due to small patient populations, financial resources may be limited and time‐frames given slow recruitment may not allow for an expansion phase. However, early clinical development should be viewed as a continuum of uncertainty reduction. Therefore, the primary consideration is whether the interim data provides sufficient evidence to justify further investigation of the biomarker. If it does, the remaining uncertainty must then be addressed either through an expansion cohort within the current study or by deferring biomarker evaluation to a subsequent trial with an appropriate design. If an expansion cohort is conducted, an additional consideration is how many patients should be recruited to reduce the remaining uncertainty. These decisions must be balanced against the practical constraints of implementing an expansion cohort. Foregoing the expansion could result in a failing to investigate a treatment that is highly beneficial in a specific subpopulation (which may not be apparent in the broader Phase I/II population). This is a significant missed opportunity. While expanding the trial requires additional time and resources, it mitigates the risk of failing to identify a subpopulation that responds to the treatment. Ultimately, the expansion could positively improve patient outcomes.

Where recruiting an expansion cohort is practically and operationally challenging, incorporating an additional interim analysis during the expansion phase may provide a compromise. The additional interim would allow the study to stop early once sufficient evidence in favour or against a predictive effect.

All simulation studies presented in this work considered a single interim analysis leading to an expansion phase. This was a design choice based on the motivating example, not a limitation to the methodology. As the proposed framework is akin to a group sequential design, it can accommodate any number of interim analyses. Given the small sample size setting, including an additional interim earlier in the study (i.e., before expansion) may lead to unstable estimates of the predictive effect, thereby producing unreliable predictiveness conclusions. In contrast, including an additional interim in the expansion phase may pose efficiency advantages, allowing for an earlier assessment of the predictive effect. This also offers sponsors greater flexibility, allowing patients to be recruited sequentially into the expansion phase and enabling a determination of whether robust biomarker conclusions can be reached earlier, rather than committing to the full expansion sample size upfront. In the Supporting Information, an additional simulation study compared a single interim design and a multiple interim design with an additional interim in the expansion phase. To summarise the findings, the additional interim reduced the average sample size by up to 6 patients, without a substantial loss in power. While these findings would encourage the incorporation of additional interim analyses, it must be weighed up with the additional operational complexity it may pose. For example, each interim requires data collection and analysis, as well as decision‐making before recruitment can proceed. This could increase trial durations, administrative burden and financial costs. Statistically, each interim requires additional decision parameters that must be calibrated.

Various design considerations are explored within the interim analysis framework, including various sample size settings and decision bound configurations to assess the predictive effect. All design considerations were investigated using simulation studies motivated by the NSCLC trial, where the power, Type I error and interim decisions were evaluated under a variety of quantitatively different data scenarios in which the prognostic and predictive effects varied. In order to assess a predictive effect, AKSA was implemented at both interim and final analysis. This method was selected given the reliable performance demonstrated by Serra et al. [6]—both in terms of maximising power and controlling the Type I error—for detecting a predictive effect in the small‐sample setting in comparison to alternative approaches such as the likelihood ratio or interaction test.

The operating characteristics of AKSA in the interim analysis framework are controlled through calibration of the three decision bounds η. Based on findings from the simulation studies in the Supporting Information, it is recommended to use three distinct decision bounds, allowing for differing thresholds for claiming a predictive effect at both the interim and final analyses. This improves power compared to fixing the predictiveness bounds at interim and final analysis ($\eta_{1}=\eta_{2}$), while controlling the allocation of Type I error rate across both stages. Similarly, a more stringent calibration rule of 70% (i.e., requiring 70% of simulation runs to terminate at interim and declare the biomarker as non‐predictive, when the biomarker is truly non‐predictive) is recommended. However, like other design recommendations, the optimal choice of calibration rule is context‐dependent and should be considered on a case‐by‐case basis, taking into account both the strength of the biomarker hypothesis, informed by external evidence and biological rationale, and the feasibility of recruiting additional patients into an expansion phase. A more stringent rule, such as the 70% calibration recommended from the simulation studies, may be preferable when the biomarker hypothesis is well‐supported and recruitment in the expansion phase is feasible, as it places greater weight on the more reliable final analysis. Overall, it is important to meticulously calibrate key design parameters through rigorous simulation studies, to ensure that these selections balance power and Type I error considerations.

In this work, the sample size at both the interim and final analyses were explored, and the impact on operating characteristics investigated. It was shown that, in the considered setting, the expansion sample size has minimal impact on operating characteristics, provided that at least a further 15 patients were recruited in each arm (i.e., a minimum of 30 patients total). This sample size threshold is motivated by the need to achieve a minimum level of additional statistical power to detect a predictive effect. According to these simulations, expansion cohorts smaller than 30 patients raise questions about their utility, given the limited gains in power. Similarly, implementing unequal recruitment in the expansion phase to balance the total sample size across the experimental and control arms was shown to have minimal difference to the operating characteristics compared to equal allocation across the two arms. Therefore, balanced allocation is ethically preferable, limiting the number of patients exposed to the control treatment.

While the expansion sample size has little effect on the operating characteristics, the interim sample size and allocation ratio has a substantial influence on the power of the study in the considered PoC with expansion setting. Given that the interim sample size is driven by the initial PoC study, the interim sample size is generally fixed, however, through the sensitivity analysis, where the sample size in the control arm at interim varied from 20 up to 50, while the sample size in the experimental arm remained fixed at 40 patients, it is shown that power is maximised when the amount of control information at interim is at least comparable to that in the experimental arm. This finding highlights the potential value of borrowing from external control data at interim to increase the available information when the sample size in the control arm is small.

The impact of the biomarker distribution was also investigated, demonstrating that it is easier to identify a predictive effect when the biomarker distribution spreads patients across the entire biomarker range. This maximises variability and thus increases power. Consequently, a uniform biomarker distribution provides higher power compared to a Gamma distribution where biomarker values are concentrated within a narrower region, reducing the information available for assessing the predictive effect. However, uniformly distributed biomarkers are unlikely to occur in practice. In real clinical settings, biomarkers are likely to be skewed, with relatively few patients exhibiting extreme biomarker values. This highlights the importance of exploratory analysis of existing trial data or real‐world evidence to assess the plausible biomarker distributions prior to conducting simulation studies and planning the trial. Such a data‐driven exploration can inform the choice of biomarker distribution and parameter settings within the simulations, ensuring the simulation studies reflect realistic trial scenarios.

The proposed framework does have limitations. For instance, it does not address the difficulty in detecting a predictive effect in the presence of a prognostic effect. This can be partly attributed to the increase in standard error of the interaction coefficient and its shifted correlation to the prognostic term as the strength of the prognostic effect increases (see Section 3.4.4 of the Supporting Information for further details), reducing the models ability to reliably estimate the predictive effect. Ideally, as noted by Serra et al. [6], the prognostic effect of a biomarker should be carefully assessed prior to proposing and evaluating an expansion to detect a predictive effect. While certain aspects of the design can be modified to mitigate the substantially reduced ability to detect a predictive effect in the presence of a prognostic effect (such as increasing the total sample size at interim), overall power remains below 75% in the simulation scenarios with strong prognostic signals. These findings suggest an important practical implication: even with the implementation of adaptive design modifications, the ability to reliably detect predictive effects can be severely limited by dominant prognostic effects. Consequently, the biomarker‐driven expansion phase may only be justified in settings where the prognostic effect is not dominant, and where there is a reasonable expectation that a predictive signal can be distinguished from the prognostic effect. Further research into approaches for detecting predictive effects in the presence of substantial prognostic effects would be a valuable.

Another limitation is that only binary endpoints were explored in this work. However, the proposed adaptive framework is endpoint‐agnostic and not restricted to binary outcomes, as the interim decision‐making process and expansion cohort mechanism are not method‐specific in terms of assessing the biomarker's predictive effect. In fact, the AKSA method can by replaced with an alternative approach better suited to the endpoint of interest, such as an interaction test for continuous outcomes. Nevertheless, binary endpoints remain the most common in early‐phase oncology studies, directly reflecting our motivating trial setting. Furthermore, the framework was developed to build upon the AKSA method, which is currently only designed for binary outcomes. While continuous or time‐to‐event endpoints are inherently more information‐rich and may improve the ability to detect a biomarker's predictive effect, this should be formally evaluated through thorough simulation studies for the endpoint of interest.

Additionally, a ‘naive’ approach was implemented to define the threshold for segregating the BMK+ from the BMK‐ population. Specifically, this threshold was selected as the intersection point between the experimental and control logistic curves. Alternative approaches are available, including the method proposed by Liu [30]—which selects the cut‐off point that maximises the product of sensitivity and specificity—or the maximum Chi‐squared statistics [31], which maximises the association between the dichotomised biomarker and outcome. Future work could investigate the impact of alternative threshold‐selection methods on the operating characteristics of the proposed design.

Also, the decision to implement an expansion cohort is unlikely to be based solely on evidence regarding the predictive effect of the biomarker. In practice, the decision will likely be influenced by the primary efficacy analysis at the interim stage (e.g., at the originally specified samples size of 40 in the experimental arm and 20 in the control arm). For example, if the interim data already demonstrates sufficient evidence of overall efficacy, triggering an expansion phase to identify a predictive biomarker may be deemed less urgent since the trial success does not depend on it. However, this does not necessarily diminish the value of assessing the predictive effect of the biomarker. Even where overall efficacy has been established, identifying a predictive biomarker may still add substantial value by supporting a targeted development strategy and differentiation versus competing treatments. Conversely, if overall efficacy is inconclusive at the interim stage, detecting a predictive effect becomes more vital as it may represent the predominant approach for demonstrating treatment benefit. More generally, expansion cohorts are typically informed by multiple objectives including efficacy, safety, dose optimisation and biomarker evaluation. The proposed framework provides a quantitative, evidence‐based approach for the biomarker component of the broader decision‐making process. Future work could build on this framework by explicitly integrating the primary efficacy analysis into the expansion decision rules. The impact of joint decision criteria can be evaluated through simulation studies, with adjustments of the critical values to ensure the trial has appropriate operating characteristics.

Overall, the proposed framework provides a practical approach for assessing the predictive effect of a biomarker in small early‐phase trials, with justification of sample sizes, allocation ratios and decision criteria. Future work should focus on extending the framework beyond the current setting, considering alternative endpoints and improving the detection of predictive effects in the presence of strong prognostic effects.

## Funding

This work was supported by the National Institute for Health and Care Research (Grant No. NIHR300576) and the Medical Research Council (Grant No. MC UU 00040/03).

## Conflicts of Interest

The authors declare no conflicts of interest.

## Supporting information




**Data S1.** Supporting Information.

## Data Availability

All simulations were conducted through R version 4.1.1 in R studio. No new data have been used in this publication. Simulations can be reproduced using the open accessible code available at https://github.com/LDaniells/IA_BiomarkerPredictiveEffect.
