# Supplementary material for: Adaptive Trial Designs for Assessing Predictiveness of a Biomarker in Early Phase Drug Development
Source: Stat Med. 2026 Sep 8;45(20-22):e70719. doi: 10.1002/sim.70719 (PMC13551143; doi:10.1002/sim.70719)
Supplement: Supplementary file 1 [file SIM-45-0-s001.pdf]

Supplementary Materials: Adaptive Trial Designs for Assessing Predictiveness of a Biomarker in Early Phase Drug Development

Libby Daniells[1], Julia Geronimi[3], Hugo Hadjur[2], Sandrine Guilleminot[3], and Pavel Mozgunov[1,2]

[1]MRC Biostatistics Unit, University of Cambridge, UK
[2]Department of Statistical Methodology, SARYGA, France
[3]Servier, Paris-Saclay, France

1 Simulation Scenarios

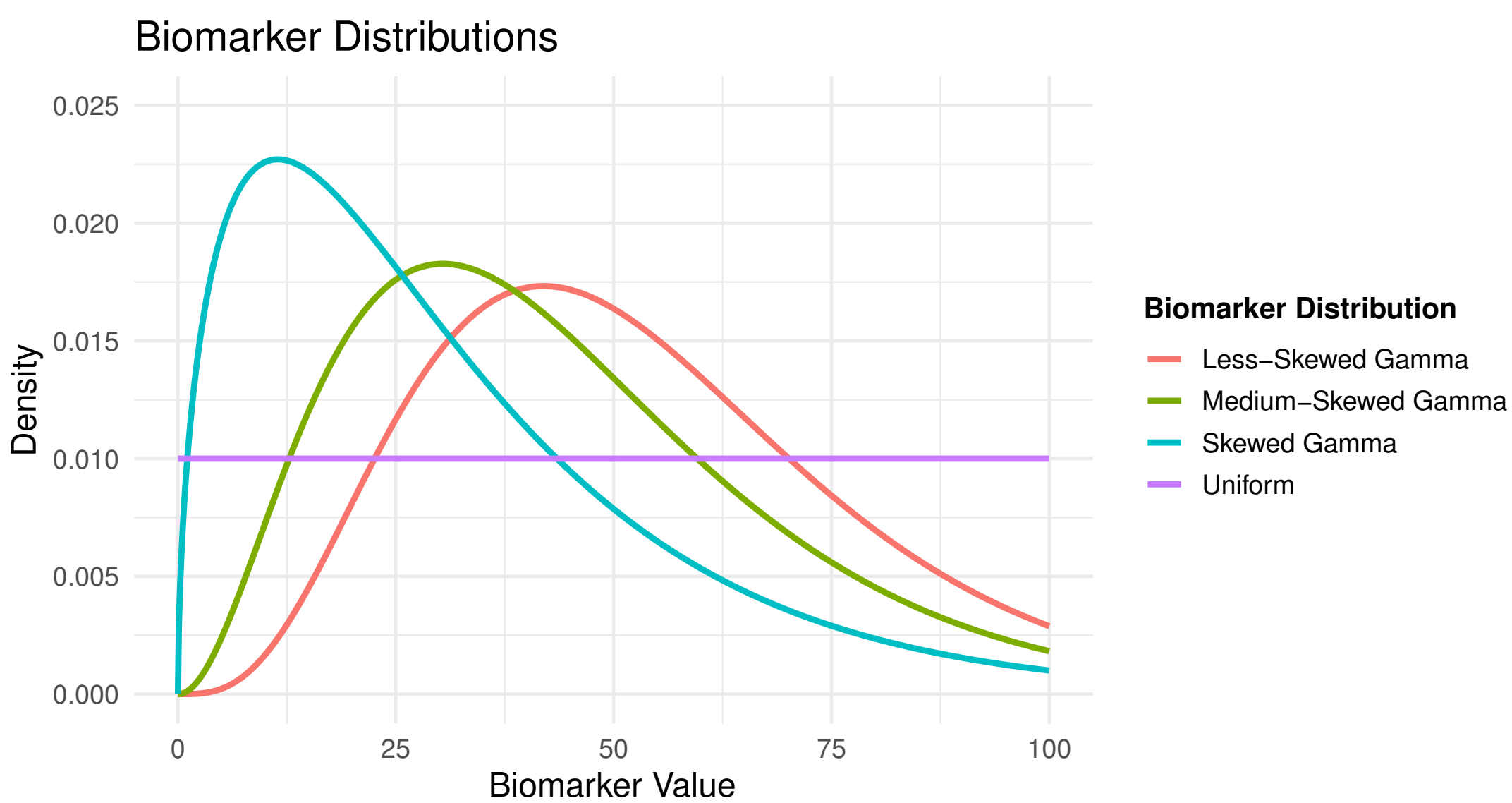

Figure 1: The Gamma and Uniform distributions considered for the continuous biomarker.

Table 1: Regression parameters $\mathbf{b} = \{b_0, b_1, b_2, b_3\}$ for each of the sixteen simulation scenarios implemented in the simulation study. These parameters are plugged into Equation (1) for data generation.

Scenario	b_0	b_1	b_2	b_3
NPNPT	-0.405	0.811	0.000	0.000
NPPT	-1.655	0.800	0.000	0.026
NPNPNT	-0.405	0.000	0.000	0.000
NPPNT	-1.655	0.000	0.000	0.026
HPNP_50	-0.405	-1.428	0.049	0.000
HPNP_40	-0.405	-0.868	0.035	0.000
MPNP_30	-0.405	-0.450	0.026	0.000
LPNP_20	-0.405	-0.020	0.017	0.000
HPMP_50	-0.405	-1.428	0.049	0.010
HPMP_40	-0.405	-0.868	0.035	0.007
MPMP_30	-0.405	-0.450	0.026	0.005
LPMP_20	-0.405	-0.020	0.017	0.003
HPHP_50	-0.405	-1.428	0.049	0.025
HPHP_40	-0.405	-0.868	0.035	0.018
MPHP_30	-0.405	-0.450	0.026	0.013
LPHP_20	-0.405	-0.020	0.017	0.009

2 Decision Bounds

Table 2: Calibrated decision bounds η_0 and $\eta_1 = \eta_2$ under the 2-Bound configuration for different post-interim sample sizes and calibration rules.

Decision Bound	Calibration Rule	Post-Interim SS 10	15	20	25	30
η_0	80%	0.707	0.703	0.711	0.702	0.711
	75%	0.674	0.667	0.676	0.667	0.677
	70%	0.642	0.633	0.644	0.633	0.647
$\eta_1 = \eta_2$	80%	0.758	0.749	0.756	0.747	0.755
	75%	0.764	0.758	0.762	0.754	0.762
	70%	0.766	0.761	0.765	0.762	0.766

Table 3: Calibrated decision bounds η_0, η_1 and η_2 under the 3-Bound configuration for different post-interim sample sizes and calibration rules.

Decision Bound	Calibration Rule	Post-Interim SS 10	15	20	25	30
η_0	80%	0.704	0.708	0.703	0.709	0.708
	75%	0.671	0.673	0.672	0.678	0.676
	70%	0.643	0.638	0.639	0.645	0.640
η_1	80%	0.794	0.791	0.789	0.793	0.789
	75%	0.794	0.791	0.789	0.793	0.789
	70%	0.794	0.791	0.789	0.793	0.789
η_2	80%	0.694	0.668	0.664	0.653	0.650
	75%	0.725	0.717	0.711	0.703	0.681
	70%	0.734	0.731	0.734	0.729	0.711

Table 4: Calibrated decision bounds η_0, η_1 and η_2 under the total balanced sample size configuration for different post-interim sample sizes and 70% calibration rule.

Decision Bound	Total Sample Size 80	90	100	110	120
η_0	0.647	0.641	0.646	0.646	0.641
η_1	0.794	0.794	0.794	0.792	0.789
η_2	0.734	0.731	0.726	0.731	0.715

Table 5: Calibrated decision bounds η_0, η_1 and η_2 under different interim sample sizes with expansion sample sizes varied.

Decision Bound	$n_{0,1}$	Post-Interim SS 10	15	20	25	30
η_0	20	0.643	0.638	0.639	0.645	0.640
	30	0.643	0.647	0.632	0.639	0.644
	35	0.640	0.643	0.639	0.635	0.635
	40	0.638	0.632	0.634	0.643	0.643
	50	0.639	0.641	0.643	0.642	0.647
η_1	20	0.794	0.791	0.789	0.793	0.789
	30	0.794	0.791	0.790	0.795	0.793
	35	0.799	0.790	0.794	0.797	0.795
	40	0.793	0.792	0.794	0.797	0.791
	50	0.792	0.794	0.797	0.796	0.798
η_2	20	0.734	0.731	0.734	0.729	0.711
	30	0.733	0.737	0.733	0.733	0.725
	35	0.743	0.739	0.734	0.734	0.726
	40	0.737	0.730	0.737	0.738	0.741
	50	0.740	0.736	0.742	0.735	0.731

Table 6: Calibrated decision bounds η_0, η_1 and η_2 under different biomarker distributions with expansion sample sizes varied.

Decision Bound	Biomarker Distribution	Post-Interim SS 10	15	20	25	30
η_0	Less-Skewed Gamma	0.643	0.638	0.639	0.645	0.640
	Medium-Skewed Gamma	0.642	0.647	0.639	0.640	0.635
	Skewed Gamma	0.634	0.634	0.633	0.632	0.645
	Uniform	0.619	0.621	0.622	0.630	0.623
η_1	Less-Skewed Gamma	0.794	0.791	0.789	0.793	0.789
	Medium-Skewed Gamma	0.791	0.789	0.788	0.790	0.792
	Skewed Gamma	0.777	0.777	0.776	0.779	0.776
	Uniform	0.773	0.777	0.773	0.774	0.780
η_2	Less-Skewed Gamma	0.734	0.731	0.734	0.729	0.711
	Medium-Skewed Gamma	0.730	0.726	0.721	0.715	0.709
	Skewed Gamma	0.728	0.721	0.722	0.709	0.714
	Uniform	0.713	0.709	0.695	0.714	0.698

3 Simulation Results

3.1 Equal Allocation in Expansion Phase

3.1.1 3-Bound Configuration

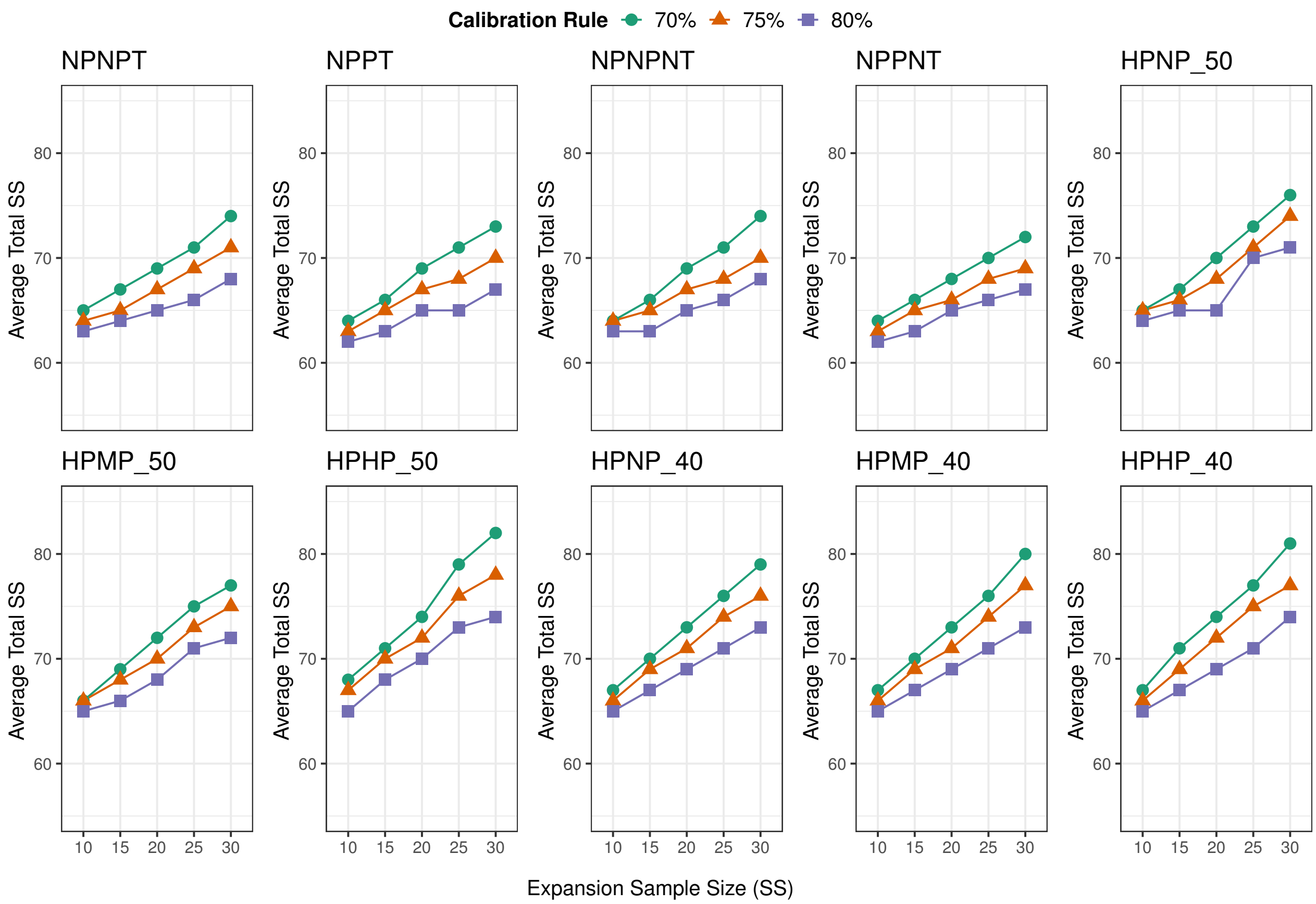

Figure 2: Average sample size under the 3-Bound configuration for the null and high predictive scenarios. Results are provided for three different calibration rules: 70%, 75% and 80% and different expansion sample sizes: $n_{0,2} = n_{1,2} = 10, 15, 20, 25, 30$.

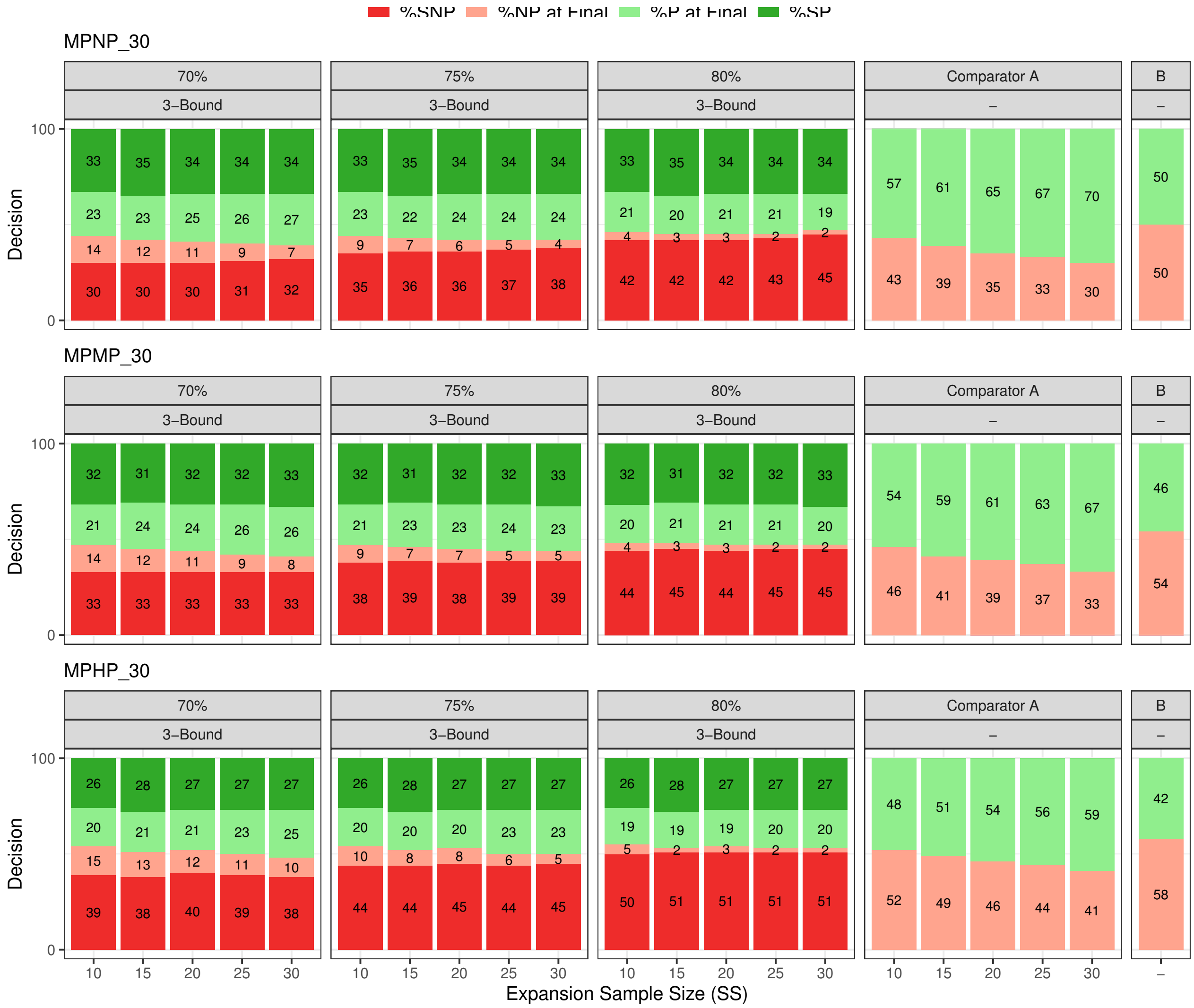

Figure 3: Percentage of simulations under the alternative scenarios with 30% predictive effect (5000 replications, 3-Bound configuration) that resulted in each decision category: predictive (%SP, dark green) or non-predictive (%SNP, dark red) at interim, or predictive (%P at Final, light green) or non-predictive (%NP at Final, light red) at final analysis. Results are by expansion sample size and calibration rule (70%, 75% and 80%). Comparator A is the design with no-interim but total sample size matched to the interim design. Comparator B is the no-interim design with 40:20 allocation.

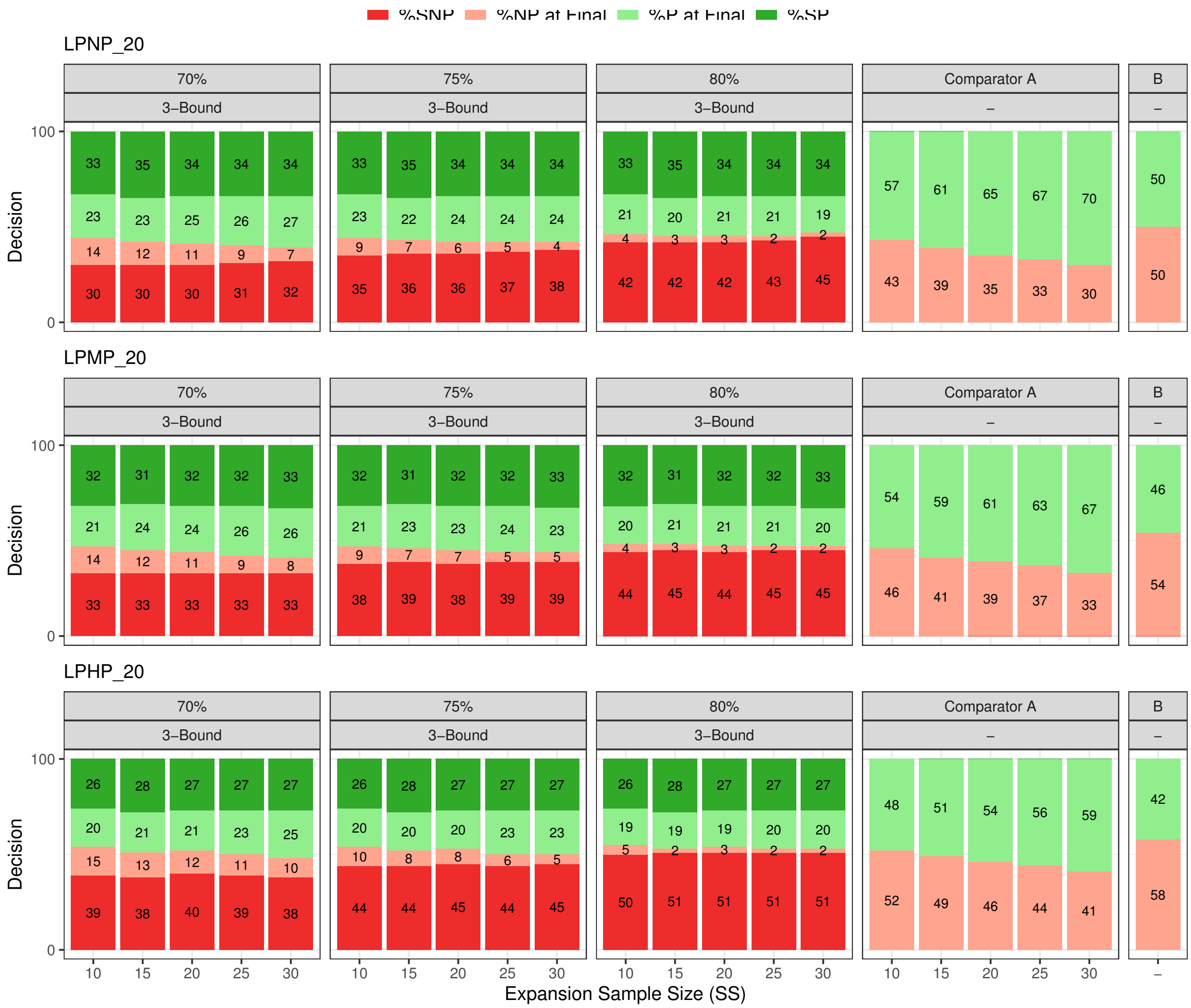

Figure 4: Percentage of simulations under the alternative scenarios with 20% predictive effect (5000 replications, 3-Bound configuration) that resulted in each decision category: predictive (%SP, dark green) or non-predictive (%SNP, dark red) at interim, or predictive (%P at Final, light green) or non-predictive (%NP at Final, light red) at final analysis. Results are by expansion sample size and calibration rule (70%, 75% and 80%). Comparator A is the design with no-interim but total sample size matched to the interim design. Comparator B is the no-interim design with 40:20 allocation.

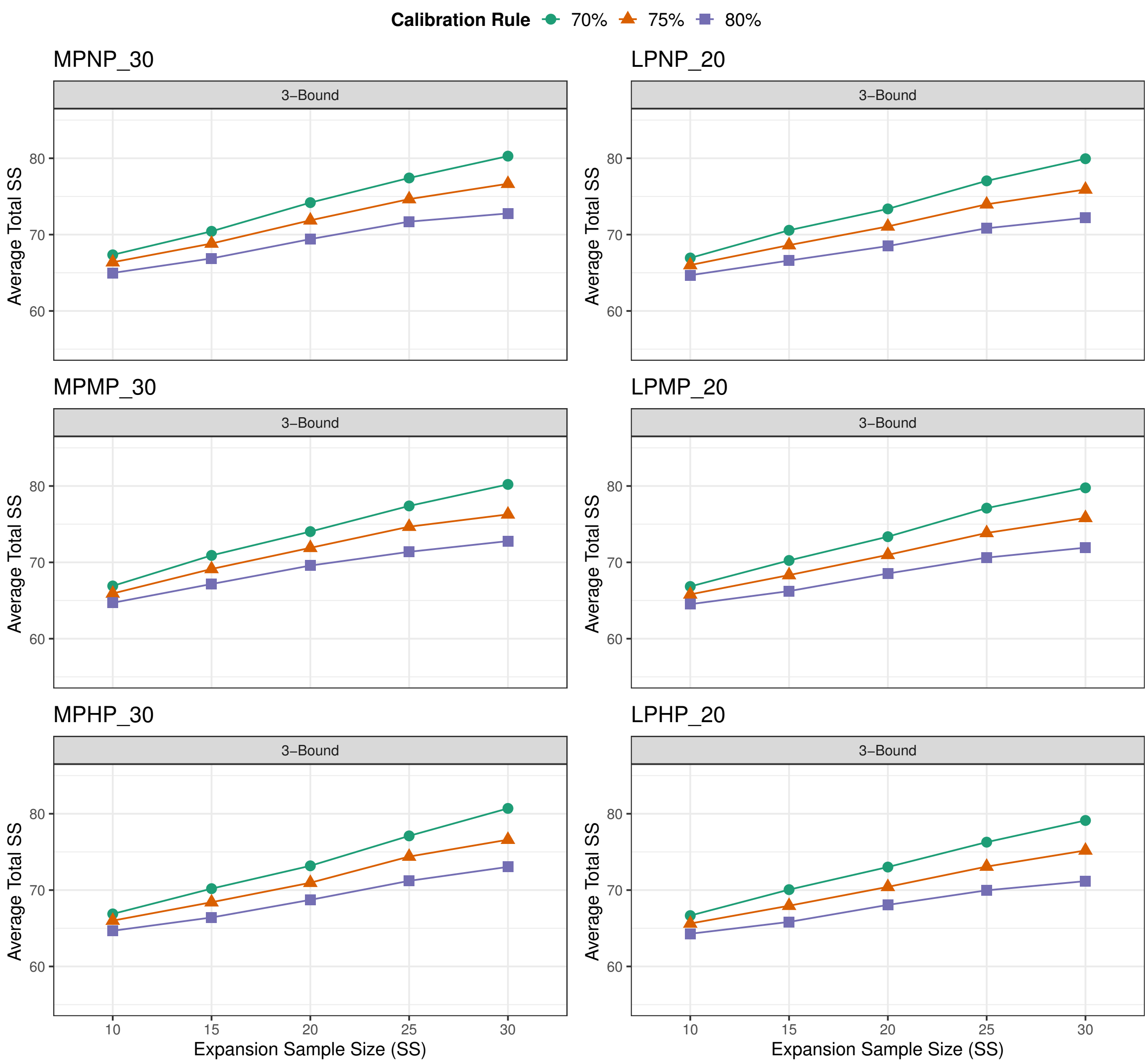

Figure 5: Average sample size under the 3-Bound configuration for the medium and low predictive scenarios. Results are provided for three different calibration rules: 70%, 75% and 80% and different expansion sample sizes: $n_{0,2} = n_{1,2} = 10, 15, 20, 25, 30$.

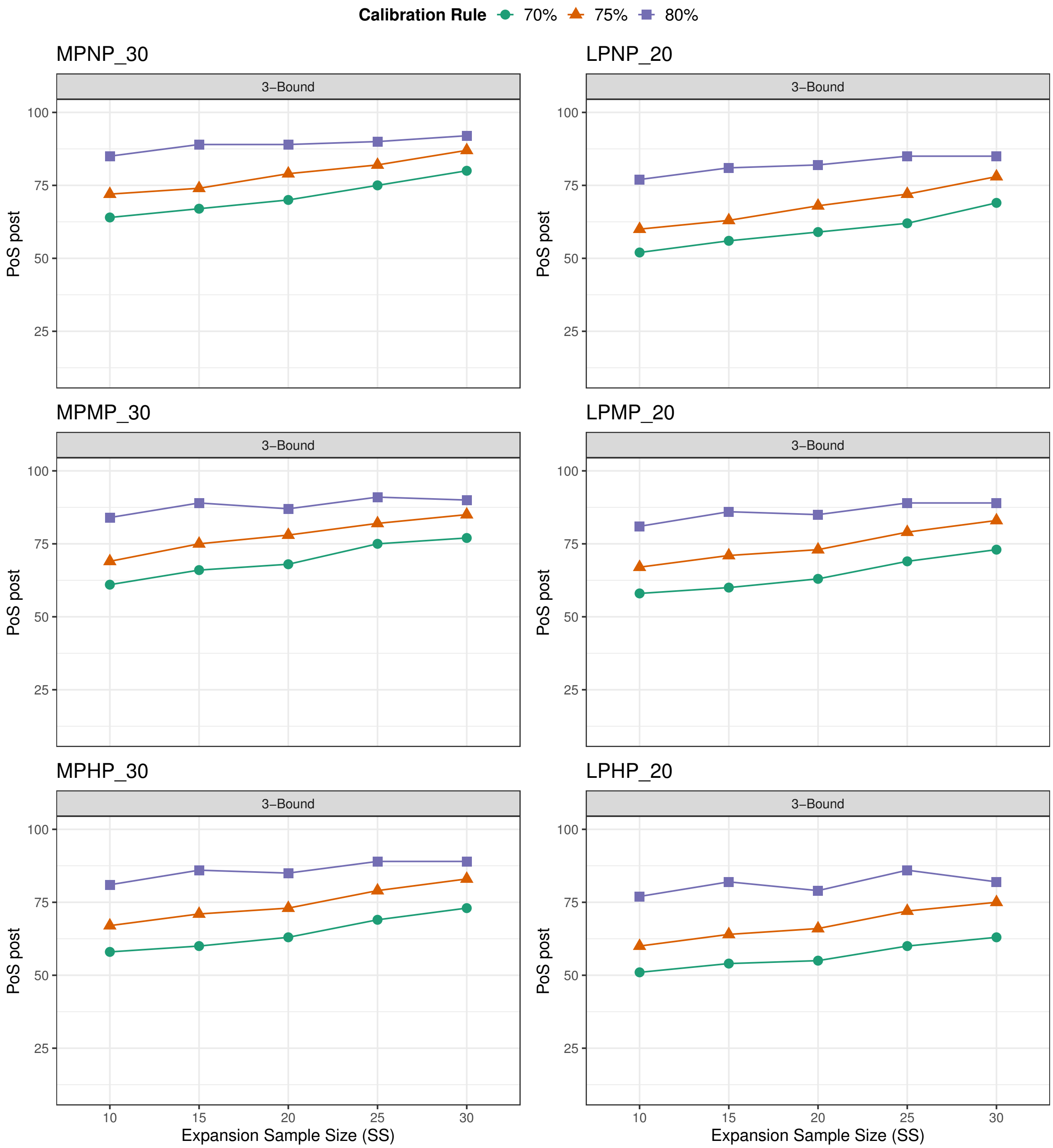

Figure 6: PoS under the 3-Bound configuration for the medium and low predictive scenarios. Results are provided for three different calibration rules: 70%, 75% and 80% and different expansion sample sizes: $n_{0,2} = n_{1,2} = 10, 15, 20, 25, 30$.

Table 7: Simulation results for the 3-bound design with equal allocation in the expansion phase. Results implement a 70% calibration rule and cover the null and high predictive scenarios.

Scenario	Post-Interim SS	T1E/Power	% SNP	% SP	Average N	PoS post
NPNPT	10	15	70	7	65	35
	15	15	70	7	67	34
	20	15	70	7	69	34
	25	15	70	7	71	33
	30	15	70	7	74	33
NPPT	10	14	71	7	64	33
	15	14	72	7	66	34
	20	14	72	7	69	31
	25	14	72	7	71	33
	30	15	71	7	73	37
NPNPNT	10	15	71	7	64	37
	15	14	72	7	66	34
	20	14	70	7	69	31
	25	14	72	7	71	34
	30	15	70	7	74	35
NPPNT	10	14	72	7	64	34
	15	15	71	7	66	34
	20	14	73	7	68	32
	25	14	72	7	70	34
	30	15	72	7	72	37
HPNP_50	10	83	12	61	65	81
	15	85	12	64	67	85
	20	85	12	64	70	88
	25	86	12	62	73	92
	30	87	12	62	76	96
HPMP_50	10	76	16	53	66	74
	15	78	17	53	69	84
	20	78	17	54	72	82
	25	80	17	54	75	88
	30	82	16	55	77	92
HPHP_50	10	58	29	33	68	67
	15	62	27	35	71	72
	20	63	28	36	74	75
	25	64	28	35	79	79
	30	65	28	36	82	84
HPNP_40	10	69	21	45	67	72
	15	70	22	46	70	76
	20	72	21	47	73	80
	25	72	23	46	76	85
	30	75	21	47	79	89
HPMP_40	10	63	26	39	67	69
	15	66	25	41	70	74
	20	68	24	43	73	75
	25	68	26	41	76	82
	30	70	25	41	80	87
HPHP_40	10	53	33	30	67	64
	15	56	32	32	71	68
	20	56	32	32	74	66
	25	56	34	31	77	72
	30	59	34	31	81	80

Table 8: Simulation results for the 3-bound design with equal allocation in the expansion phase. Results implement a 75% calibration rule and cover the null and high predictive scenarios.

Scenario	Post-Interim SS	T1E/Power	% SNP	% SP	Average N	% PoS post
NPNPT	10	14	75	7	64	43
	15	15	75	7	65	43
	20	15	75	7	67	43
	25	15	75	7	69	43
	30	15	75	7	71	44
NPPT	10	15	76	7	63	43
	15	14	77	7	65	42
	20	14	77	7	67	42
	25	14	77	7	68	45
	30	15	77	7	70	50
NPNPNT	10	15	75	7	64	44
	15	14	77	7	65	44
	20	15	75	7	67	41
	25	14	77	7	68	44
	30	15	76	7	70	46
NPPNT	10	14	76	7	63	41
	15	15	76	7	65	43
	20	14	77	7	66	42
	25	14	77	7	68	44
	30	15	77	7	69	49
HPNP_50	10	82	14	61	65	86
	15	83	15	64	66	90
	20	84	15	64	68	93
	25	84	15	62	71	95
	30	84	15	62	74	98
HPMP_50	10	75	18	53	66	80
	15	77	21	53	68	89
	20	77	21	54	70	89
	25	77	21	54	73	93
	30	79	20	55	75	95
HPHP_50	10	58	33	33	67	75
	15	60	33	35	70	80
	20	62	33	36	72	84
	25	62	34	35	76	87
	30	62	34	36	78	90
HPNP_40	10	68	25	45	66	77
	15	69	26	46	69	82
	20	70	26	47	71	87
	25	70	27	46	74	90
	30	72	26	47	76	93
HPMP_40	10	62	30	39	66	75
	15	64	30	41	69	81
	20	66	29	43	71	83
	25	67	30	41	74	89
	30	67	31	41	77	92
HPHP_40	10	53	38	30	66	71
	15	55	38	32	69	75
	20	54	38	32	72	75
	25	55	40	31	75	81
	30	57	40	31	77	88

Table 9: Simulation results for the 3-bound design with equal allocation in the expansion phase. Results implement a 80% calibration rule and cover the null and high predictive scenarios.

Scenario	Post-Interim SS	T1E/Power	% SNP	% SP	Average N	% PoS post
NPNPT	10	15	81	7	63	62
	15	15	80	7	64	61
	20	15	80	7	65	60
	25	15	80	7	66	60
	30	15	80	7	68	54
NPPT	10	15	80	7	62	60
	15	14	81	7	63	63
	20	14	81	7	65	61
	25	14	81	7	65	61
	30	14	82	7	67	61
NPNPNT	10	15	80	7	63	61
	15	14	82	7	63	62
	20	15	80	7	65	59
	25	14	81	7	66	62
	30	14	80	7	68	56
NPPNT	10	14	81	7	62	60
	15	14	81	7	63	64
	20	14	81	7	65	59
	25	14	81	7	66	59
	30	14	81	7	67	59
HPNP_50	10	80	18	61	64	93
	15	81	19	64	65	97
	20	82	18	64	65	97
	25	81	19	62	70	98
	30	81	19	62	71	98
HPMP_50	10	74	24	53	65	89
	15	73	26	53	66	95
	20	74	25	54	68	95
	25	74	25	54	71	98
	30	75	25	55	72	97
HPHP_50	10	57	39	33	65	88
	15	58	40	35	68	92
	20	59	38	36	70	92
	25	58	40	35	73	95
	30	58	41	36	74	95
HPNP_40	10	67	30	45	65	89
	15	66	32	46	67	93
	20	67	31	47	69	94
	25	67	33	46	71	96
	30	67	32	47	73	96
HPMP_40	10	60	37	39	65	88
	15	61	37	41	67	91
	20	64	35	43	69	92
	25	63	36	41	71	95
	30	62	37	41	73	95
HPHP_40	10	51	45	30	65	85
	15	53	45	32	67	90
	20	53	44	32	69	88
	25	52	46	31	71	90
	30	52	46	31	74	92

3.1.2 2-Bound Configuration

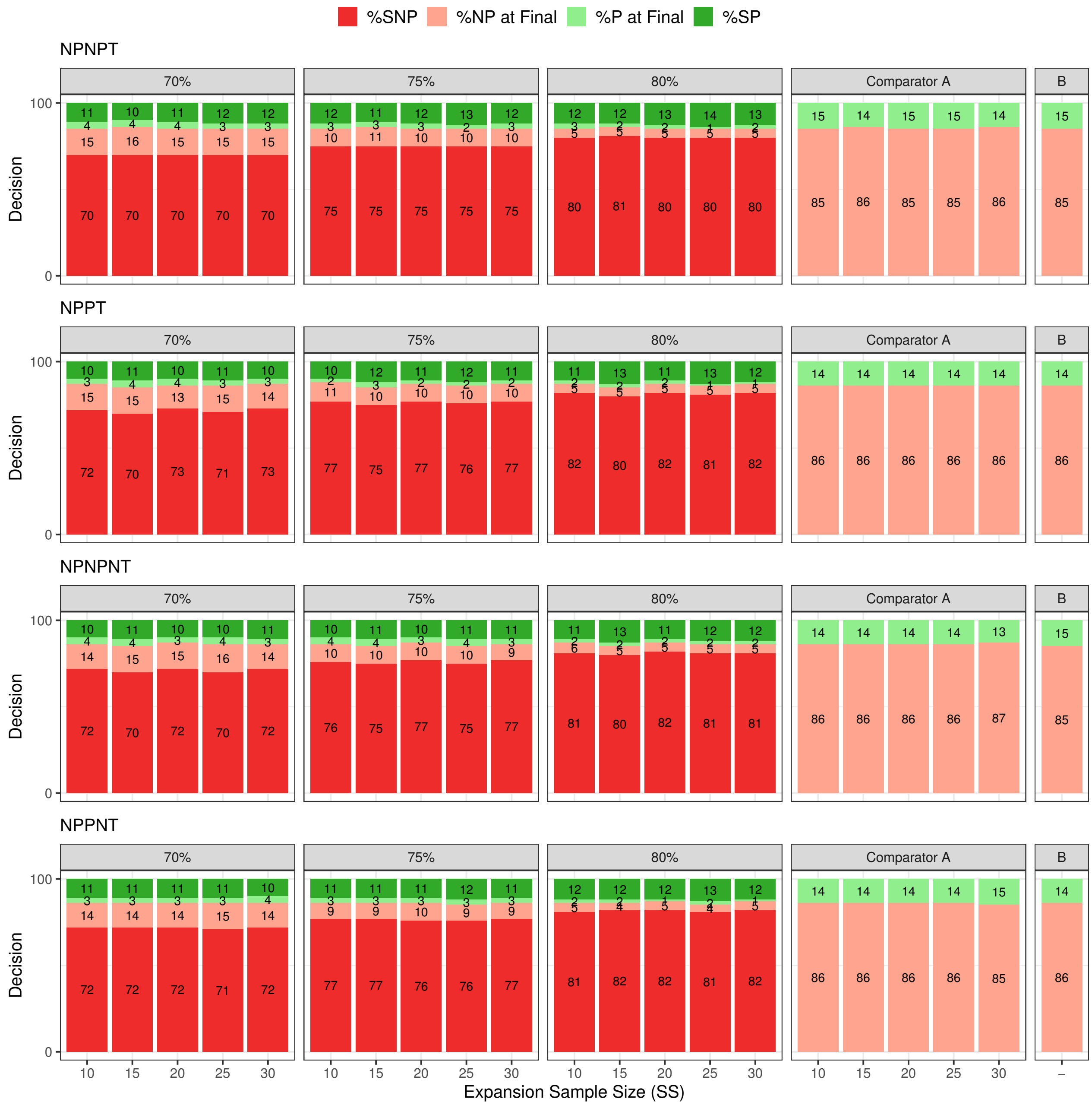

Figure 7: Percentage of simulations under the null scenarios (5000 replications, 2-Bound configuration) that resulted in each decision category: predictive (%SP, dark green) or non-predictive (%SNP, dark red) at interim, or predictive (%P at Final, light green) or non-predictive (%NP at Final, light red) at final analysis. Results are by expansion sample size and calibration rule (70%, 75% and 80%). Comparator A is the design with no-interim but total sample size matched to the interim design. Comparator B is the no-interim design with 40:20 allocation.

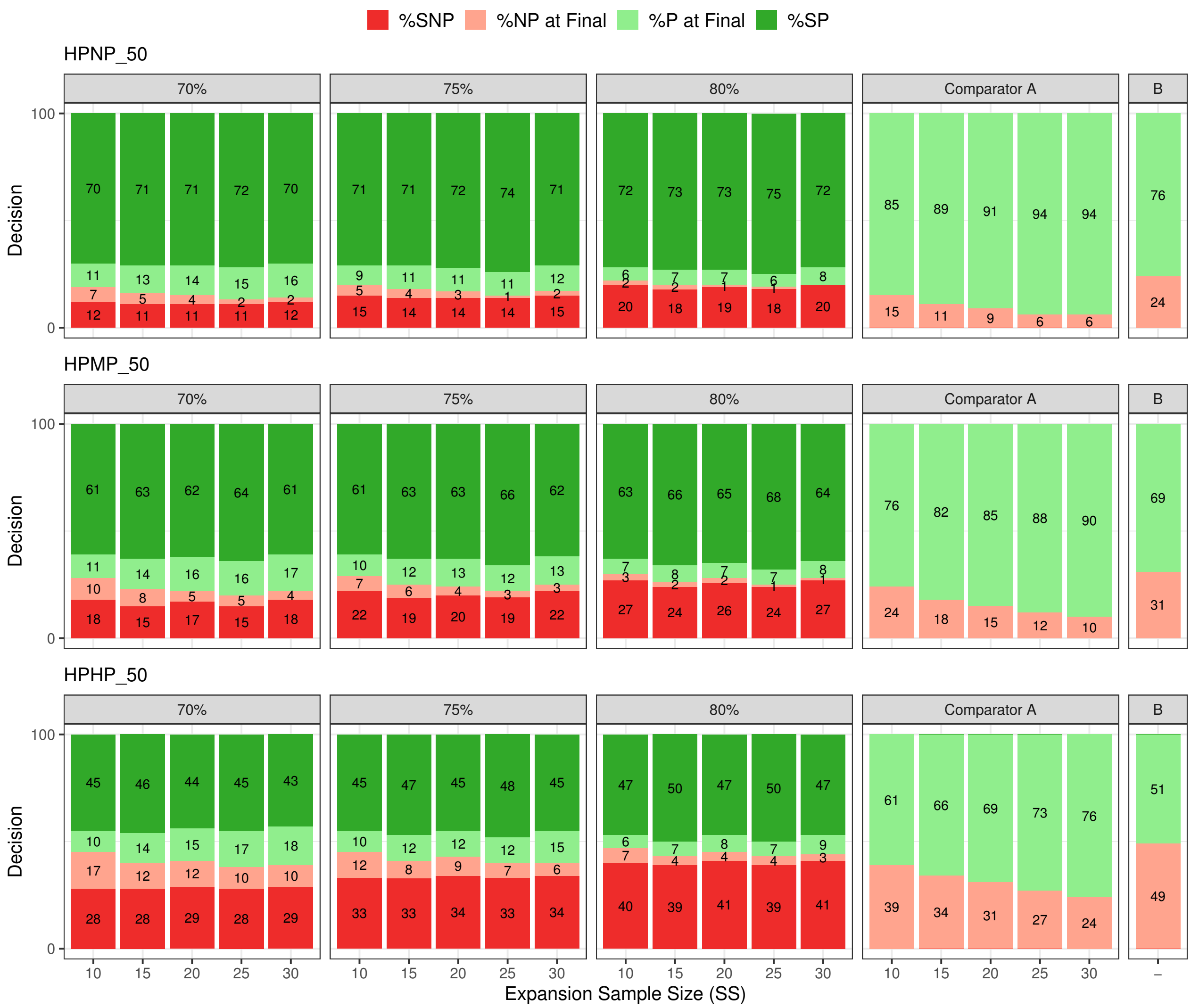

Figure 8: Percentage of simulations under the alternative scenarios with 50% predictive effect (5000 replications, 2-Bound configuration) that resulted in each decision category: predictive (%SP, dark green) or non-predictive (%SNP, dark red) at interim, or predictive (%P at Final, light green) or non-predictive (%NP at Final, light red) at final analysis. Results are by expansion sample size and calibration rule (70%, 75% and 80%). Comparator A is the design with no-interim but total sample size matched to the interim design. Comparator B is the no-interim design with 40:20 allocation.

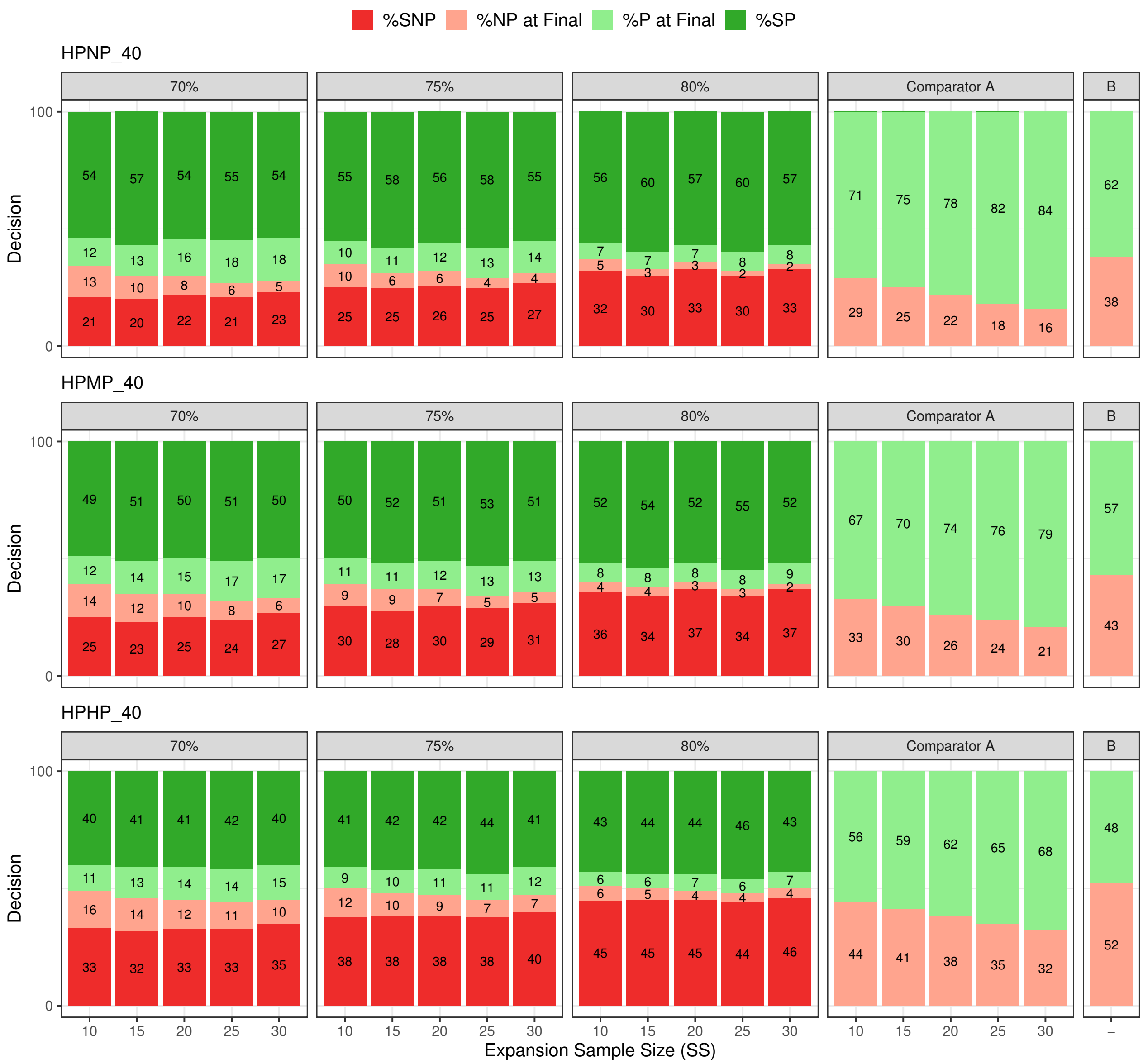

Figure 9: Percentage of simulations under the alternative scenarios with 40% predictive effect (5000 replications, 2-Bound configuration) that resulted in each decision category: predictive (%SP, dark green) or non-predictive (%SNP, dark red) at interim, or predictive (%P at Final, light green) or non-predictive (%NP at Final, light red) at final analysis. Results are by expansion sample size and calibration rule (70%, 75% and 80%). Comparator A is the design with no-interim but total sample size matched to the interim design. Comparator B is the no-interim design with 40:20 allocation.

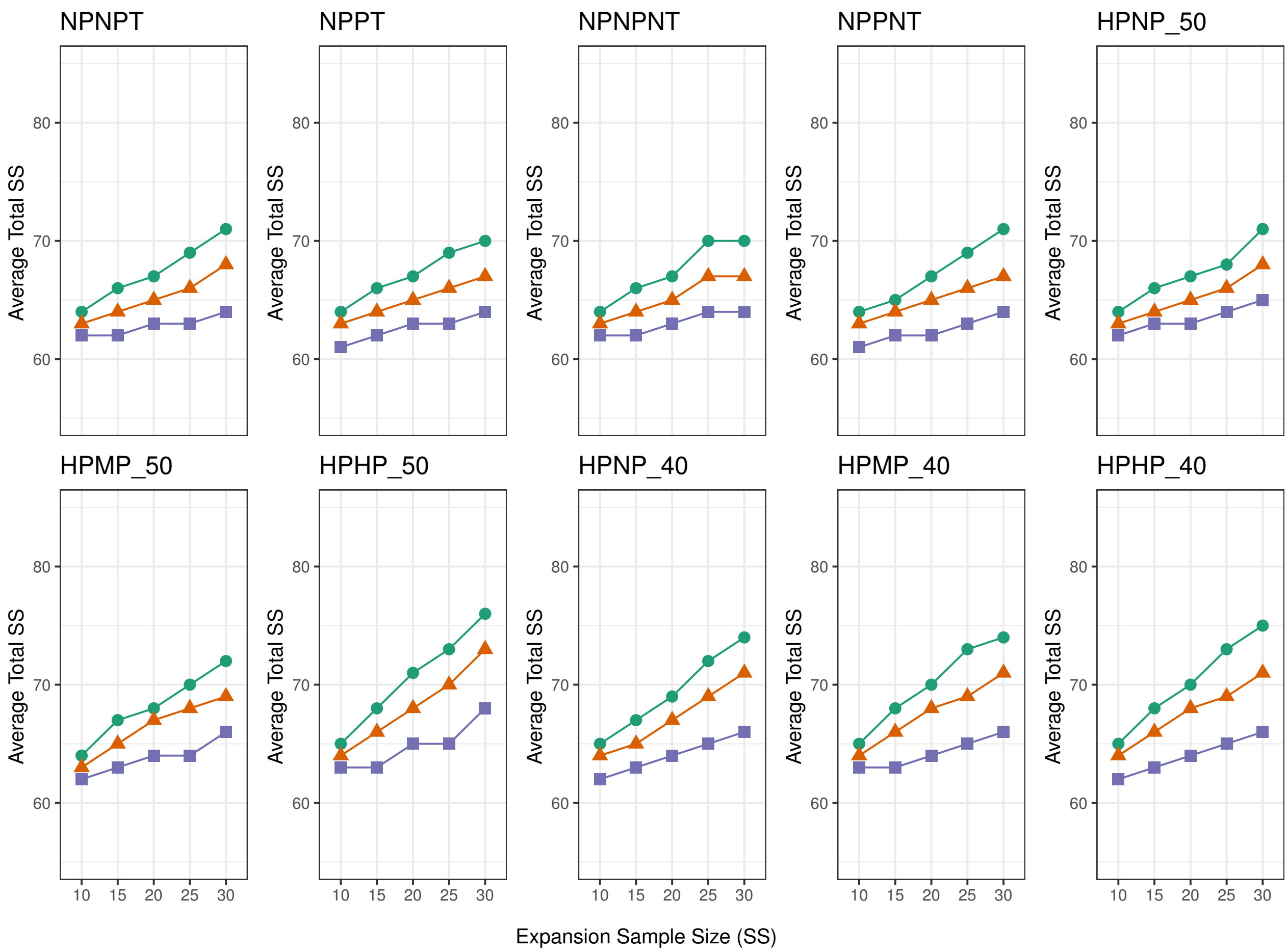

Figure 10: Average sample size under the 2-Bound configuration for the null and high predictive scenarios. Results are provided for three different calibration rules: 70%, 75% and 80% and different expansion sample sizes: $n_{0,2} = n_{1,2} = 10, 15, 20, 25, 30$.

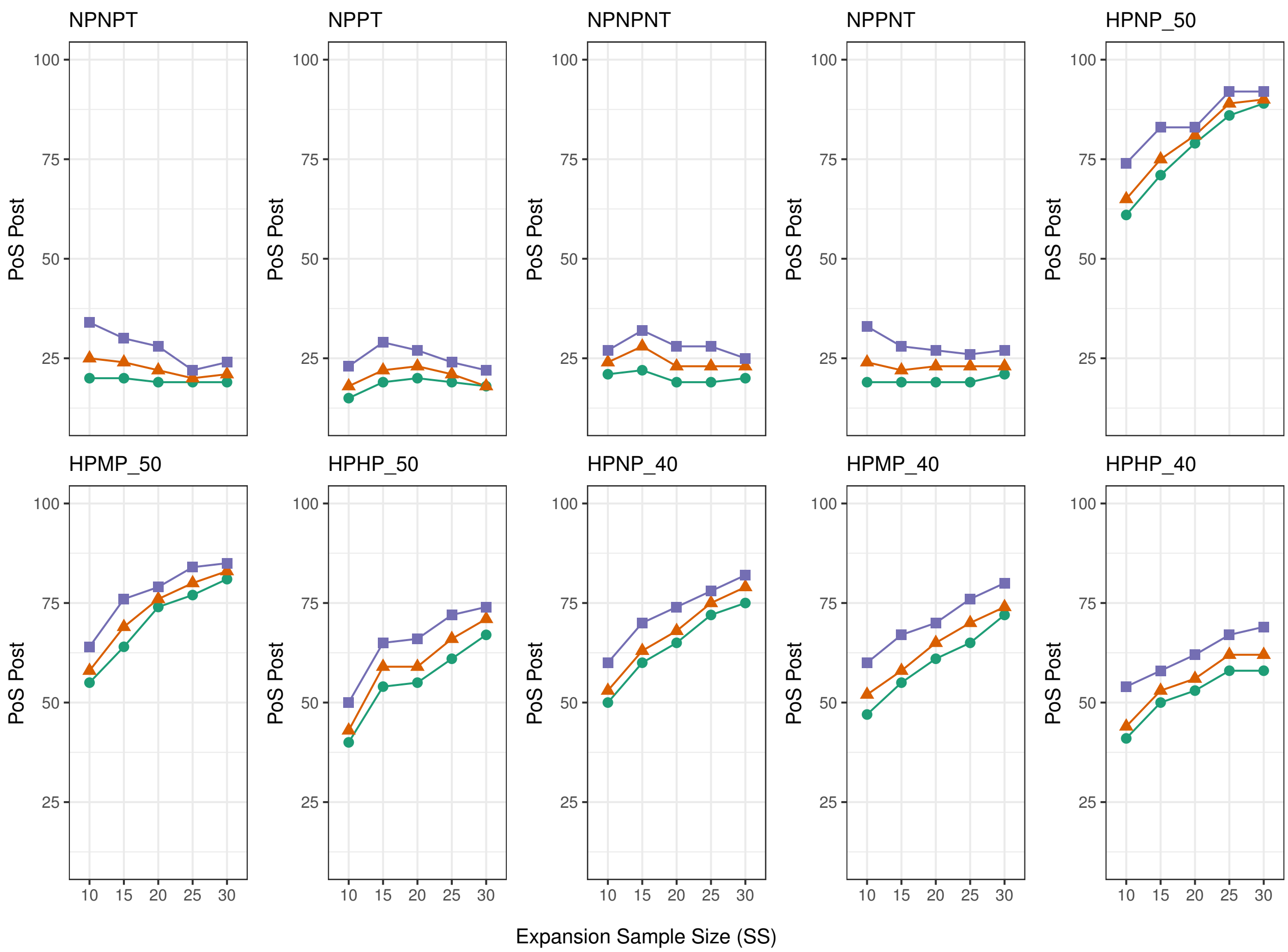

Figure 11: PoS post under the 2-Bound configuration for the null and high predictive scenarios. Results are provided for three different calibration rules: 70%, 75% and 80% and different expansion sample sizes: $n_{0,2} = n_{1,2} = 10, 15, 20, 25, 30$.

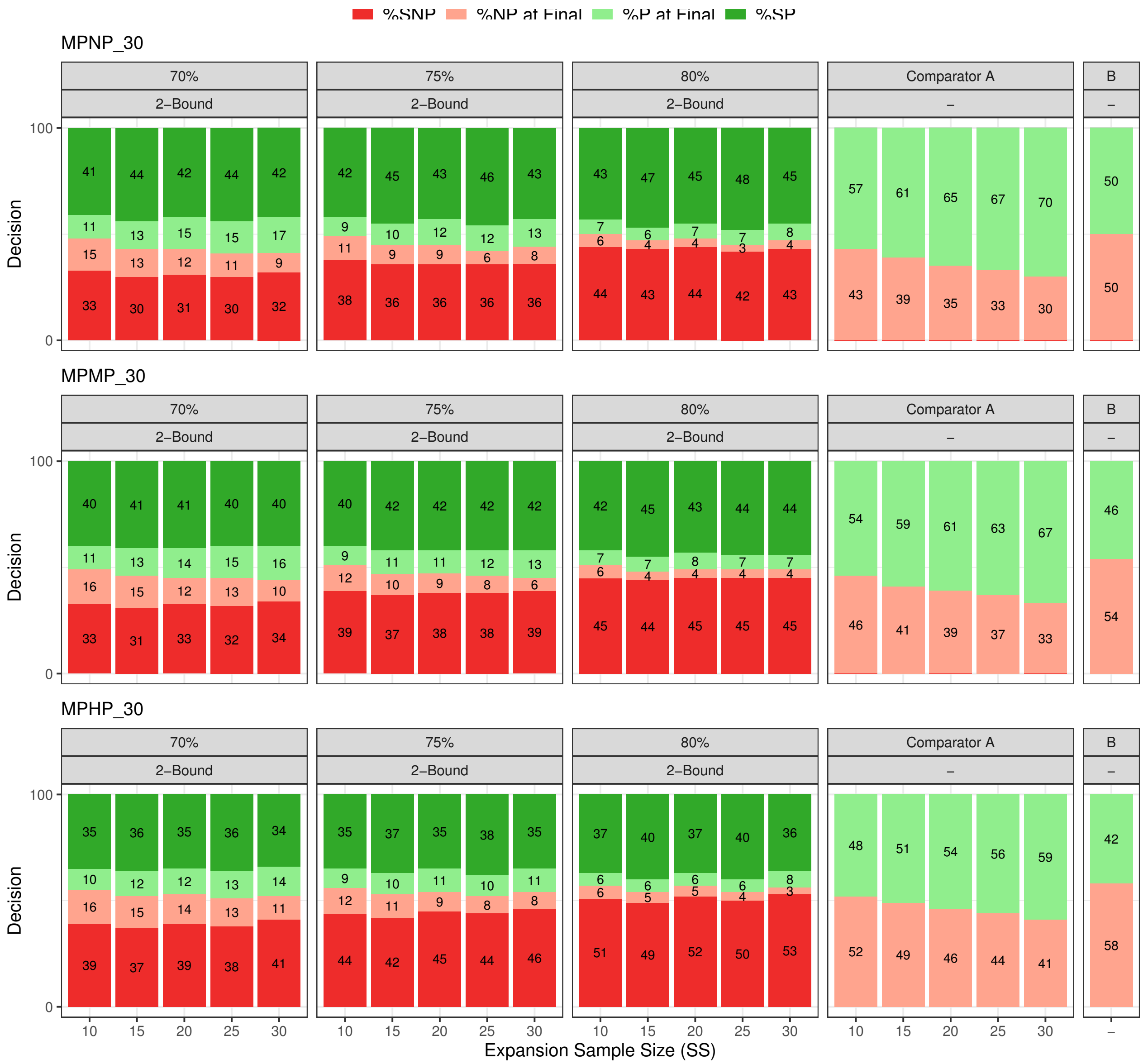

Figure 12: Percentage of simulations under the alternative scenarios with 30% predictive effect (5000 replications, 3-Bound configuration) that resulted in each decision category: predictive (%SP, dark green) or non-predictive (%SNP, dark red) at interim, or predictive (%P at Final, light green) or non-predictive (%NP at Final, light red) at final analysis. Results are by expansion sample size and calibration rule (70%, 75% and 80%). Comparator A is the design with no-interim but total sample size matched to the interim design. Comparator B is the no-interim design with 40:20 allocation.

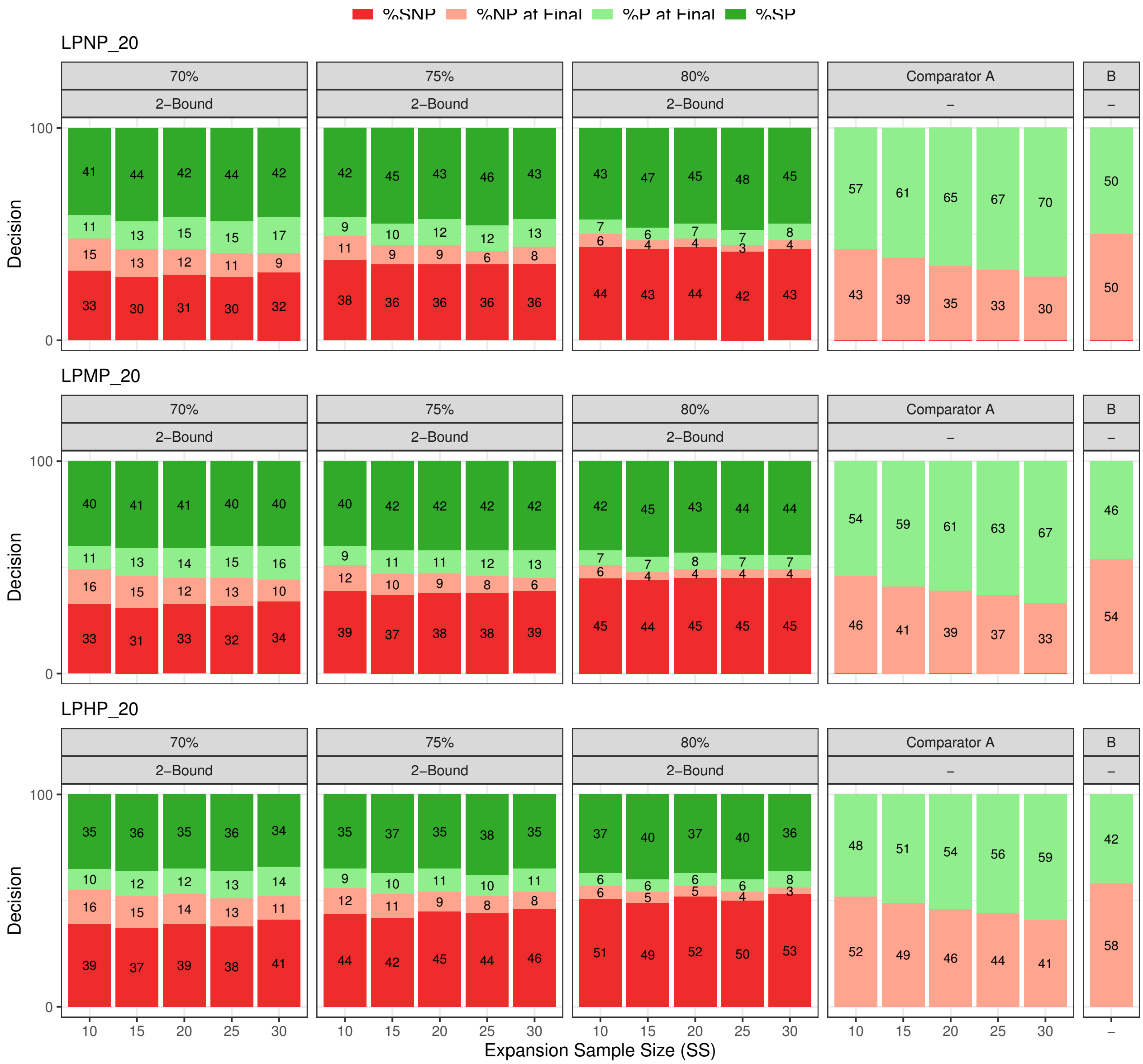

Figure 13: Percentage of simulations under the alternative scenarios with 20% predictive effect (5000 replications, 3-Bound configuration) that resulted in each decision category: predictive (%SP, dark green) or non-predictive (%SNP, dark red) at interim, or predictive (%P at Final, light green) or non-predictive (%NP at Final, light red) at final analysis. Results are by expansion sample size and calibration rule (70%, 75% and 80%). Comparator A is the design with no-interim but total sample size matched to the interim design. Comparator B is the no-interim design with 40:20 allocation.

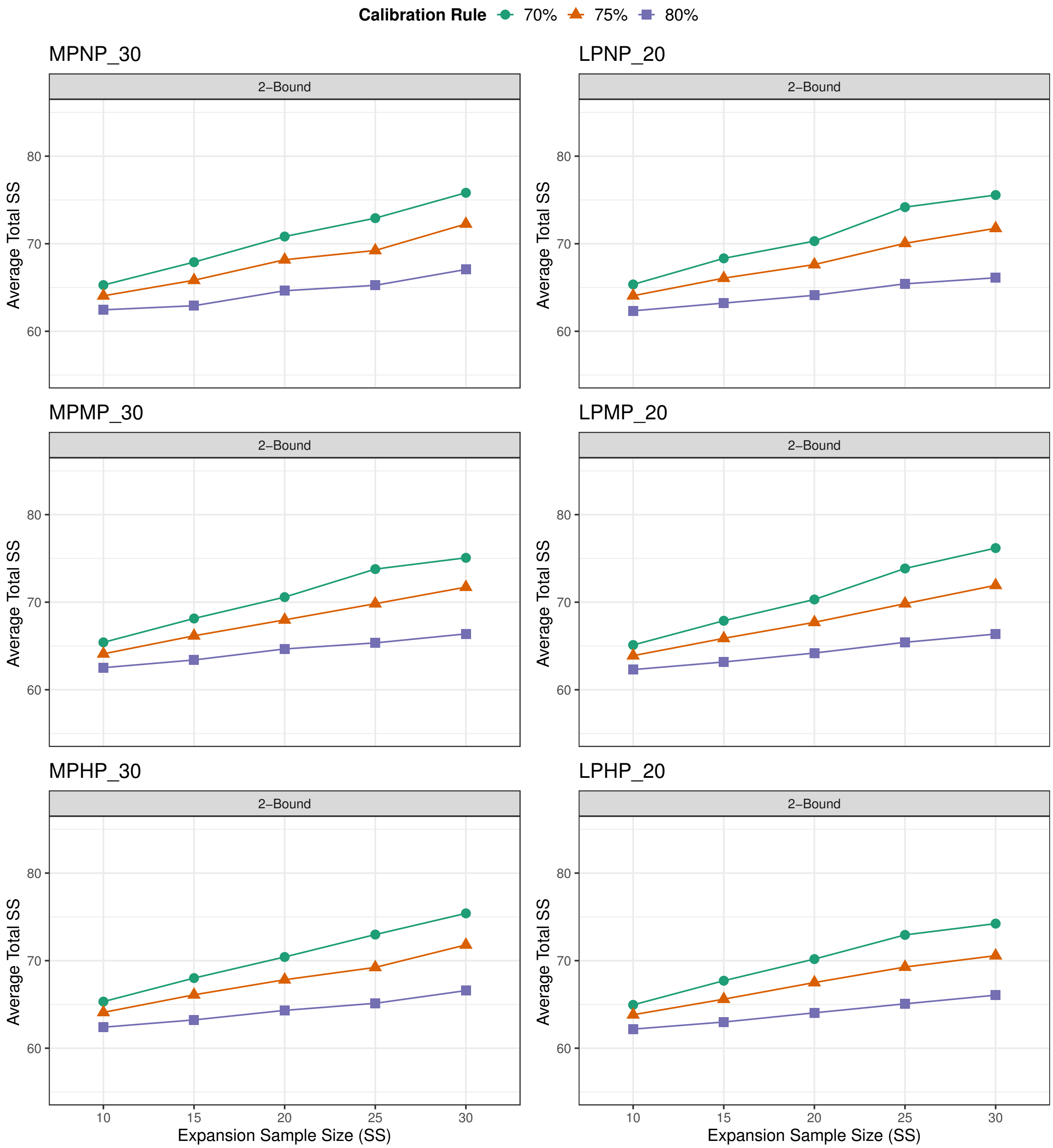

Figure 14: Average sample size under the 2-Bound configuration for the medium and low predictive scenarios. Results are provided for three different calibration rules: 70%, 75% and 80% and different expansion sample sizes: $n_{0,2} = n_{1,2} = 10, 15, 20, 25, 30$.

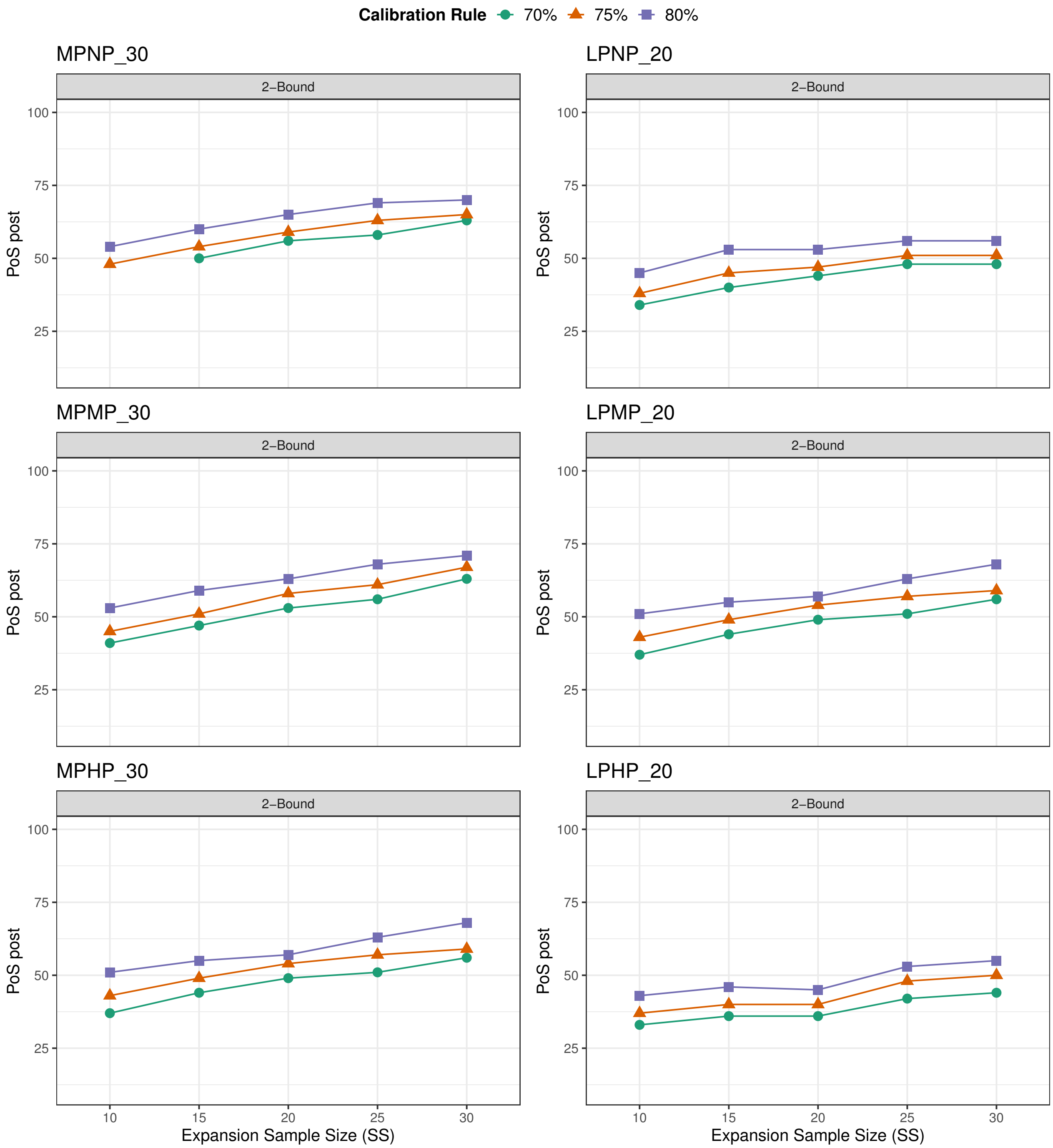

Figure 15: PoS post under the 2-Bound configuration for the medium and low predictive scenarios. Results are provided for three different calibration rules: 70%, 75% and 80% and different expansion sample sizes: $n_{0,2} = n_{1,2} = 10, 15, 20, 25, 30$.

Table 10: Simulation results for the 2-bound design with equal allocation in the expansion phase. Results implement a 70% calibration rule and cover the null and high predictive scenarios.

Scenario	Post-Interim SS	T1E/Power	% SNP	% SP	Average N	% PoS post
NPNPT	10	15	70	11	64	20
	15	14	70	10	66	20
	20	15	70	11	67	19
	25	15	70	12	69	19
	30	15	70	12	71	19
NPPT	10	13	72	10	64	15
	15	15	70	11	66	19
	20	14	73	10	67	20
	25	14	71	11	69	19
	30	13	73	10	70	18
NPNPNT	10	14	72	10	64	21
	15	15	70	11	66	22
	20	13	72	10	67	19
	25	14	70	10	70	19
	30	14	72	11	70	20
NPPNT	10	14	72	11	64	19
	15	14	72	11	65	19
	20	14	72	11	67	19
	25	14	71	11	69	19
	30	14	72	10	71	21
HPNP_50	10	81	12	70	64	61
	15	84	11	71	66	71
	20	85	11	71	67	79
	25	87	11	72	68	86
	30	86	12	70	71	89
HPMP_50	10	72	18	61	64	55
	15	77	15	63	67	64
	20	78	17	62	68	74
	25	80	15	64	70	77
	30	78	18	61	72	81
HPHP_50	10	55	28	45	65	40
	15	60	28	46	68	54
	20	59	29	44	71	55
	25	62	28	45	73	61
	30	61	29	43	76	67
HPNP_40	10	66	21	54	65	50
	15	70	20	57	67	60
	20	70	22	54	69	65
	25	73	21	55	72	72
	30	72	23	54	74	75
HPMP_40	10	61	25	49	65	47
	15	65	23	51	68	55
	20	65	25	50	70	61
	25	68	24	51	73	65
	30	67	27	50	74	72
HPHP_40	10	51	33	40	65	41
	15	54	32	41	68	50
	20	55	33	41	70	53
	25	56	33	42	73	58
	30	55	35	40	75	58

Table 11: Simulation results for the 2-bound design with equal allocation in the expansion phase. Results implement a 75% calibration rule and cover the null and high predictive scenarios.

Scenario	Post-Interim SS	T1E/Power	% SNP	% SP	Average N	% PoS post
NPNPT	10	15	75	12	63	25
	15	14	75	11	64	24
	20	15	75	12	65	22
	25	15	75	13	66	20
	30	15	75	12	68	21
NPPT	10	12	77	10	63	18
	15	15	75	12	64	22
	20	13	77	11	65	23
	25	14	76	12	66	21
	30	13	77	11	67	18
NPNPNT	10	14	76	10	63	24
	15	15	75	11	64	28
	20	13	77	10	65	23
	25	15	75	11	67	23
	30	14	77	11	67	23
NPPNT	10	14	77	11	63	24
	15	14	77	11	64	22
	20	14	76	11	65	23
	25	15	76	12	66	23
	30	14	77	11	67	23
HPNP_50	10	80	15	71	63	65
	15	82	14	71	64	75
	20	83	14	72	65	81
	25	85	14	74	66	89
	30	83	15	71	68	90
HPMP_50	10	71	22	61	63	58
	15	75	19	63	65	69
	20	76	20	63	67	76
	25	78	19	66	68	80
	30	75	22	62	69	83
HPHP_50	10	55	33	45	64	43
	15	59	33	47	66	59
	20	57	34	45	68	59
	25	60	33	48	70	66
	30	60	34	45	73	71
HPNP_40	10	65	25	55	64	53
	15	69	25	58	65	63
	20	68	26	56	67	68
	25	71	25	58	69	75
	30	69	27	55	71	79
HPMP_40	10	61	30	50	64	52
	15	63	28	52	66	58
	20	63	30	51	68	65
	25	66	29	53	69	70
	30	64	31	51	71	74
HPHP_40	10	50	38	41	64	44
	15	52	38	42	66	53
	20	53	38	42	68	56
	25	55	38	44	69	62
	30	53	40	41	71	62

Table 12: Simulation results for the 2-bound design with equal allocation in the expansion phase. Results implement a 80% calibration rule and cover the null and high predictive scenarios.

Scenario	Post-Interim SS	T1E/Power	% SNP	% SP	Average N	PoS post
NPNPT	10	15	80	12	62	34
	15	14	81	12	62	30
	20	15	80	13	63	28
	25	15	80	14	63	22
	30	15	80	13	64	24
NPPT	10	13	82	11	61	23
	15	15	80	13	62	29
	20	13	82	11	63	27
	25	14	81	13	63	24
	30	13	82	12	64	22
NPNPNT	10	13	81	11	62	27
	15	15	80	13	62	32
	20	13	82	11	63	28
	25	14	81	12	64	28
	30	14	81	12	64	25
NPPNT	10	14	81	12	61	33
	15	14	82	12	62	28
	20	13	82	12	62	27
	25	15	81	13	63	26
	30	13	82	12	64	27
HPNP_50	10	78	20	72	62	74
	15	80	18	73	63	83
	20	80	19	73	63	83
	25	81	18	75	64	92
	30	80	20	72	65	92
HPMP_50	10	70	27	63	62	64
	15	74	24	66	63	76
	20	72	26	65	64	79
	25	75	24	68	64	84
	30	72	27	64	66	85
HPHP_50	10	53	40	47	63	50
	15	57	39	50	63	65
	20	55	41	47	65	66
	25	57	39	50	65	72
	30	56	41	47	68	74
HPNP_40	10	63	32	56	62	60
	15	67	30	60	63	70
	20	64	33	57	64	74
	25	68	30	60	65	78
	30	65	33	57	66	82
HPMP_40	10	60	36	52	63	60
	15	62	34	54	63	67
	20	60	37	52	64	70
	25	63	34	55	65	76
	30	61	37	52	66	80
HPHP_40	10	49	45	43	62	54
	15	50	45	44	63	58
	20	51	45	44	64	62
	25	52	44	46	65	67
	30	50	46	43	66	69

3.2 Unequal Allocation in Expansion Phase

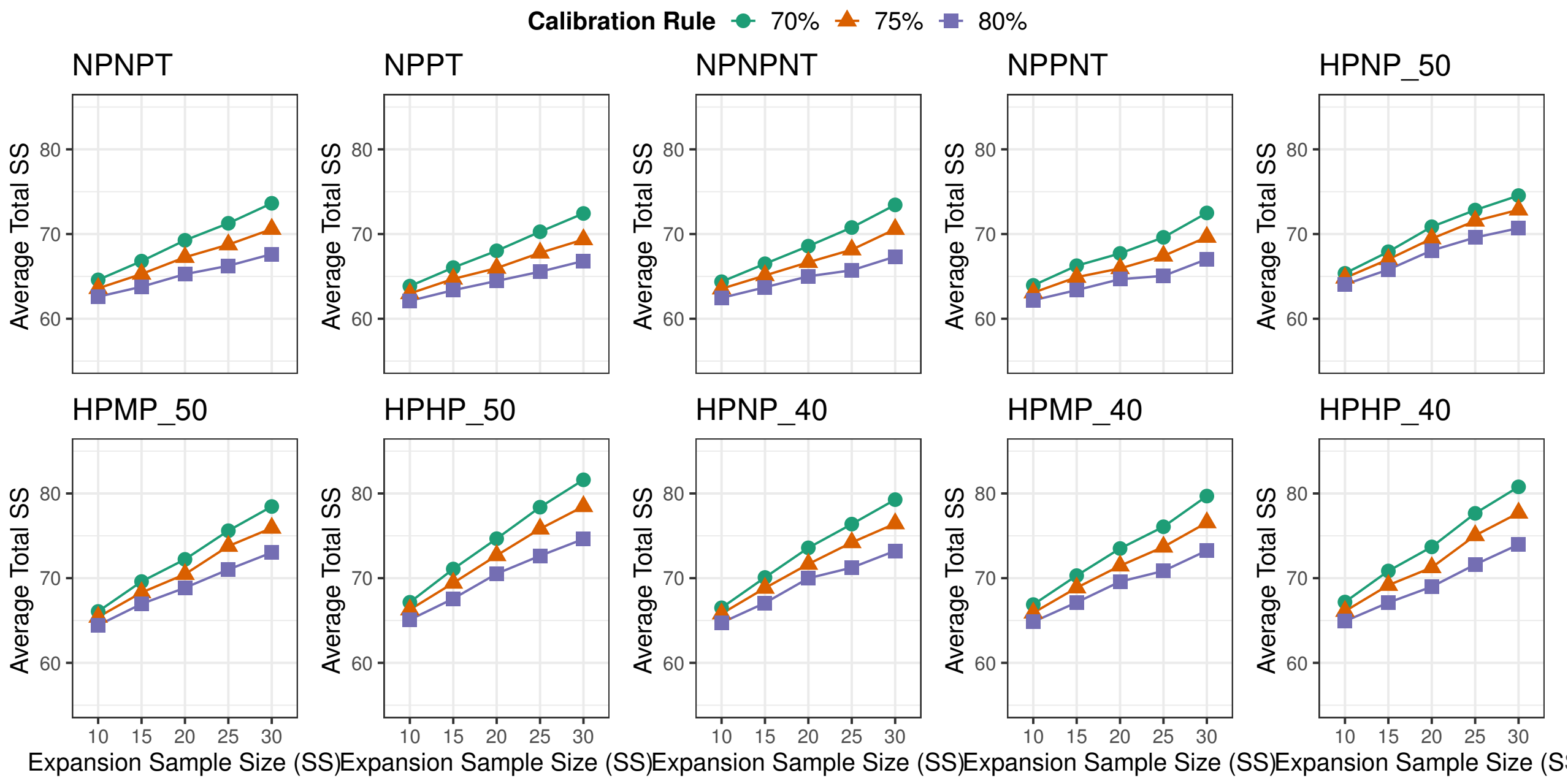

Figure 16: Average sample size under the 3-Bound configuration for the unequal allocation in the expansion phase. Results are provided for three different calibration rules: 70%, 75% and 80% and different expansion sample sizes: $n_{0,2} : n_{1,2} = 0 : 20, 10 : 30, 15 : 35, 20 : 40$.

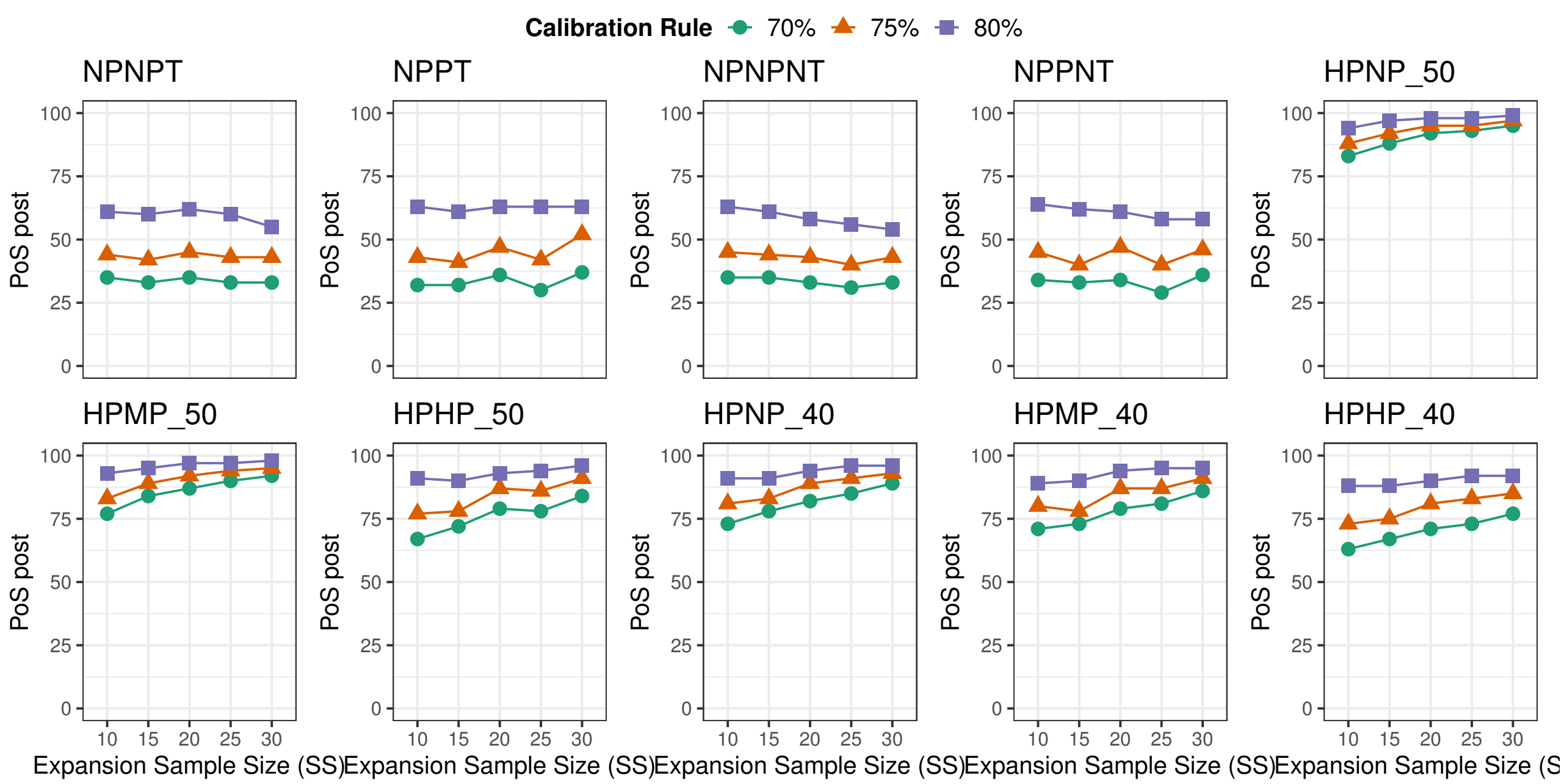

Figure 17: PoS post under the 3-Bound configuration for the unequal allocation in the expansion phase. Results are provided for three different calibration rules: 70%, 75% and 80% and different expansion sample sizes: $n_{0,2} : n_{1,2} = 0 : 20, 10 : 30, 15 : 35, 20 : 40$.

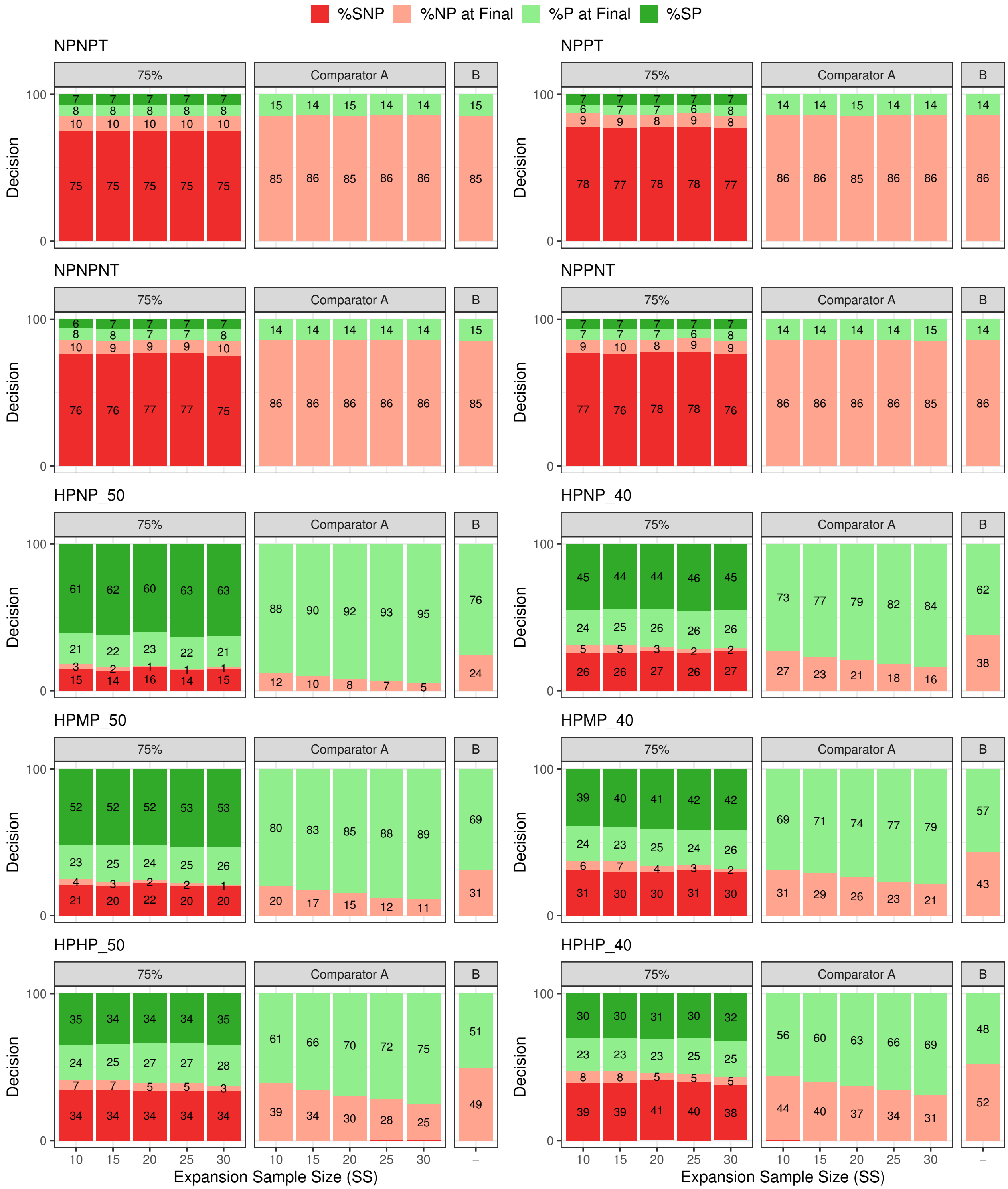

Figure 18: Percentage of simulations under unequal allocation (5000 replications) and 3-Bound configuration that resulted in each decision category: predictive (%SP, dark green) or non-predictive (%SNP, dark red) at interim, or predictive (%P at Final, light green) or non-predictive (%NP at Final, light red) at final analysis. Results are by expansion sample size, $n_{0,2} : n_{1,2} = 0 : 20, 10 : 30, 15 : 35, 20 : 40$, under a 75% calibration rule. Comparator A is the design with no-interim but total sample size matched to the interim design. Comparator B is the no-interim design with 40:20 allocation.

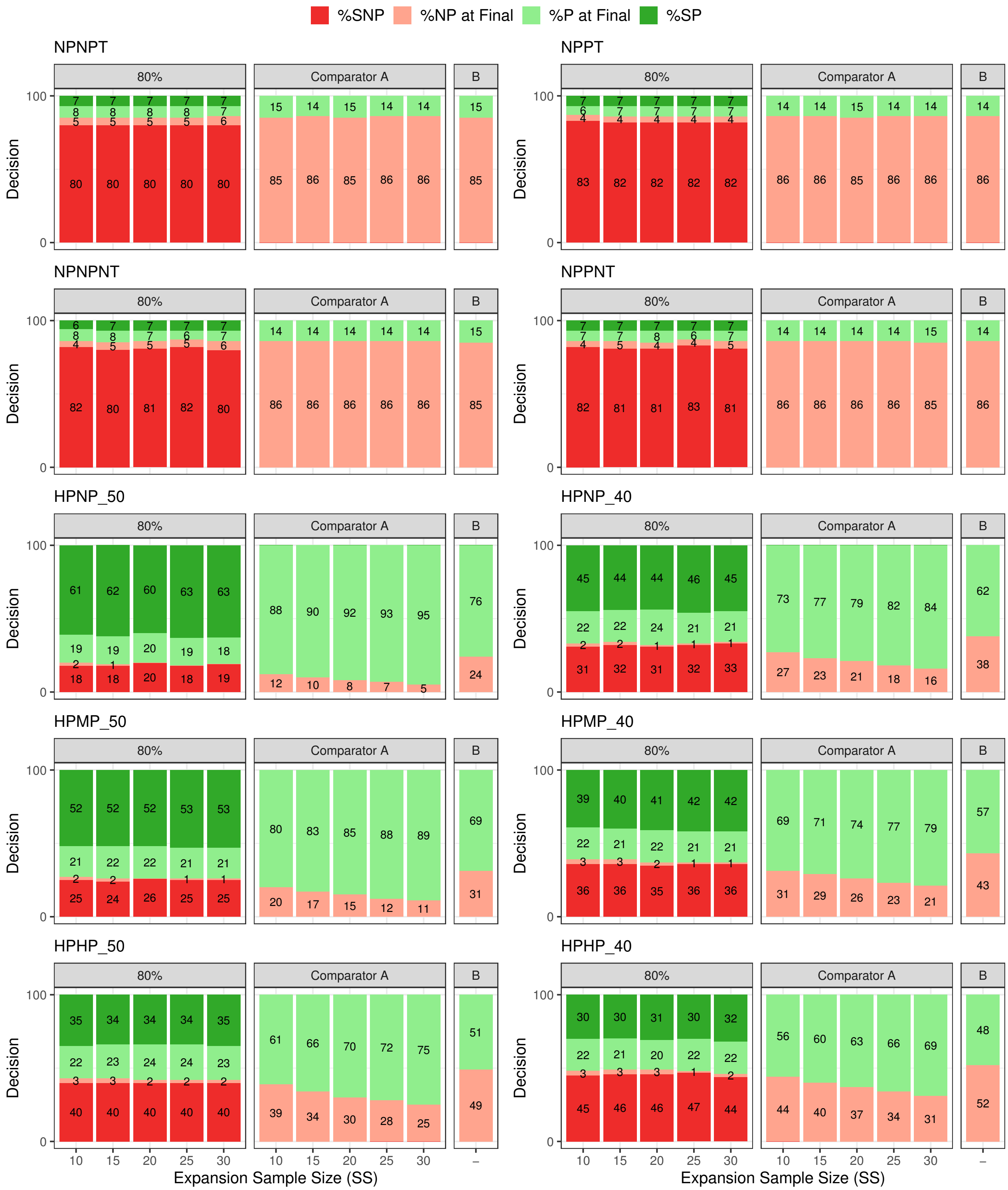

Figure 19: Percentage of simulations under unequal allocation (5000 replications) and 3-Bound configuration that resulted in each decision category: predictive (%SP, dark green) or non-predictive (%SNP, dark red) at interim, or predictive (%P at Final, light green) or non-predictive (%NP at Final, light red) at final analysis. Results are by expansion sample size, $n_{0,2} : n_{1,2} = 0 : 20, 10 : 30, 15 : 35, 20 : 40$, under a 80% calibration rule. Comparator A is the design with no-interim but total sample size matched to the interim design. Comparator B is the no-interim design with 40:20 allocation.

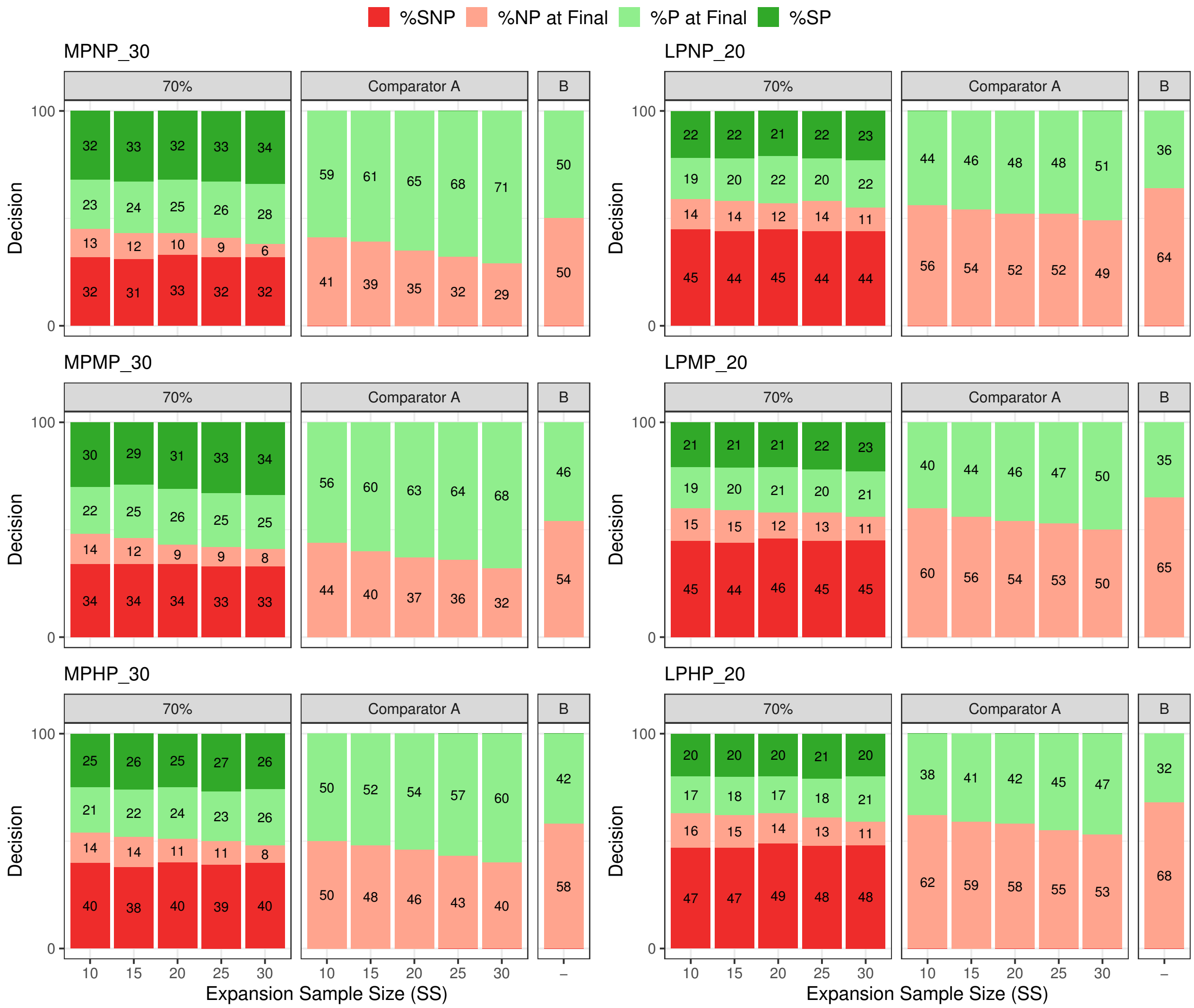

Figure 20: Percentage of simulations under unequal allocation (5000 replications) and 3-Bound configuration that resulted in each decision category: predictive (%SP, dark green) or non-predictive (%SNP, dark red) at interim, or predictive (%P at Final, light green) or non-predictive (%NP at Final, light red) at final analysis. Results are by expansion sample size, $n_{0,2} : n_{1,2} = 0 : 20, 10 : 30, 15 : 35, 20 : 40$, under a 70% calibration rule. Comparator A is the design with no-interim but total sample size matched to the interim design. Comparator B is the no-interim design with 40:20 allocation.

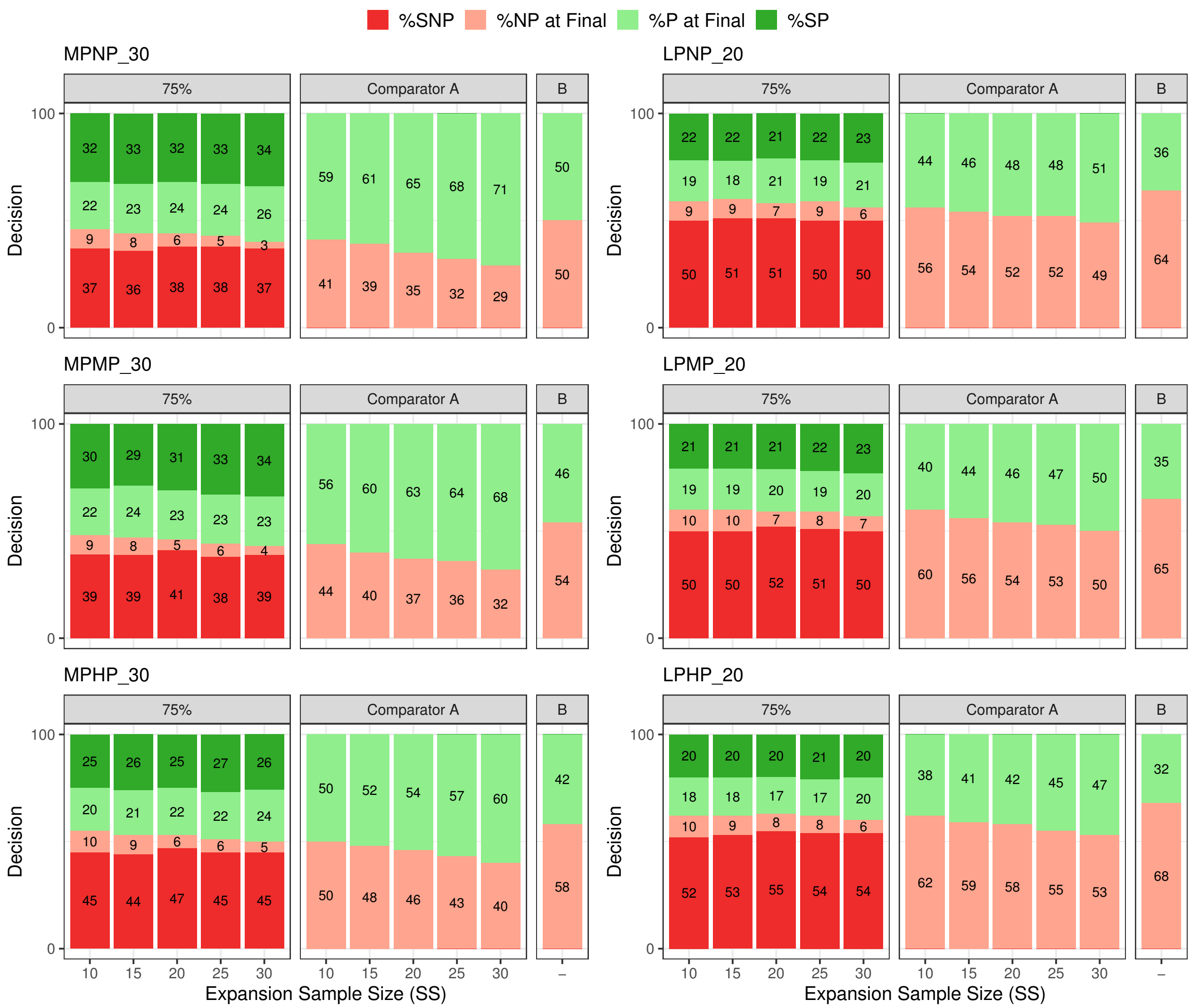

Figure 21: Percentage of simulations under unequal allocation (5000 replications) and 3-Bound configuration that resulted in each decision category: predictive (%SP, dark green) or non-predictive (%SNP, dark red) at interim, or predictive (%P at Final, light green) or non-predictive (%NP at Final, light red) at final analysis. Results are by expansion sample size, $n_{0,2} : n_{1,2} = 0 : 20, 10 : 30, 15 : 35, 20 : 40$, under a 75% calibration rule. Comparator A is the design with no-interim but total sample size matched to the interim design. Comparator B is the no-interim design with 40:20 allocation.

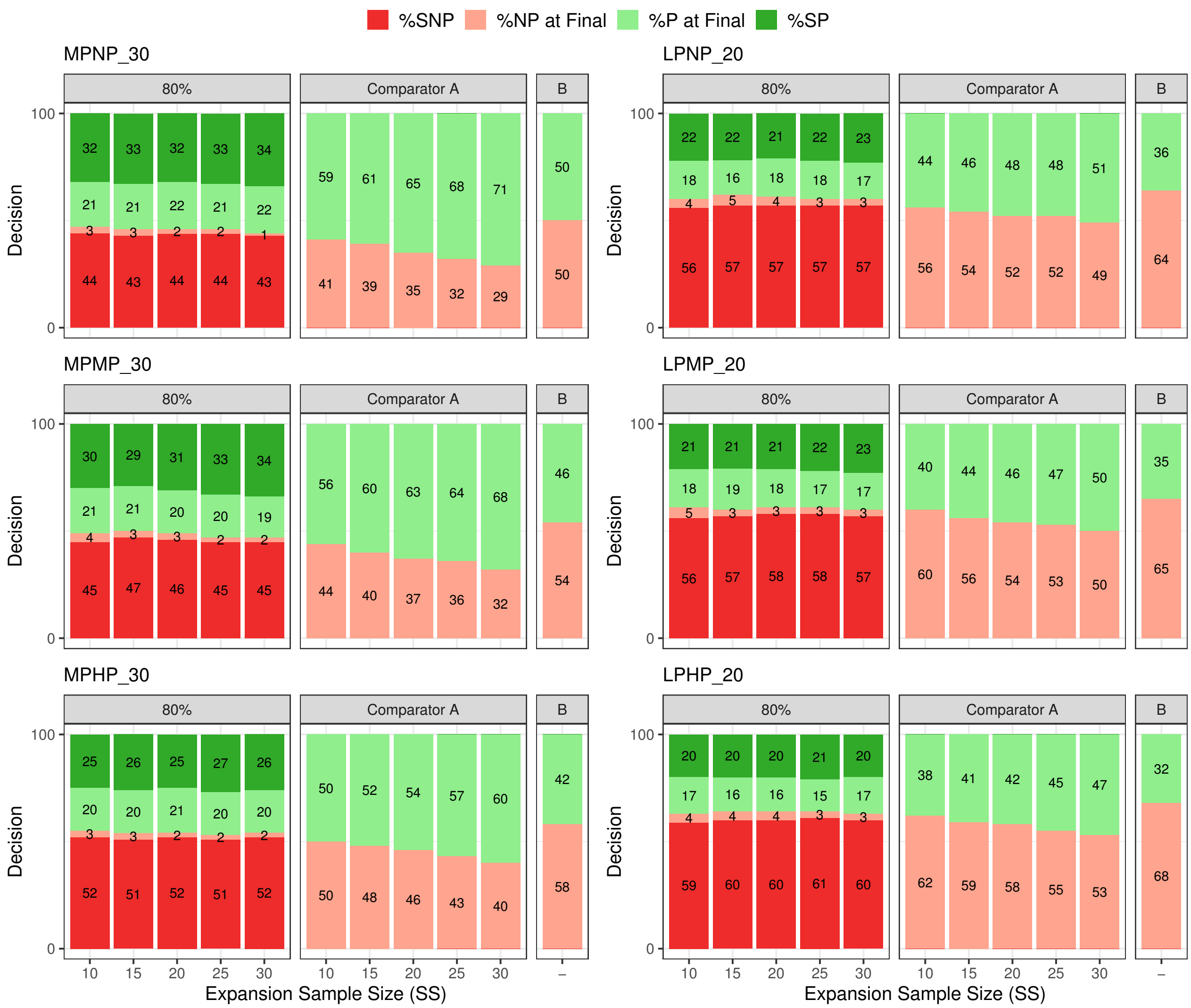

Figure 22: Percentage of simulations under unequal allocation (5000 replications) and 3-Bound configuration that resulted in each decision category: predictive (%SP, dark green) or non-predictive (%SNP, dark red) at interim, or predictive (%P at Final, light green) or non-predictive (%NP at Final, light red) at final analysis. Results are by expansion sample size, $n_{0,2} : n_{1,2} = 0 : 20, 10 : 30, 15 : 35, 20 : 40$, under a 80% calibration rule. Comparator A is the design with no-interim but total sample size matched to the interim design. Comparator B is the no-interim design with 40:20 allocation.

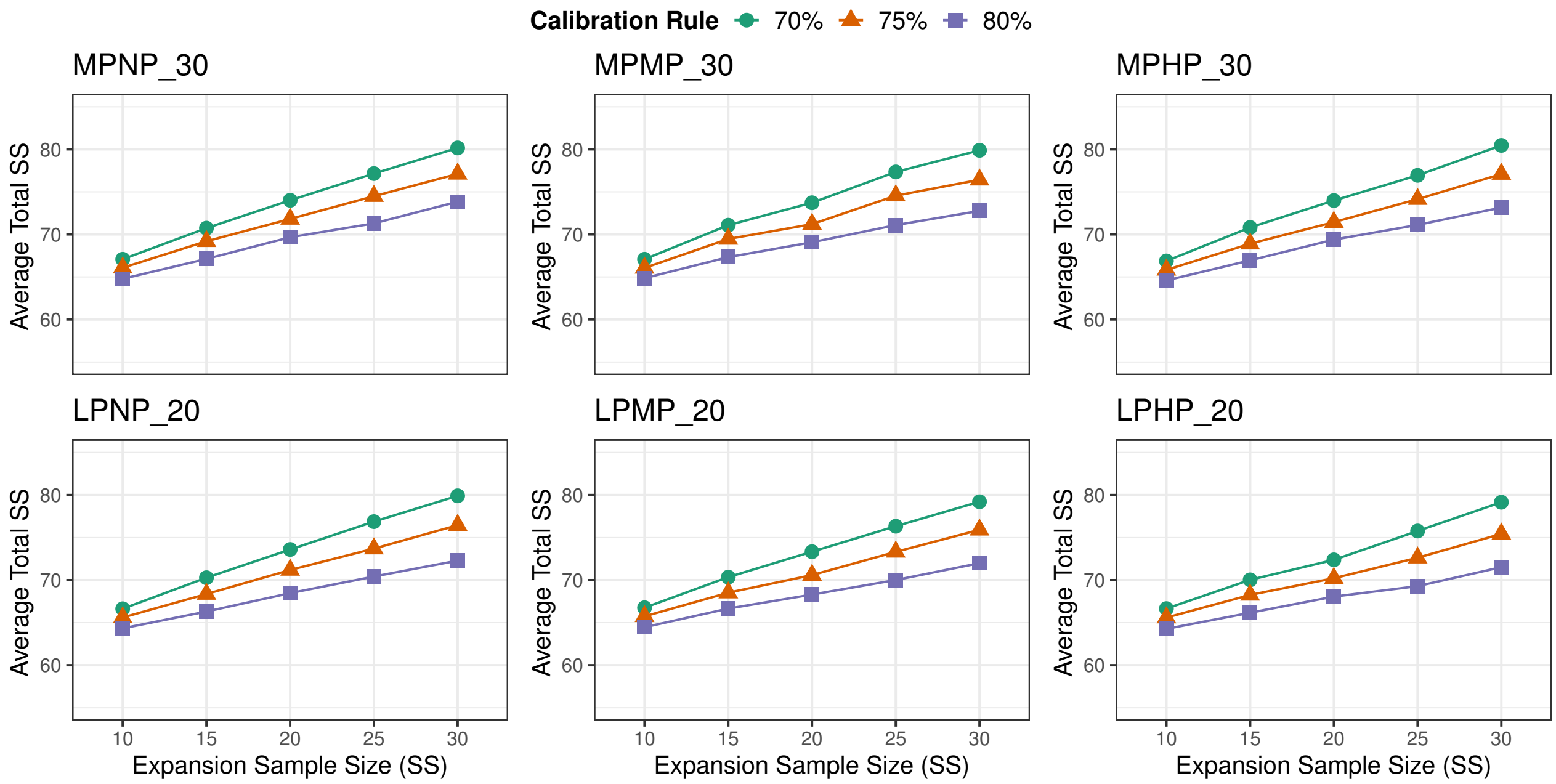

Figure 23: Average sample size under the 3-Bound configuration for the unequal allocation in the expansion phase. Results are provided for three different calibration rules: 70%, 75% and 80% and different expansion sample sizes: $n_{0,2} : n_{1,2} = 0 : 20, 10 : 30, 15 : 35, 20 : 40$.

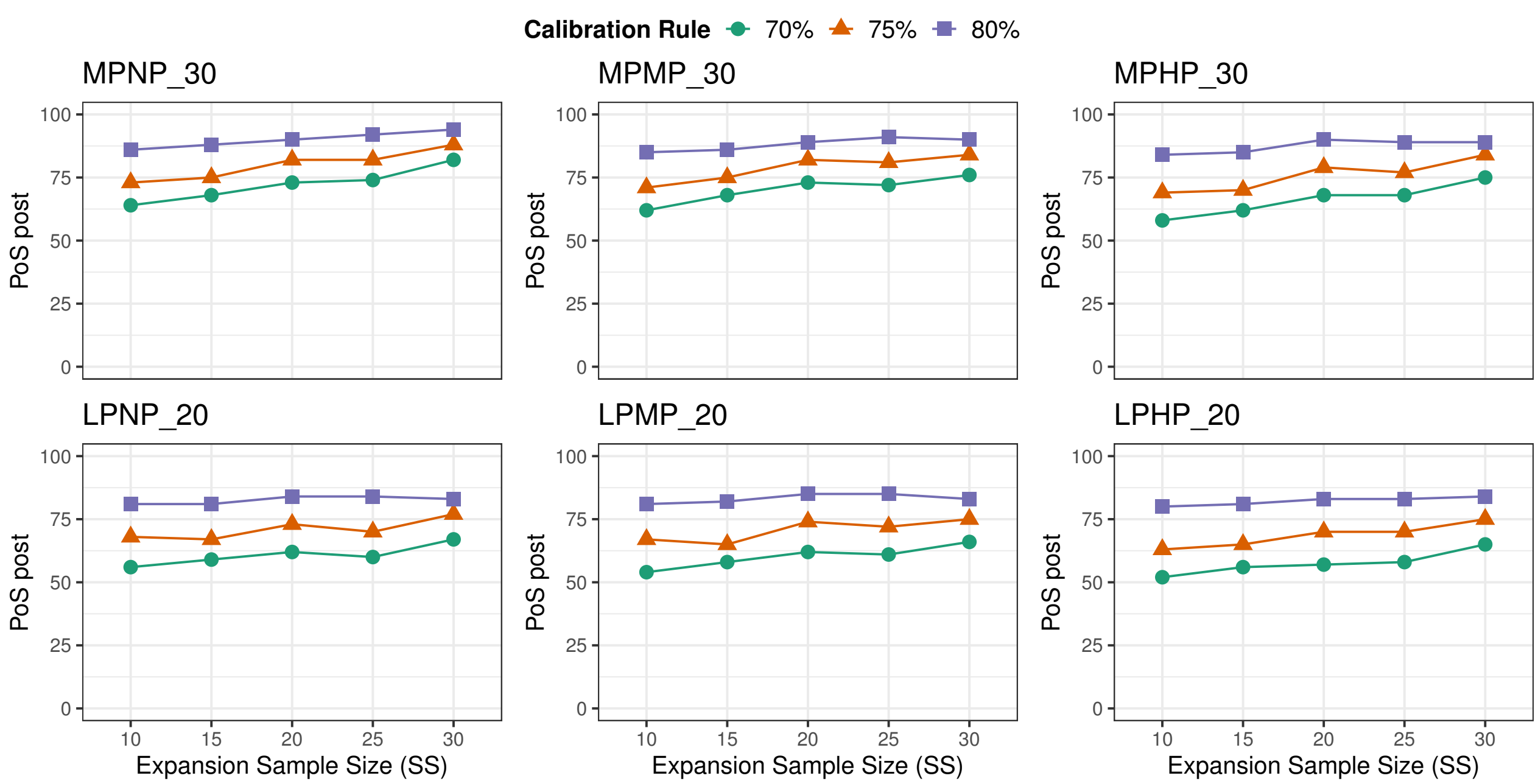

Figure 24: PoS post under the 3-Bound configuration for the unequal allocation in the expansion phase. Results are provided for three different calibration rules: 70%, 75% and 80% and different expansion sample sizes: $n_{0,2} : n_{1,2} = 0 : 20, 10 : 30, 15 : 35, 20 : 40$.

3.3 Overall Comparison

Table 13: Average power, total N, PoS post, % SNP and % SP metrics across the high predictive scenarios under the 2-Bound and 3-Bound configurations for both equal allocation and unequal allocation after expansion. Mean type I error rate under the null scenarios. Three calibration rules are considered: 80%, 75% or 70%. The preferred approach is highlighted in **bold**, which balances power gain and an increase in PoS post-interim

Metric	Bounds	Calibration Rule	Equal Allocation ($n_{0,2} = n_{1,2}$)					Unequal Allocation (N)				
			10	15	20	25	30	80	90	100	110	120
Mean Type I Error	2-Bound	80%	14	15	14	15	14	14	14	15	14	15
		75%	14	15	14	15	14	14	15	14	14	14
		70%	14	15	14	14	14	14	15	14	14	15
	3-Bound	80%	15	14	15	14	14	14	15	15	14	14
		75%	15	15	15	14	15	14	15	14	14	15
		70%	**15**	**15**	**14**	**14**	**15**	**14**	**15**	**14**	**14**	**15**
Mean Power	2-Bound	80%	62	65	64	66	64	64	64	64	65	65
		75%	64	67	67	69	67	65	66	66	68	69
		70%	64	68	69	71	70	66	67	68	70	71
	3-Bound	80%	65	65	67	66	66	65	65	66	66	66
		75%	66	68	69	69	70	67	68	68	70	70
		70%	**67**	**70**	**70**	**71**	**73**	**67**	**69**	**70**	**71**	**73**
Mean Average N	2-Bound	80%	62	63	72	78	80	60	61	62	63	64
		75%	64	66	67	68	71	60	62	63	65	68
		70%	65	67	69	72	74	60	62	65	67	69
	3-Bound	80%	65	67	69	71	73	65	67	69	71	73
		75%	66	68	71	74	76	66	69	71	74	76
		70%	**67**	**70**	**73**	**76**	**79**	**67**	**70**	**73**	**76**	**79**
Mean PoS post	2-Bound	80%	60	70	72	78	80	62	69	73	78	81
		75%	53	63	68	74	77	57	63	69	74	77
		70%	49	59	65	70	74	52	59	65	70	75
	3-Bound	80%	89	93	93	95	96	91	92	94	95	96
		75%	77	83	85	89	93	80	83	89	89	92
		70%	**71**	**77**	**78**	**83**	**88**	**72**	**77**	**82**	**83**	**87**
Mean % SNP	2-Bound	80%	33	32	34	32	34	33	33	33	33	33
		75%	27	26	26	26	28	28	27	28	28	27
		70%	23	22	23	22	24	23	23	24	23	23
	3-Bound	80%	32	33	32	33	33	33	33	33	33	33
		75%	27	27	27	28	28	28	27	28	28	27
		70%	**23**	**23**	**22**	**23**	**23**	**23**	**23**	**24**	**23**	**23**
Mean % SP	2-Bound	80%	56	58	56	59	56	58	56	57	57	58
		75%	54	56	55	57	54	56	55	55	55	55
		70%	53	55	54	55	53	55	53	54	54	54
	3-Bound	80%	44	45	46	45	45	44	44	44	45	45
		75%	44	45	46	45	45	44	44	44	45	45
		70%	**44**	**45**	**46**	**45**	**45**	**44**	**44**	**44**	**45**	**45**

3.4 Sensitivity Analysis

3.4.1 Interim Sample Sizes Results

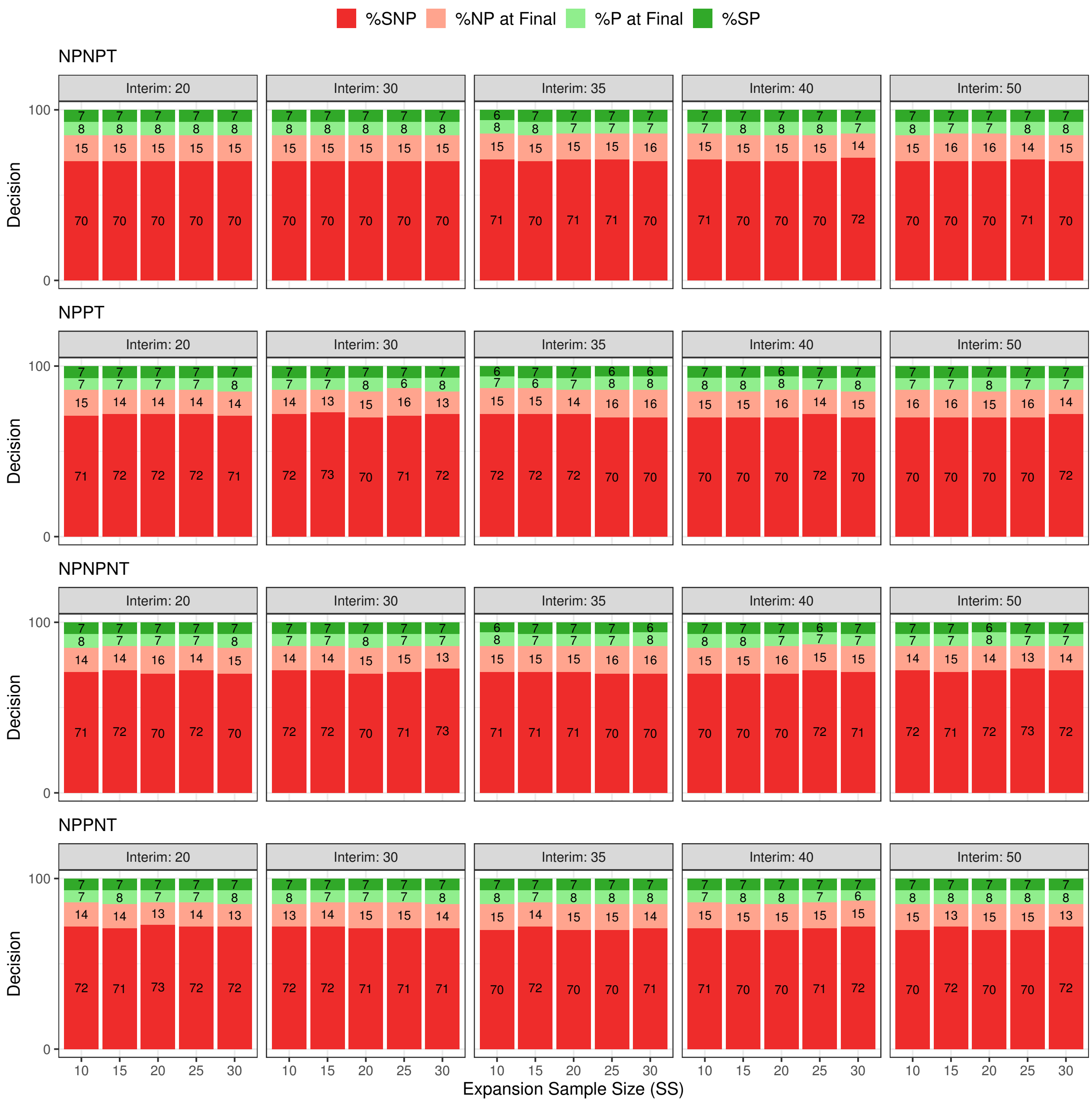

Figure 25: Percentage of simulations under the null scenarios (5000 replications) that resulted in each decision category: predictive (%SP, dark green) or non-predictive (%SNP, dark red) at interim, or predictive (%P at Final, light green) or non-predictive (%NP at Final, light red) at final analysis. Results are by interim sample size, $n_{0,1}$, and expansion sample size for a 3-Bound configuration and 70% calibration rule.

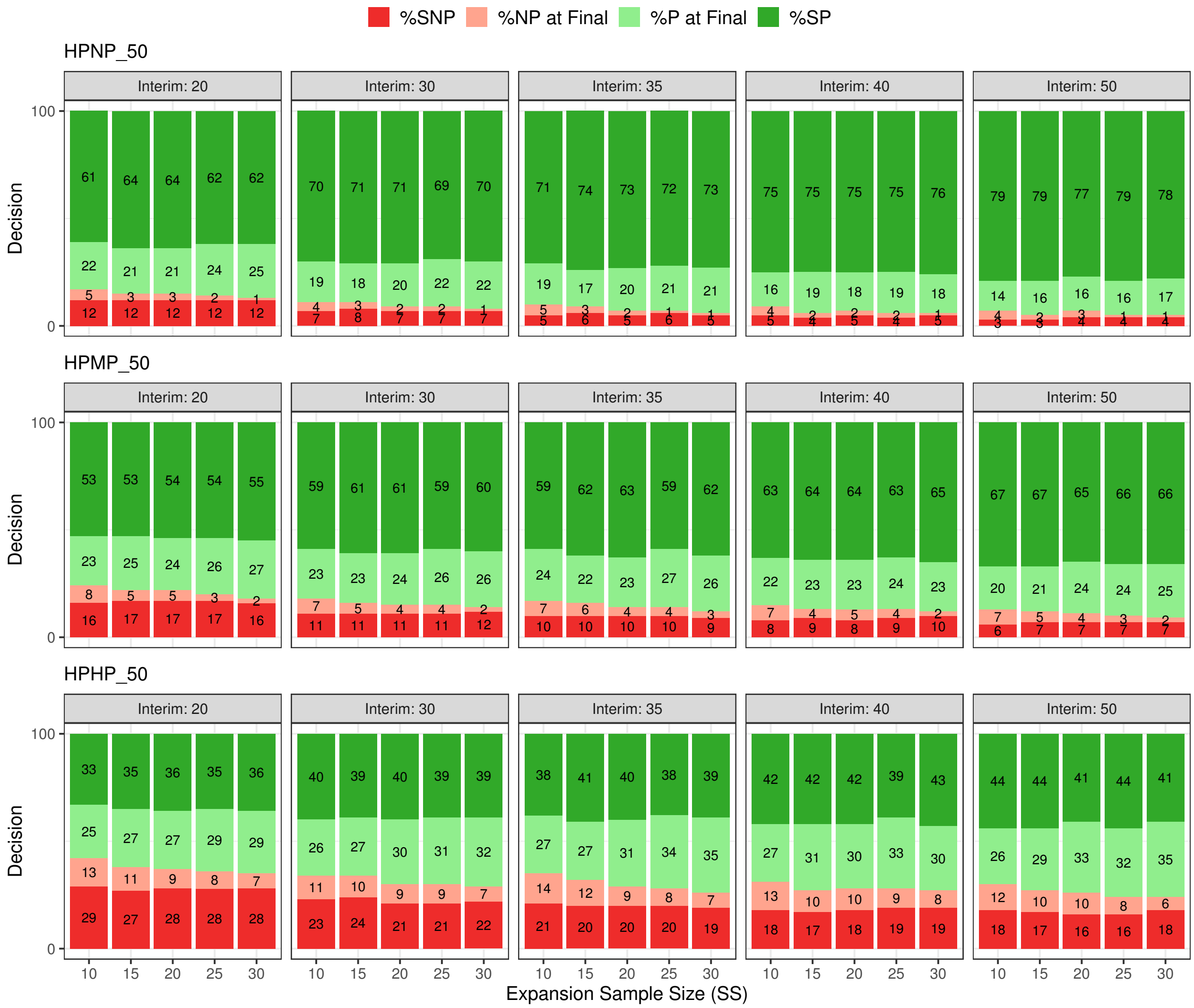

Figure 26: Percentage of simulations under the high predictive scenarios with 50% predictive effect (5000 replications) that resulted in each decision category: predictive (%SP, dark green) or non-predictive (%SNP, dark red) at interim, or predictive (%P at Final, light green) or non-predictive (%NP at Final, light red) at final analysis. Results are by interim sample size, $n_{0,1}$, and expansion sample size for a 3-Bound configuration and 70% calibration rule.

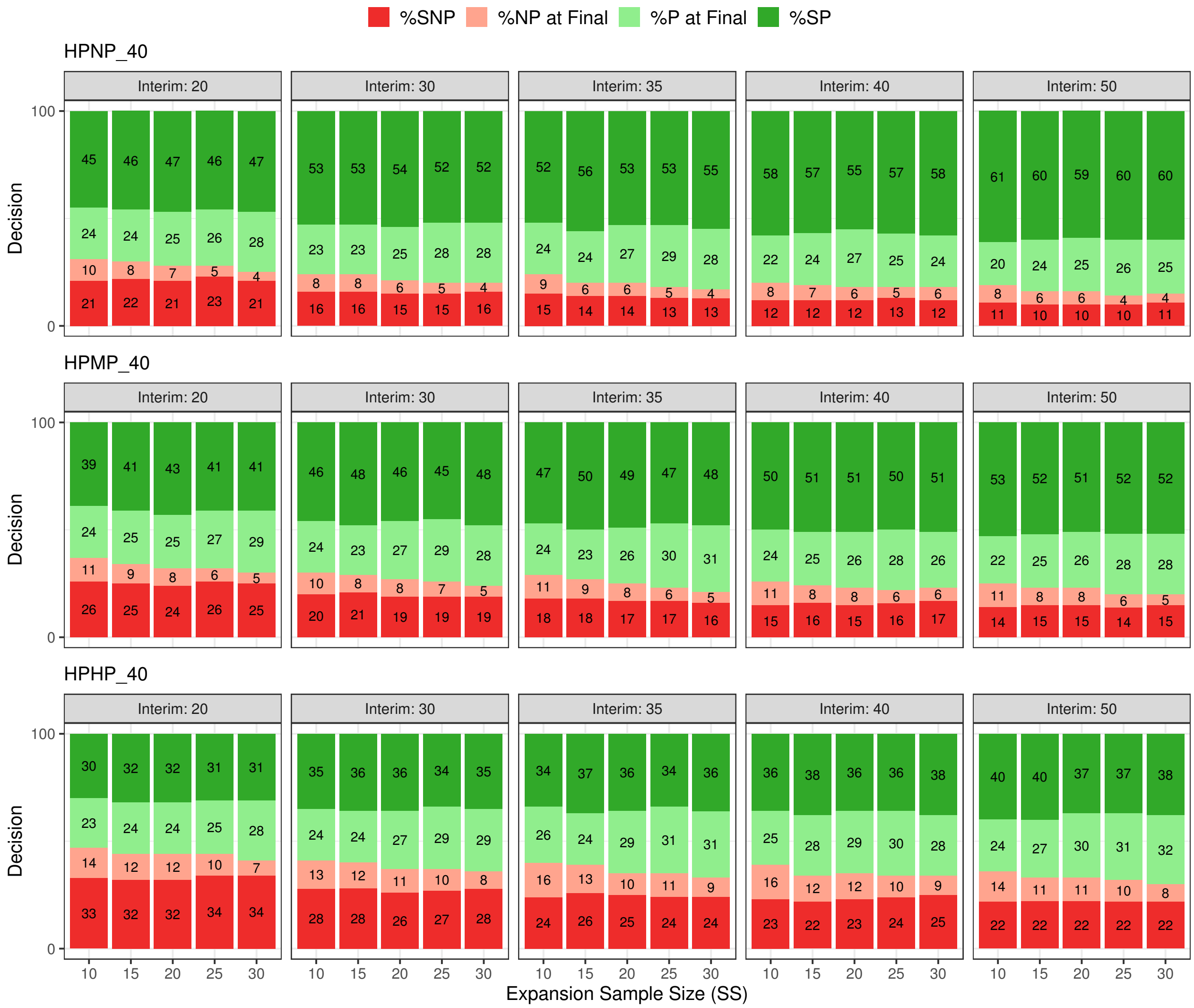

Figure 27: Percentage of simulations under the high predictive scenarios with 40% predictive effect (5000 replications) that resulted in each decision category: predictive (%SP, dark green) or non-predictive (%SNP, dark red) at interim, or predictive (%P at Final, light green) or non-predictive (%NP at Final, light red) at final analysis. Results are by interim sample size, $n_{0,1}$, and expansion sample size for a 3-Bound configuration and 70% calibration rule.

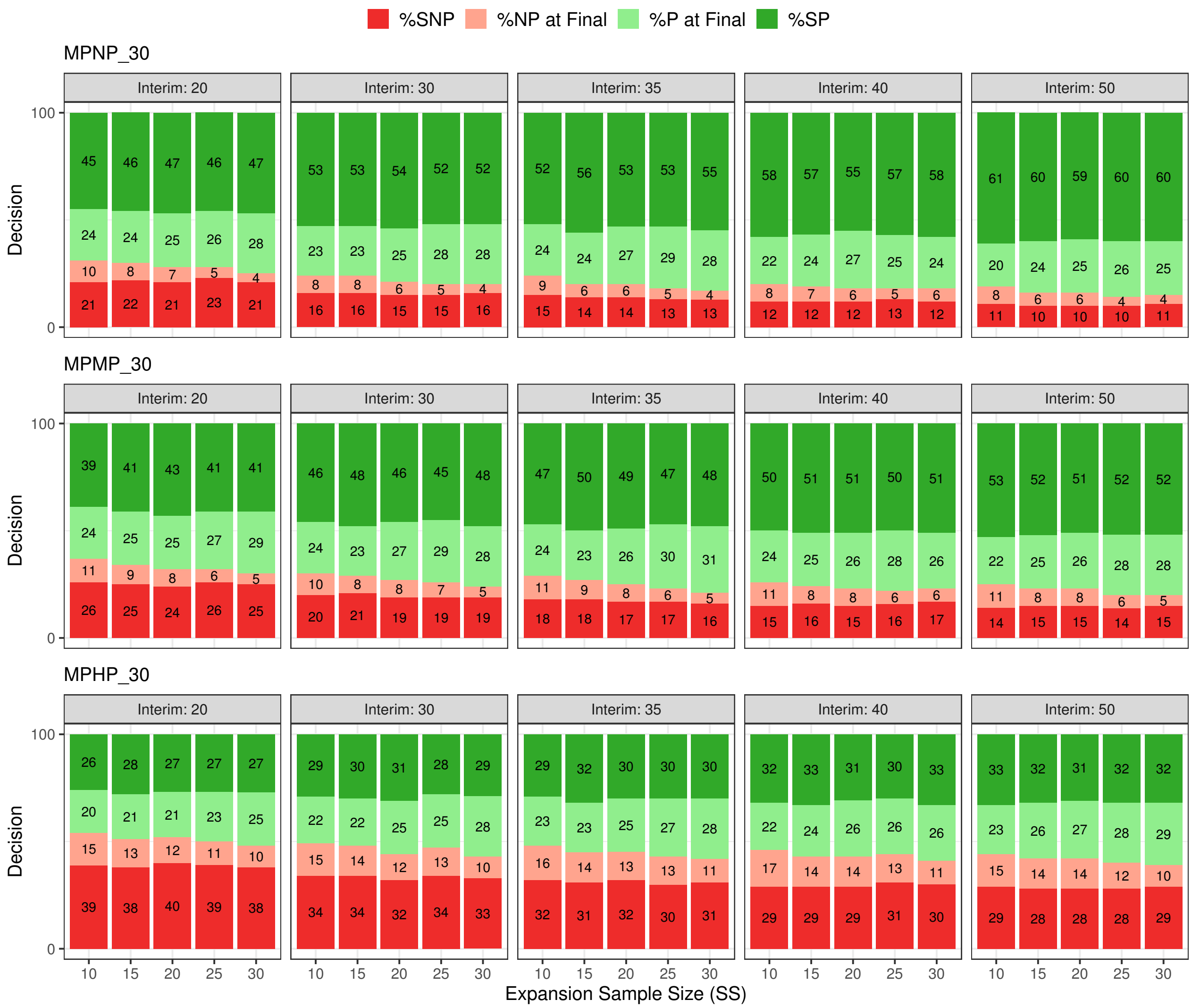

Figure 28: Percentage of simulations under the medium predictive scenarios with 30% predictive effect (5000 replications) that resulted in each decision category: predictive (%SP, dark green) or non-predictive (%SNP, dark red) at interim, or predictive (%P at Final, light green) or non-predictive (%NP at Final, light red) at final analysis. Results are by interim sample size, $n_{0,1}$, and expansion sample size for a 3-Bound configuration and 70% calibration rule.

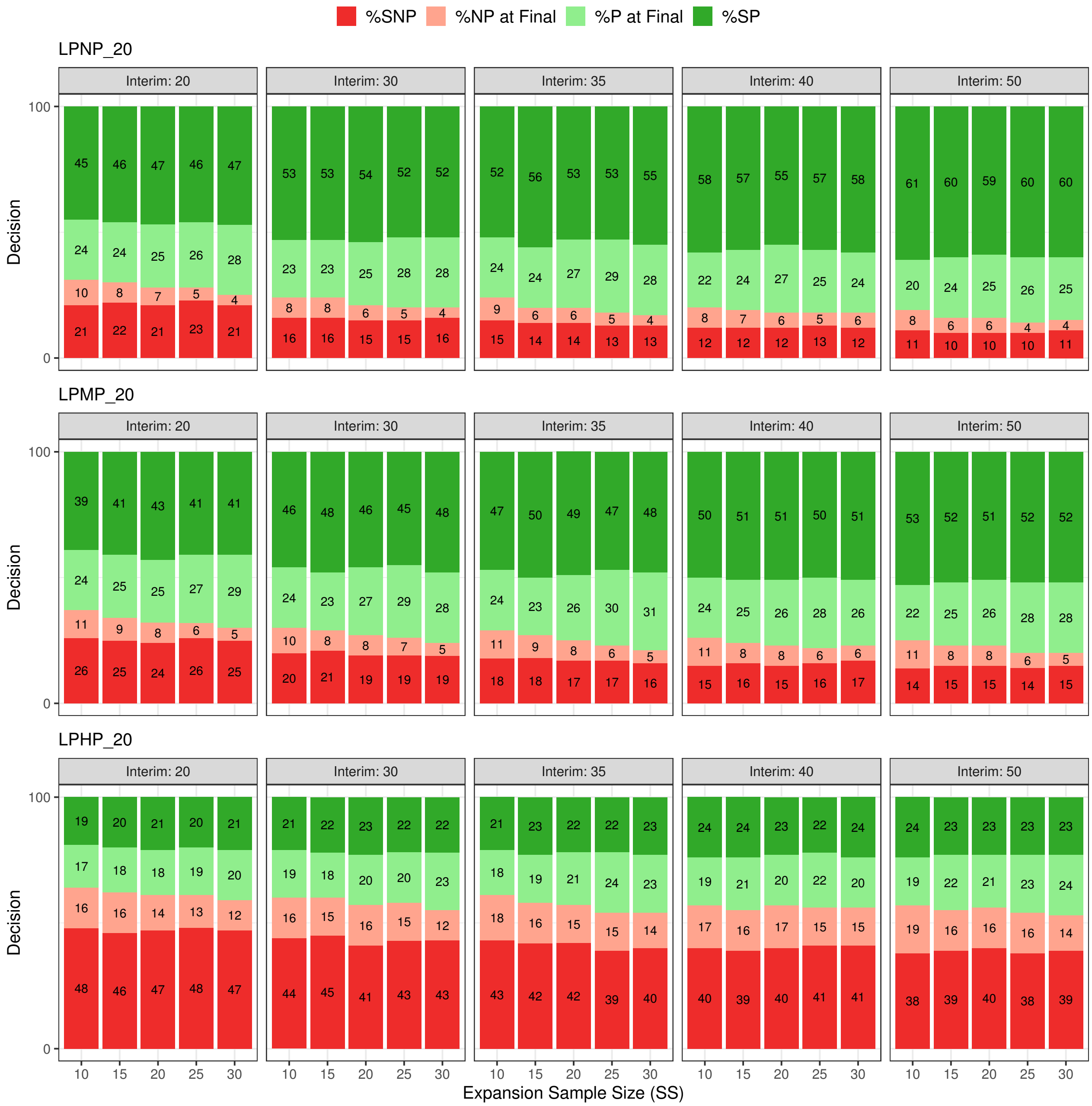

Figure 29: Percentage of simulations under the low predictive scenarios with 20% predictive effect (5000 replications) that resulted in each decision category: predictive (%SP, dark green) or non-predictive (%SNP, dark red) at interim, or predictive (%P at Final, light green) or non-predictive (%NP at Final, light red) at final analysis. Results are by interim sample size, $n_{0,1}$, and expansion sample size for a 3-Bound configuration and 70% calibration rule.

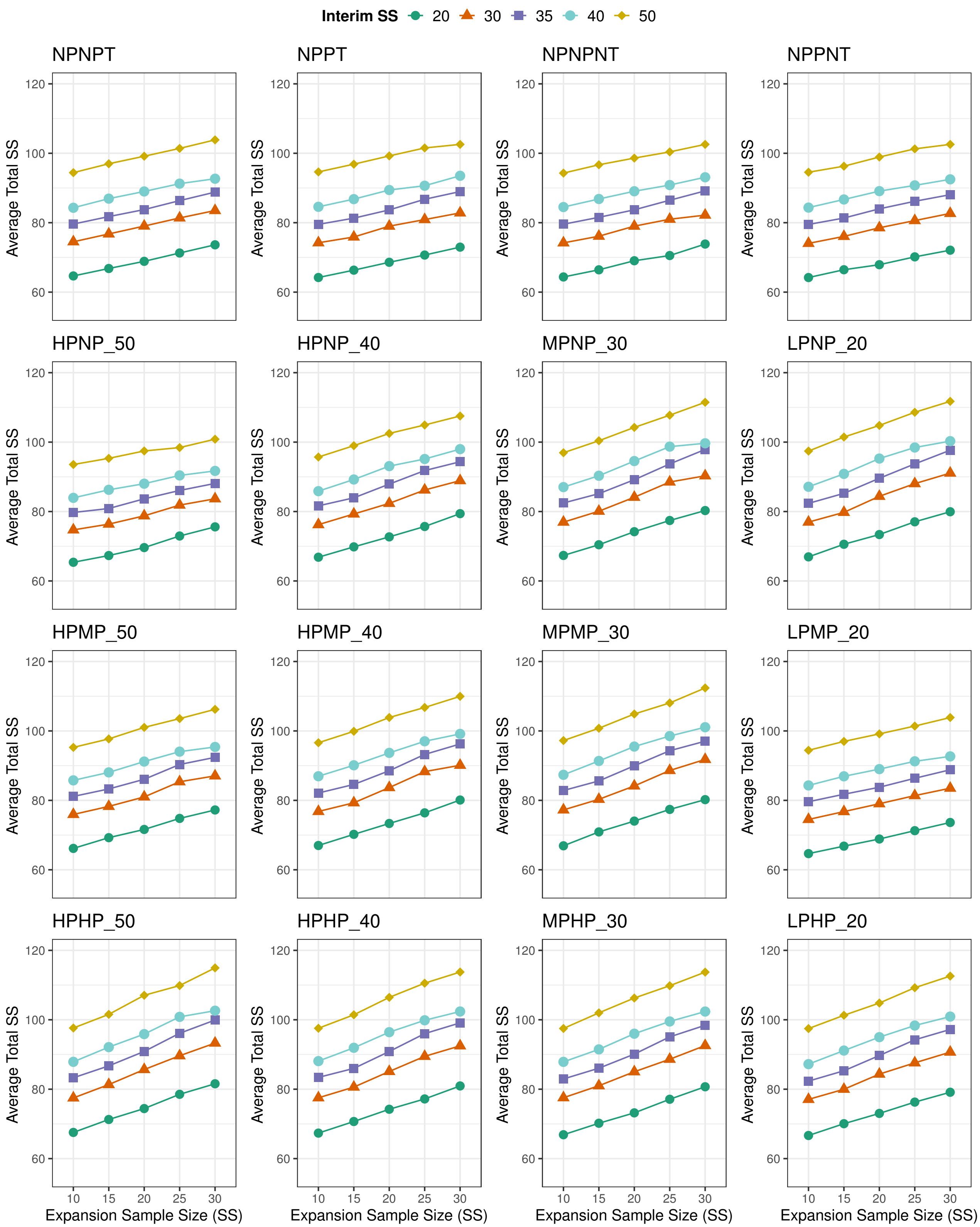

Figure 30: Average sample size under different interim control sample sizes, $n_{0,1}$ (5000 replications). Results are by expansion sample size for a 3-Bound configuration and 70% calibration rule.

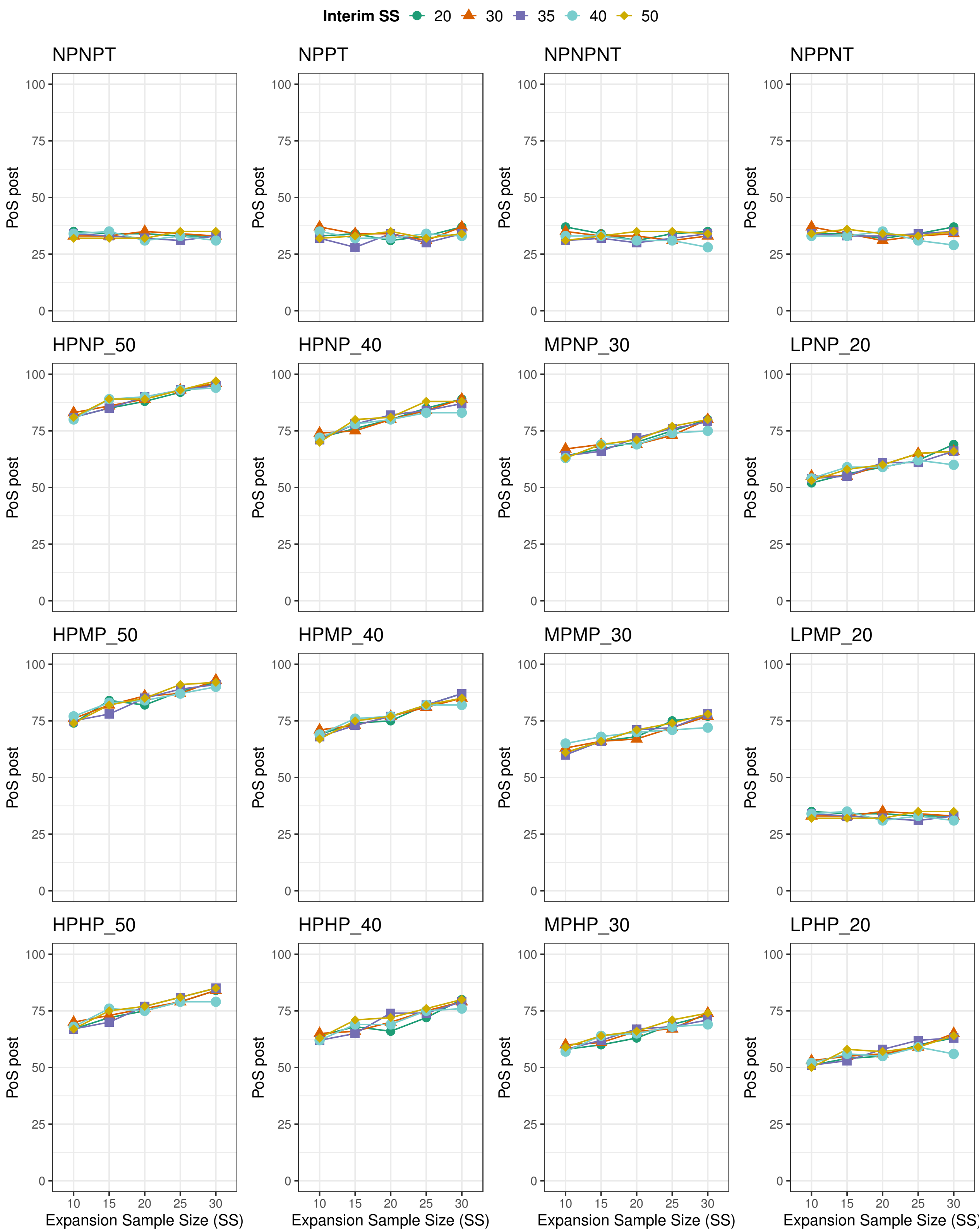

Figure 31: PoS post under different interim control sample sizes, $n_{0,1}$ (5000 replications). Results are by expansion sample size for a 3-Bound configuration and 70% calibration rule.

3.4.2 Biomarker Distribution

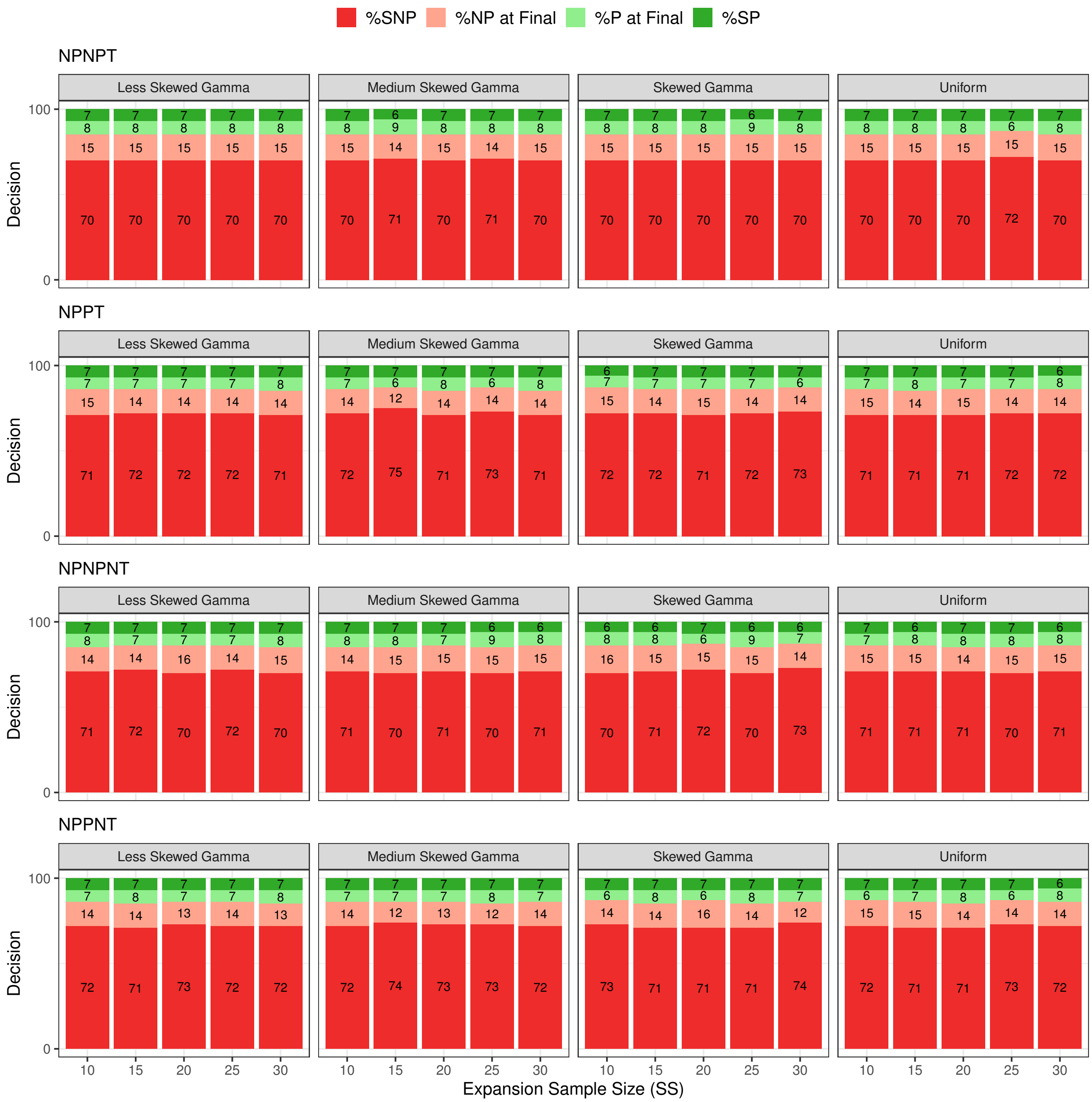

Figure 32: Percentage of simulations under the null scenarios (5000 replications) that resulted in each decision category: predictive (%SP, dark green) or non-predictive (%SNP, dark red) at interim, or predictive (%P at Final, light green) or non-predictive (%NP at Final, light red) at final analysis. Results are by biomarker distribution ("Less Skewed", "Medium Skewed" and "Skewed" Gamma and Uniform) and expansion sample size for a 3-Bound configuration and 70% calibration rule.

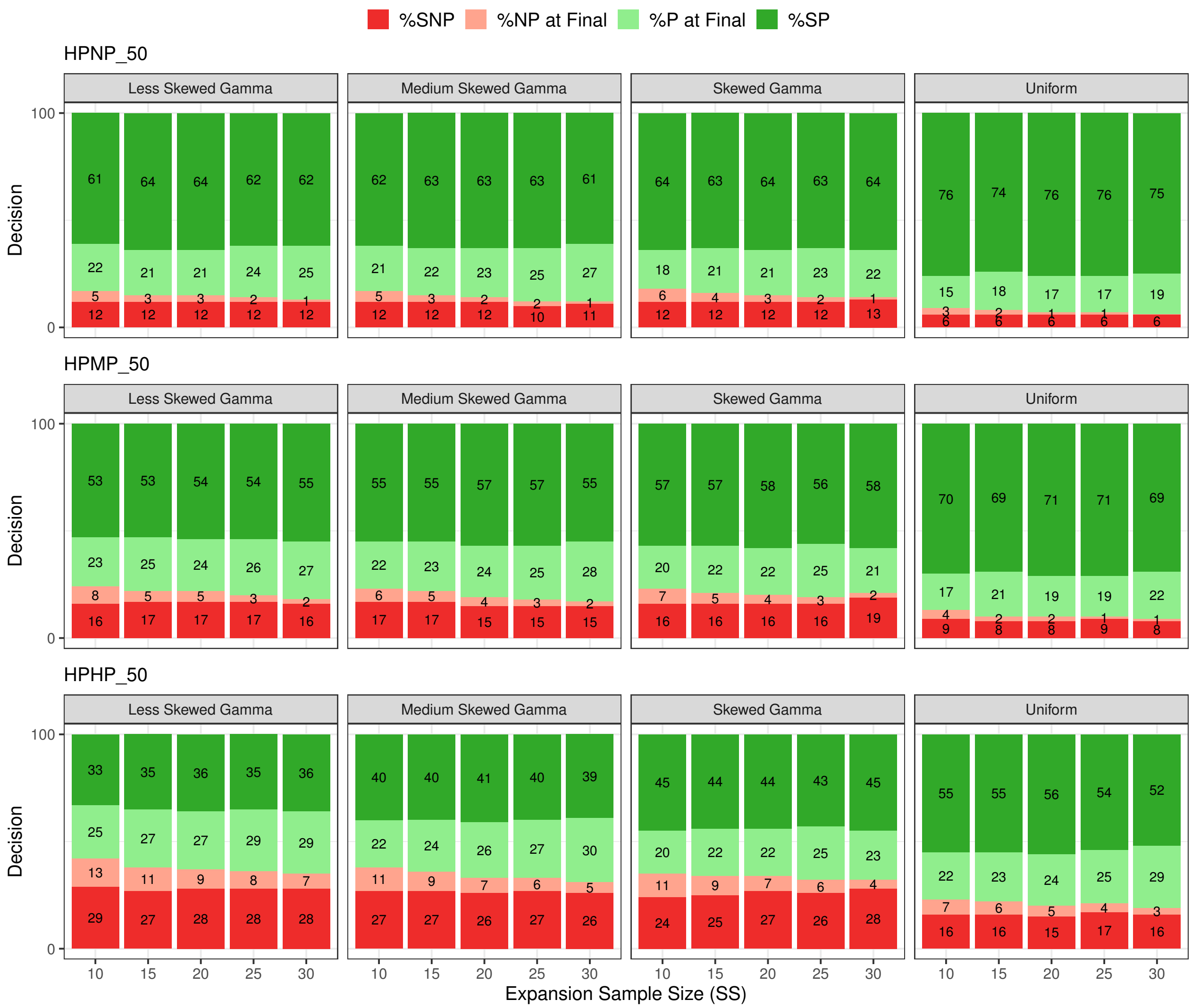

Figure 33: Percentage of simulations under the high predictive scenarios with 50% predictive effect (5000 replications) that resulted in each decision category: predictive (%SP, dark green) or non-predictive (%SNP, dark red) at interim, or predictive (%P at Final, light green) or non-predictive (%NP at Final, light red) at final analysis. Results are by biomarker distribution ("Less Skewed", "Medium Skewed" and "Skewed" Gamma and Uniform) and expansion sample size for a 3-Bound configuration and 70% calibration rule.

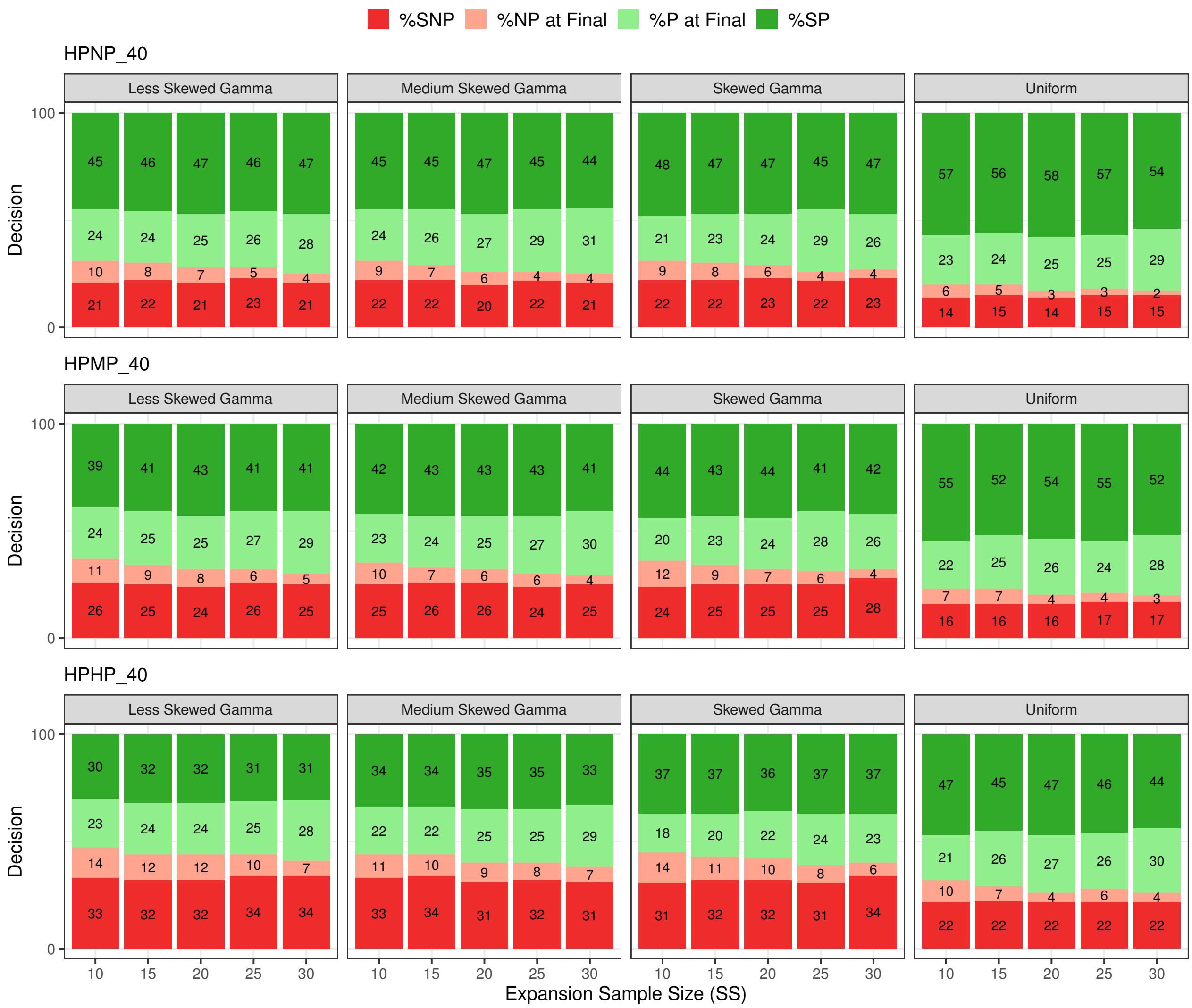

Figure 34: Percentage of simulations under the high predictive scenarios with 40% predictive effect (5000 replications) that resulted in each decision category: predictive (%SP, dark green) or non-predictive (%SNP, dark red) at interim, or predictive (%P at Final, light green) or non-predictive (%NP at Final, light red) at final analysis. Results are by biomarker distribution ("Less Skewed", "Medium Skewed" and "Skewed" Gamma and Uniform) and expansion sample size for a 3-Bound configuration and 70% calibration rule.

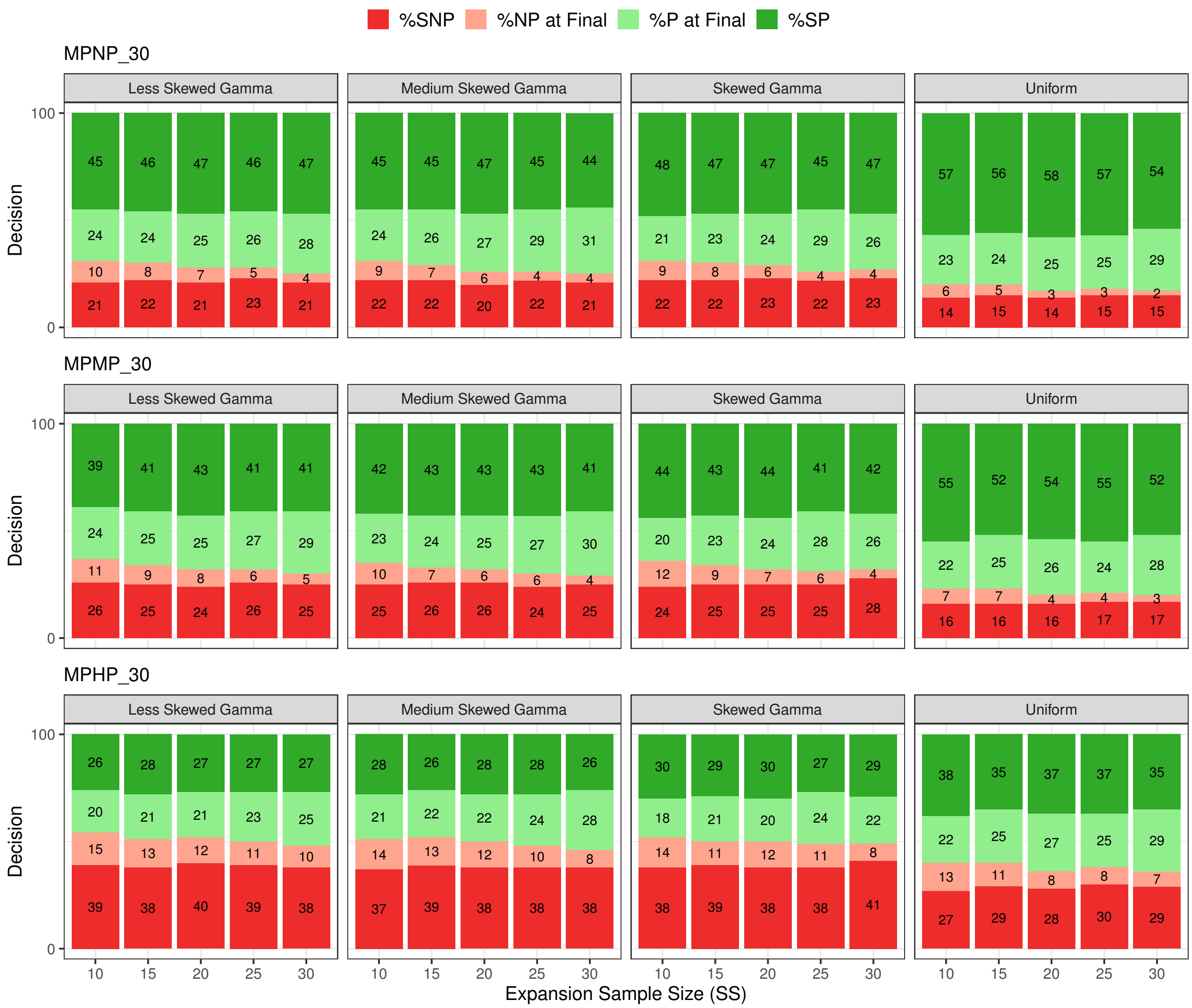

Figure 35: Percentage of simulations under the medium predictive scenarios with 30% predictive effect (5000 replications) that resulted in each decision category: predictive (%SP, dark green) or non-predictive (%SNP, dark red) at interim, or predictive (%P at Final, light green) or non-predictive (%NP at Final, light red) at final analysis. Results are by biomarker distribution ("Less Skewed", "Medium Skewed" and "Skewed" Gamma and Uniform) and expansion sample size for a 3-Bound configuration and 70% calibration rule.

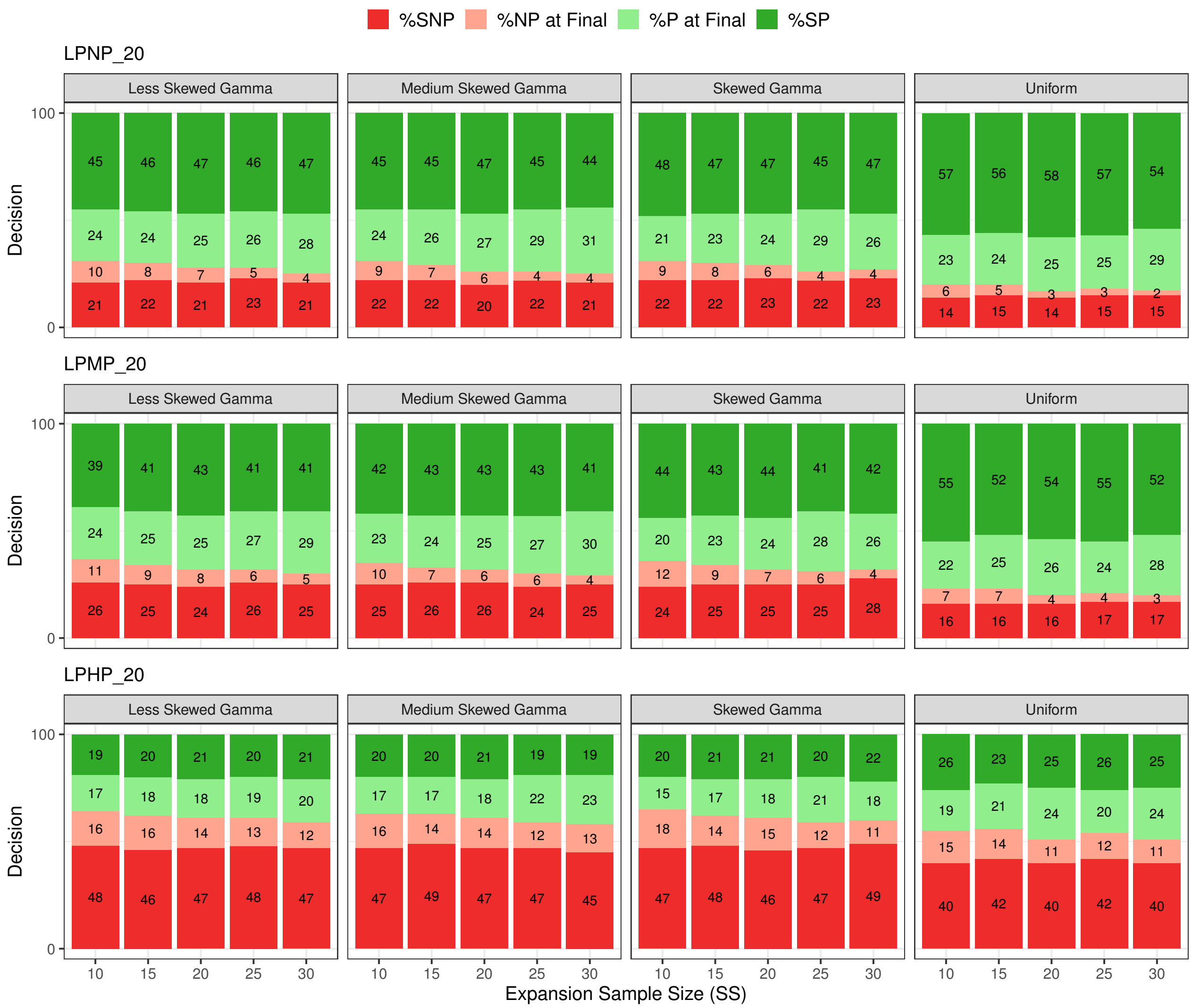

Figure 36: Percentage of simulations under the low predictive scenarios with 20% predictive effect (5000 replications) that resulted in each decision category: predictive (%SP, dark green) or non-predictive (%SNP, dark red) at interim, or predictive (%P at Final, light green) or non-predictive (%NP at Final, light red) at final analysis. Results are by biomarker distribution (“Less Skewed”, “Medium Skewed” and “Skewed” Gamma and Uniform) and expansion sample size for a 3-Bound configuration and 70% calibration rule.

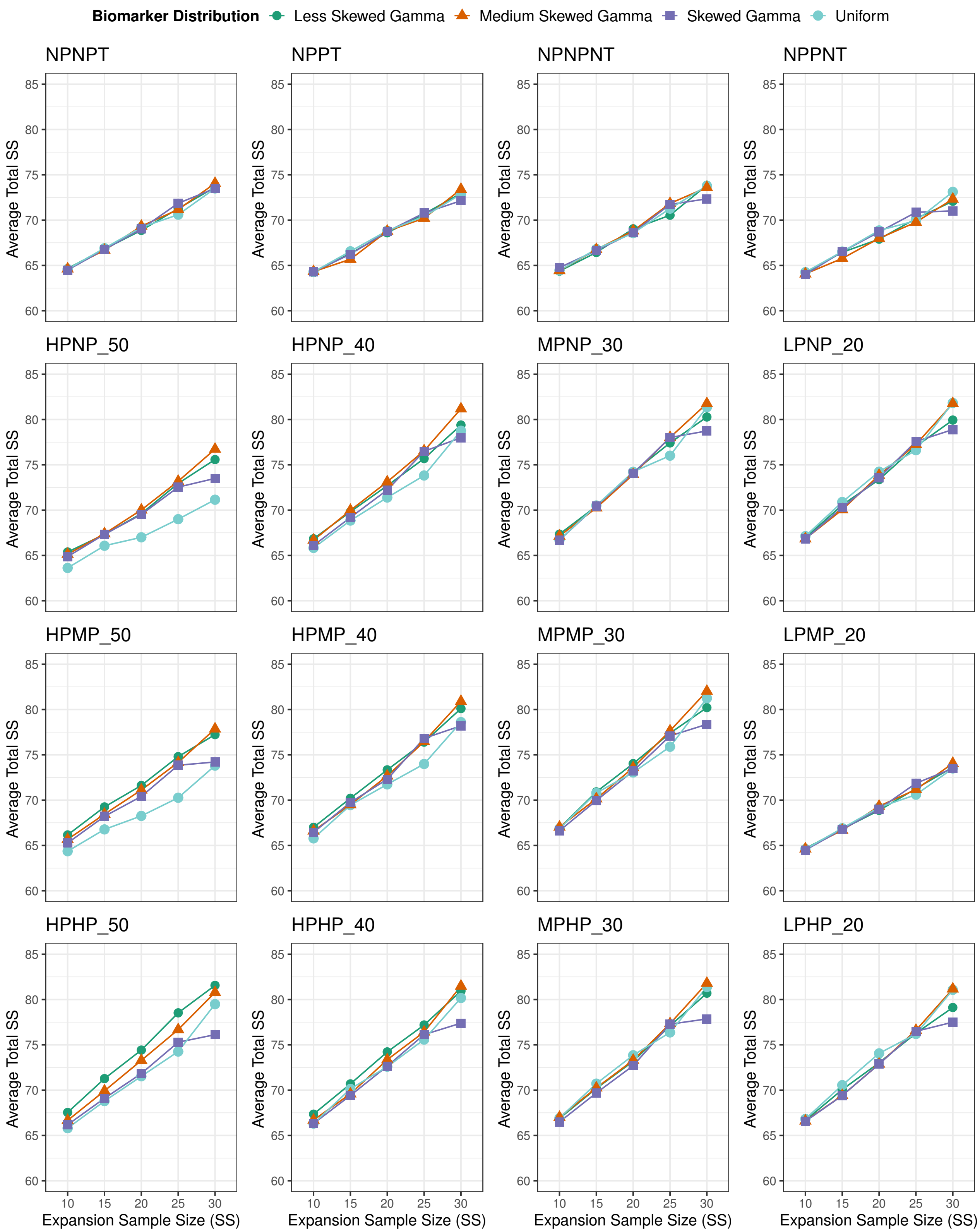

Figure 37: Average sample size under different biomarker distributions (5000 replications). Results are by expansion sample size for a 3-Bound configuration and 70% calibration rule.

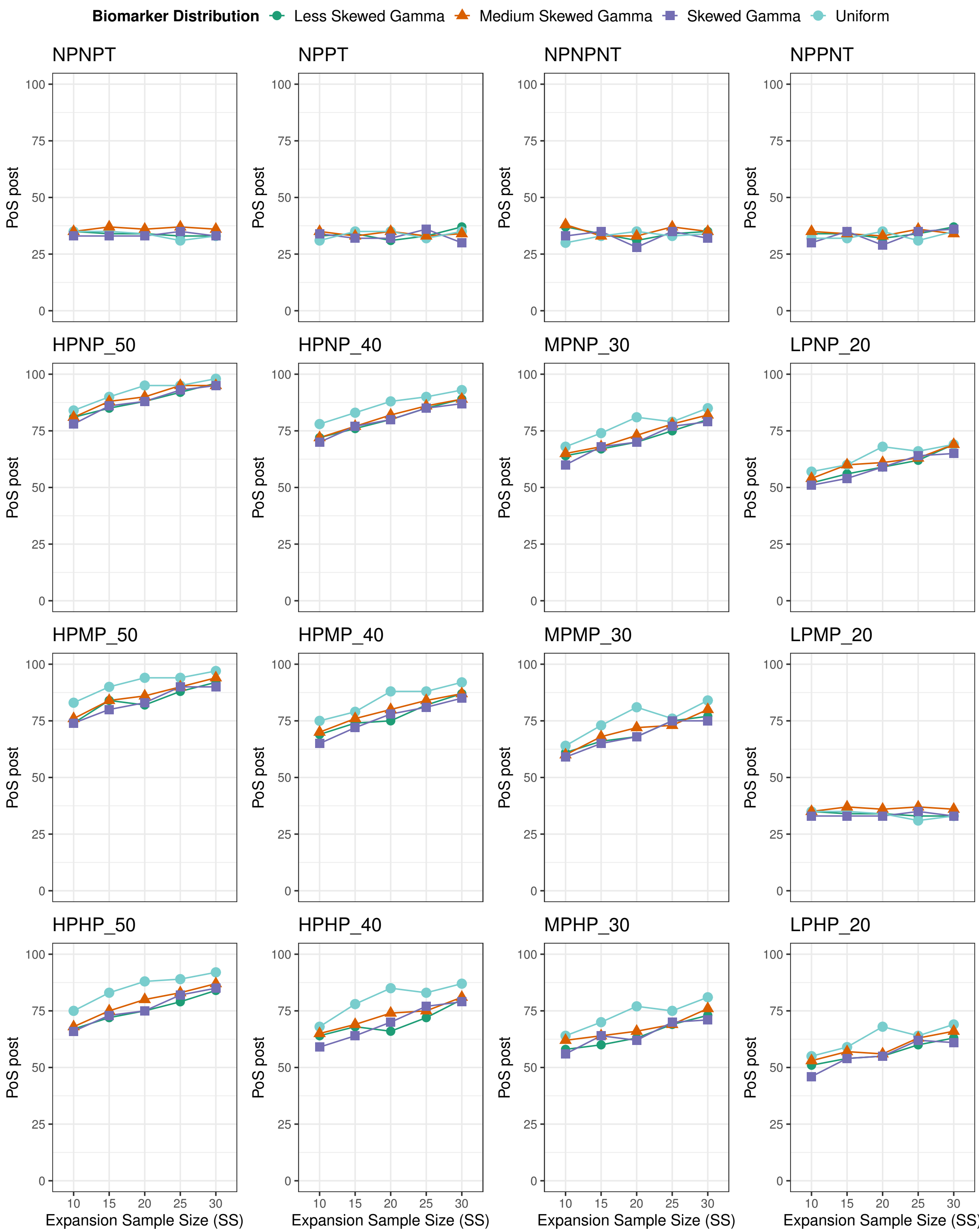

Figure 38: PoS post under different biomarker distributions (5000 replications). Results are by expansion sample size for a 3-Bound configuration and 70% calibration rule.

3.4.3 Interim Allocation Ratio

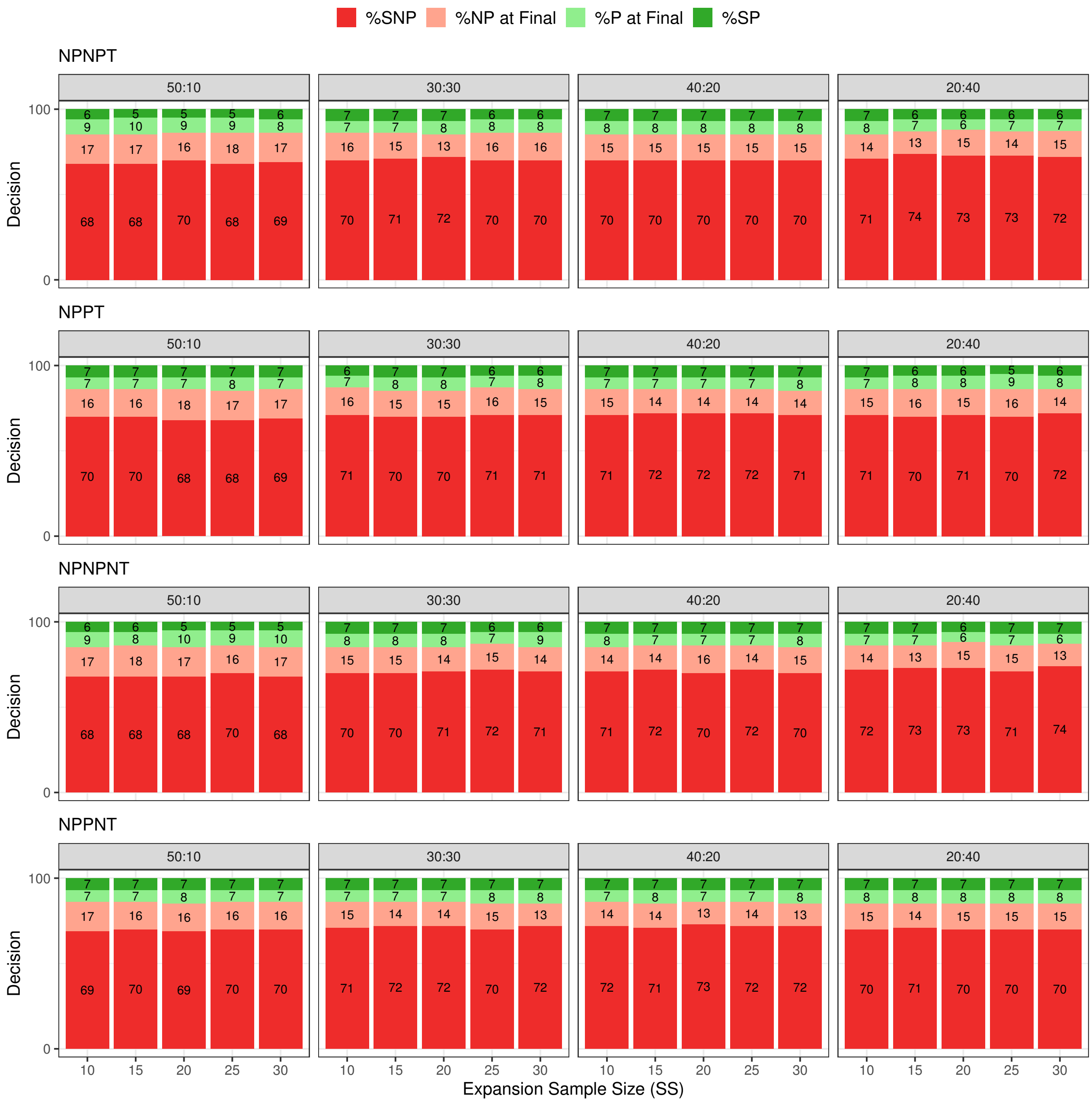

Figure 39: Percentage of simulations under the null scenarios (5000 replications) that resulted in each decision category: predictive (%SP, dark green) or non-predictive (%SNP, dark red) at interim, or predictive (%P at Final, light green) or non-predictive (%NP at Final, light red) at final analysis. Results are by interim allocation ratio (50:10, 40:20, 30:30 and 20:40) and expansion sample size for a 3-Bound configuration and 70% calibration rule.

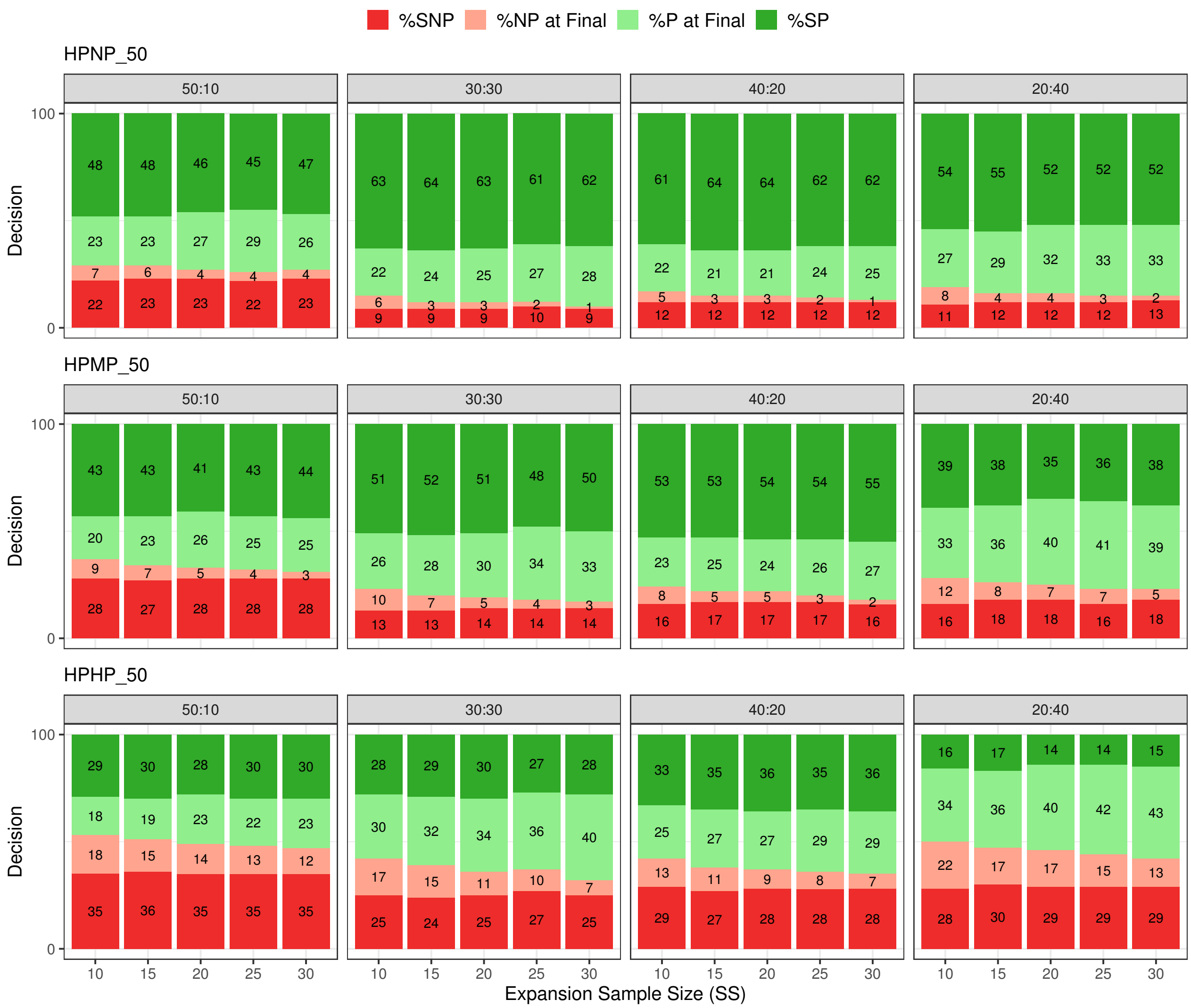

Figure 40: Percentage of simulations under the high predictive scenarios with 50% predictive effect (5000 replications) that resulted in each decision category: predictive (%SP, dark green) or non-predictive (%SNP, dark red) at interim, or predictive (%P at Final, light green) or non-predictive (%NP at Final, light red) at final analysis. Results are by interim allocation ratio (50:10, 40:20, 30:30 and 20:40) and expansion sample size for a 3-Bound configuration and 70% calibration rule.

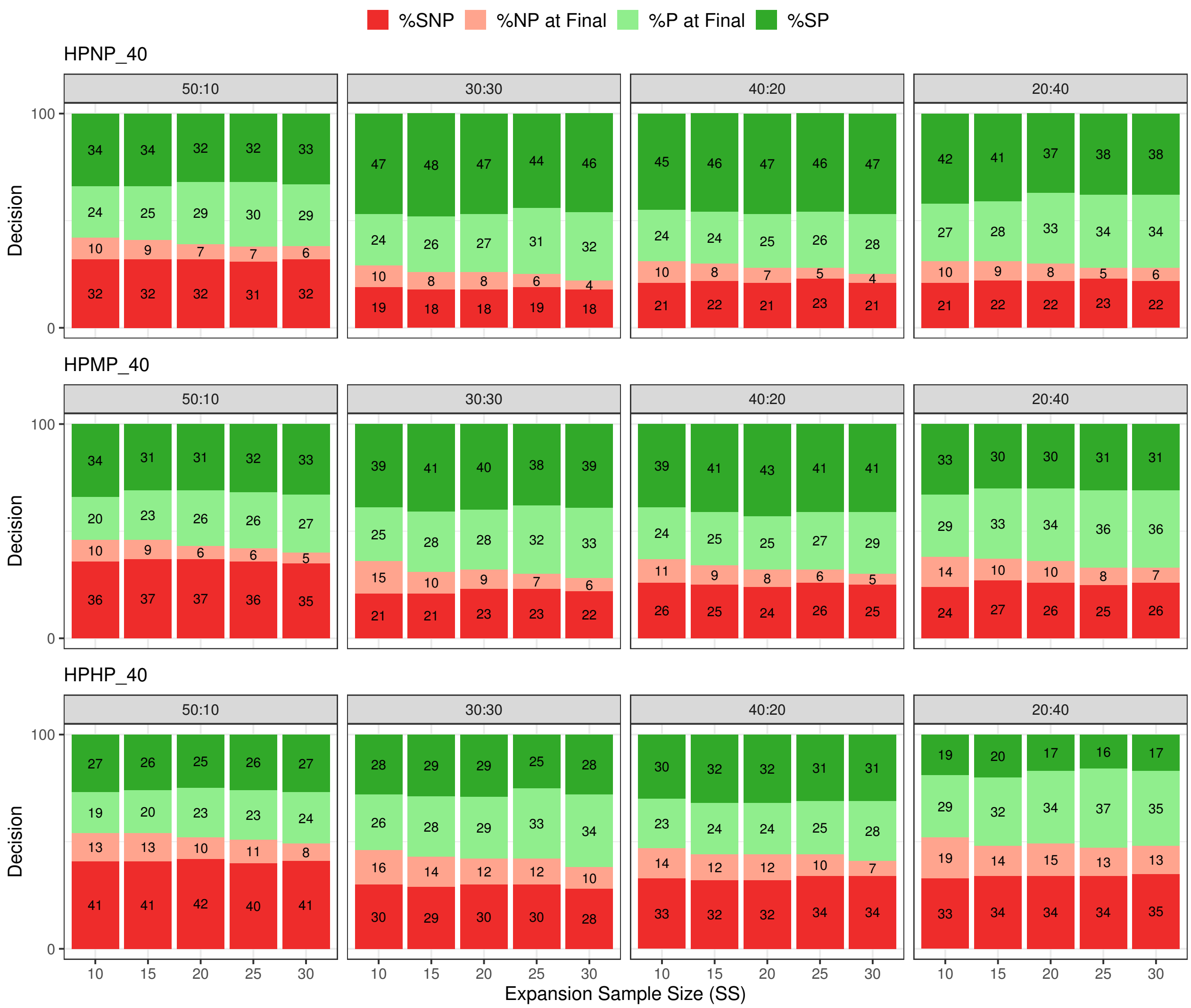

Figure 41: Percentage of simulations under the high predictive scenarios with 40% predictive effect (5000 replications) that resulted in each decision category: predictive (%SP, dark green) or non-predictive (%SNP, dark red) at interim, or predictive (%P at Final, light green) or non-predictive (%NP at Final, light red) at final analysis. Results are by interim allocation ratio (50:10, 40:20, 30:30 and 20:40) and expansion sample size for a 3-Bound configuration and 70% calibration rule.

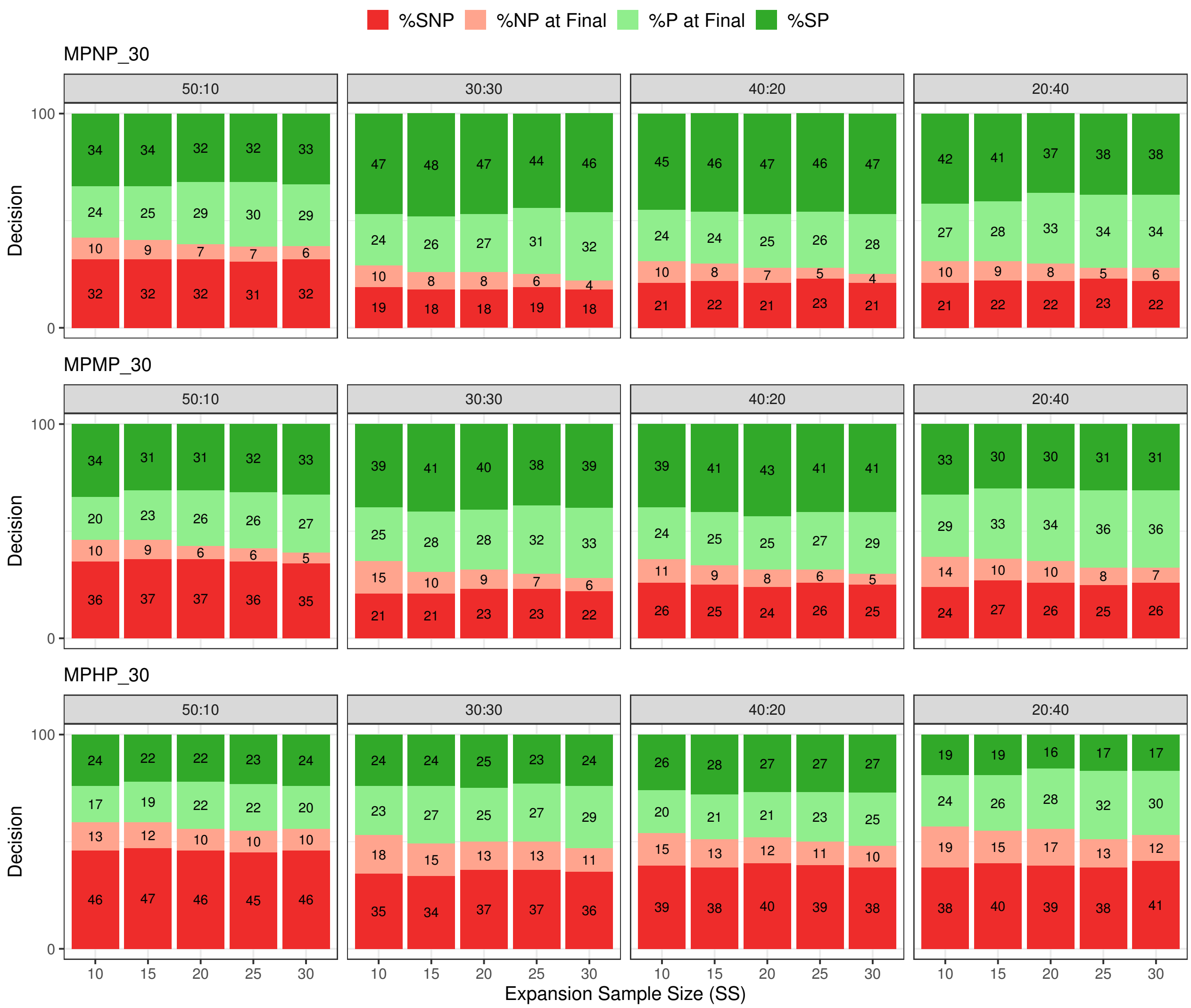

Figure 42: Percentage of simulations under the medium predictive scenarios with 30% predictive effect (5000 replications) that resulted in each decision category: predictive (%SP, dark green) or non-predictive (%SNP, dark red) at interim, or predictive (%P at Final, light green) or non-predictive (%NP at Final, light red) at final analysis. Results are by interim allocation ratio (50:10, 40:20, 30:30 and 20:40) and expansion sample size for a 3-Bound configuration and 70% calibration rule.

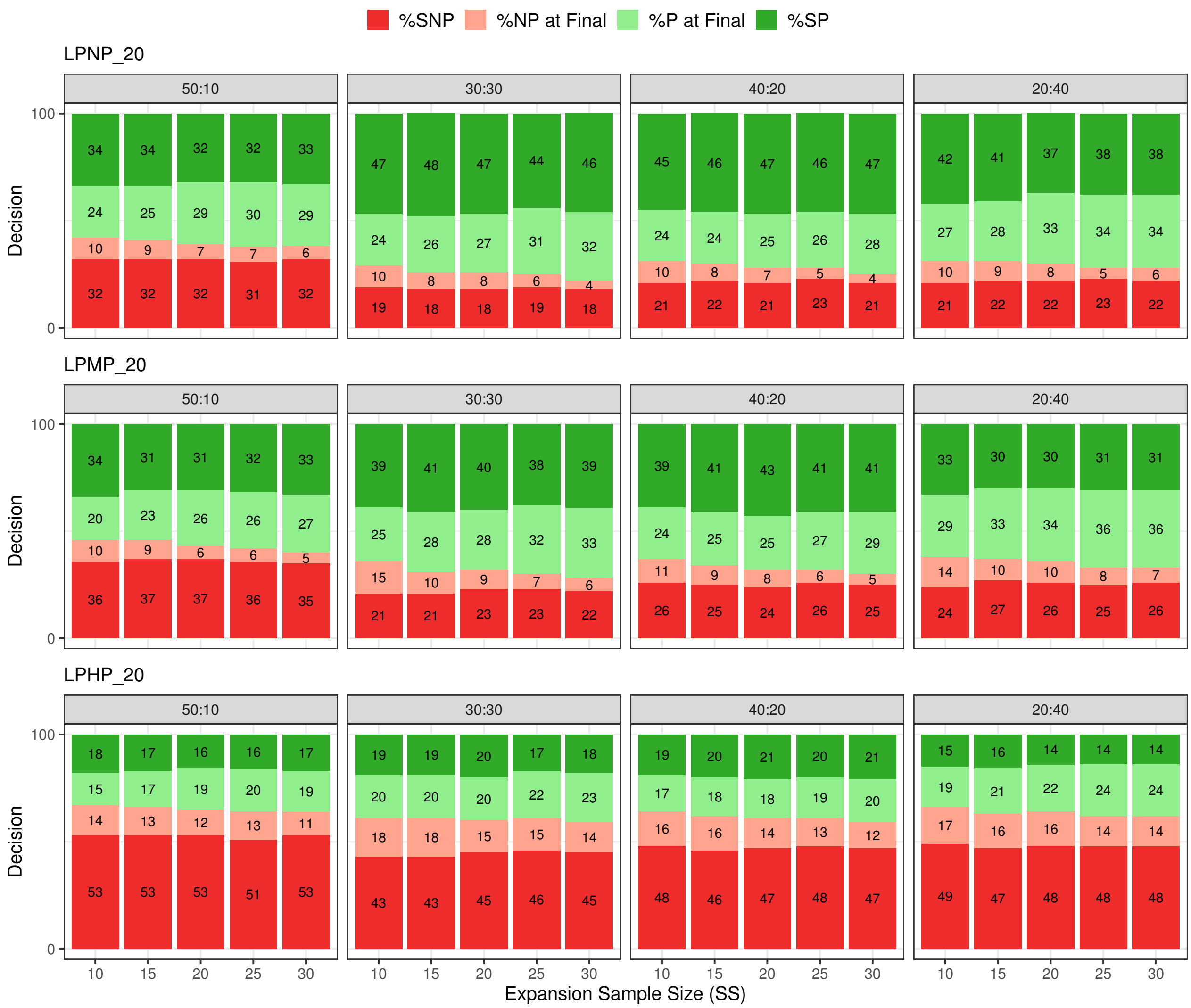

Figure 43: Percentage of simulations under the low predictive scenarios with 20% predictive effect (5000 replications) that resulted in each decision category: predictive (%SP, dark green) or non-predictive (%SNP, dark red) at interim, or predictive (%P at Final, light green) or non-predictive (%NP at Final, light red) at final analysis. Results are by interim allocation ratio (50:10, 40:20, 30:30 and 20:40) and expansion sample size for a 3-Bound configuration and 70% calibration rule.

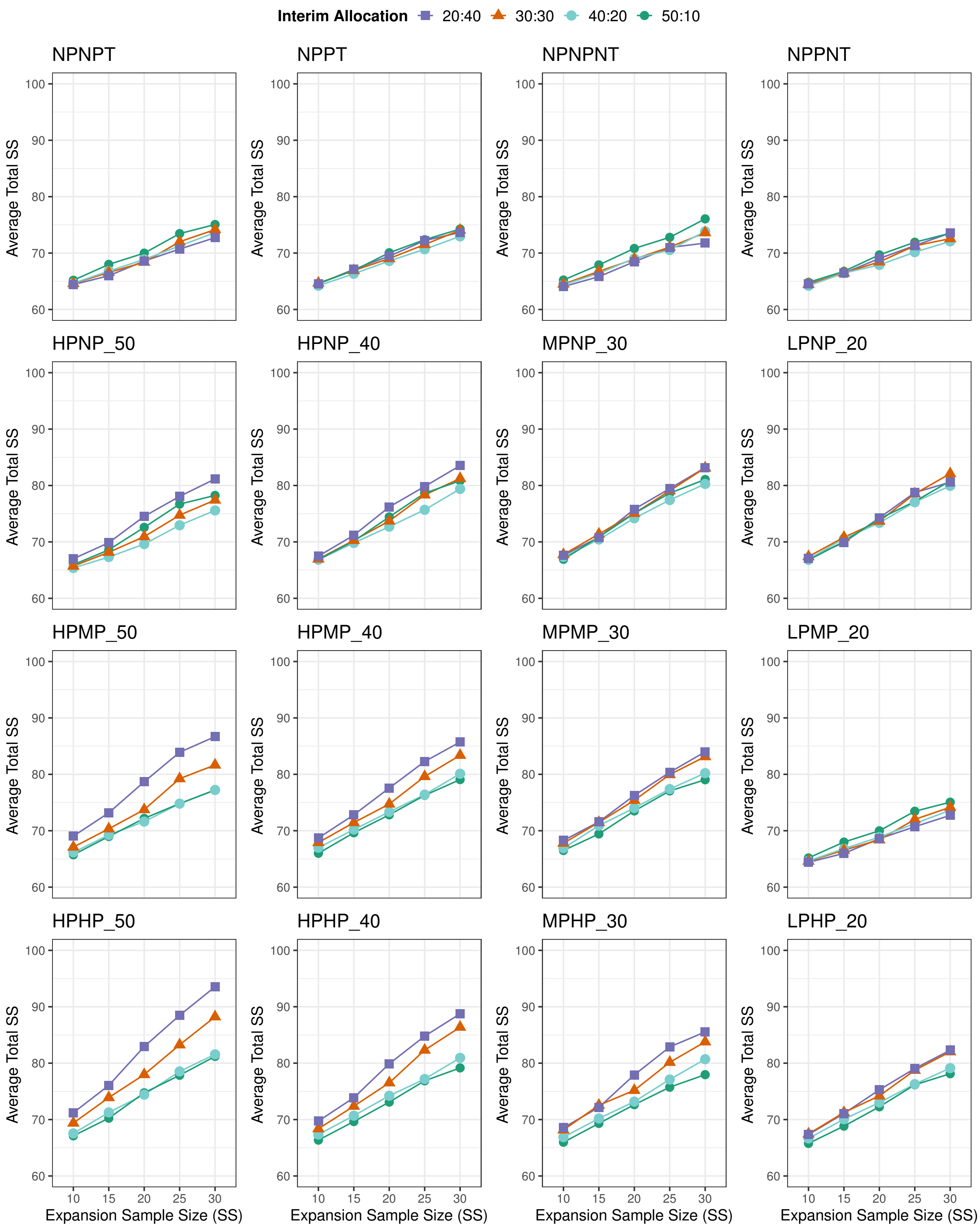

Figure 44: Average sample size under different interim allocation ratios (5000 replications). Results are by expansion sample size for a 3-Bound configuration and 70% calibration rule.

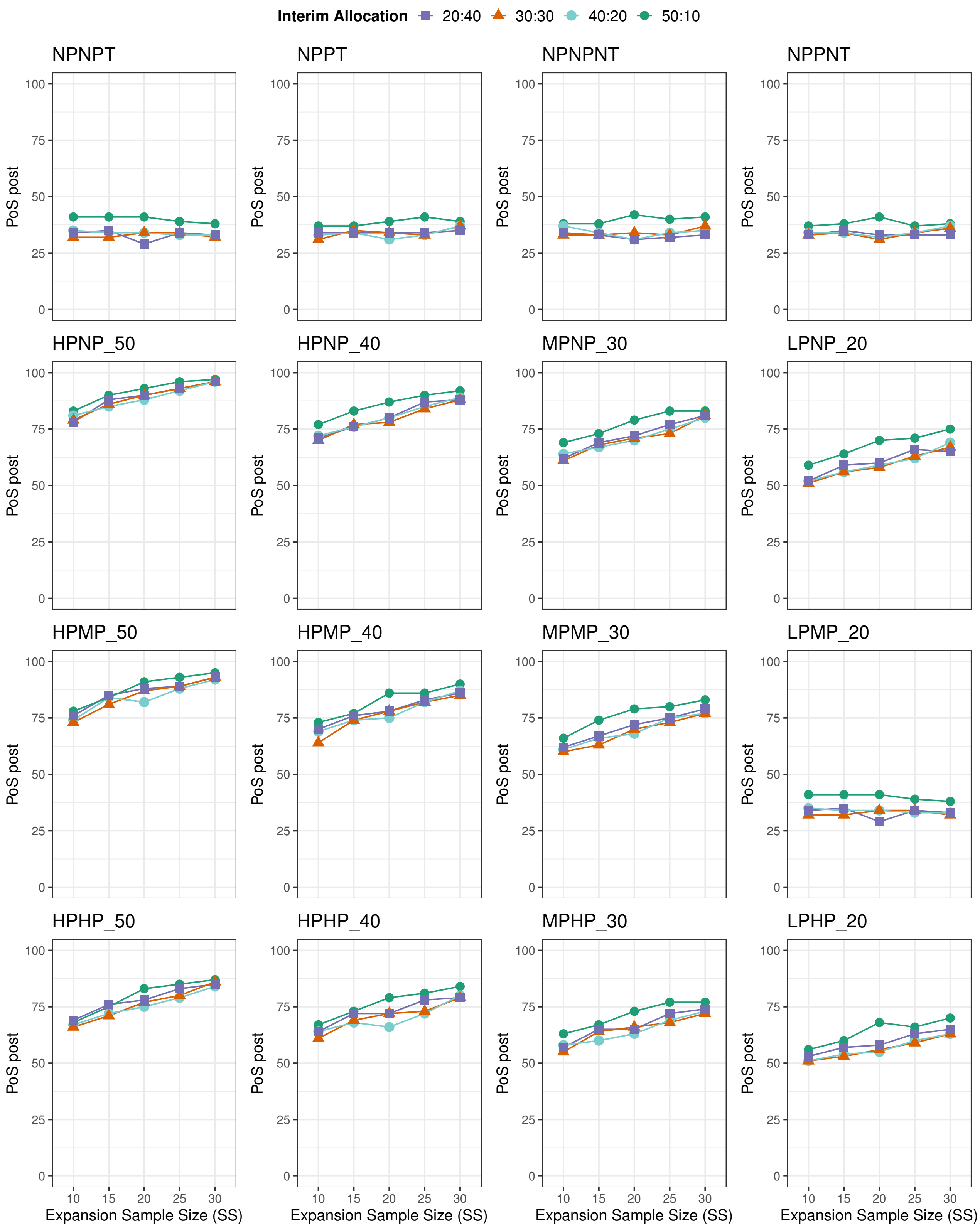

Figure 45: PoS post under different interim allocation ratios (5000 replications). Results are by expansion sample size for a 3-Bound configuration and 70% calibration rule.

3.4.4 Sensitivity of the Predictive Effect Estimate to the Presence of a Prognostic Effect

To assess whether the presence of a prognostic effect introduces multicollinearity into the model, thereby inflating standard errors and impacting the ability to detect predictive effects, two metrics were examined from the model output within the simulation studies:

- The standard error of the interaction term (the predictive effect);
- The correlation between the prognostic effect and the interaction coefficient.

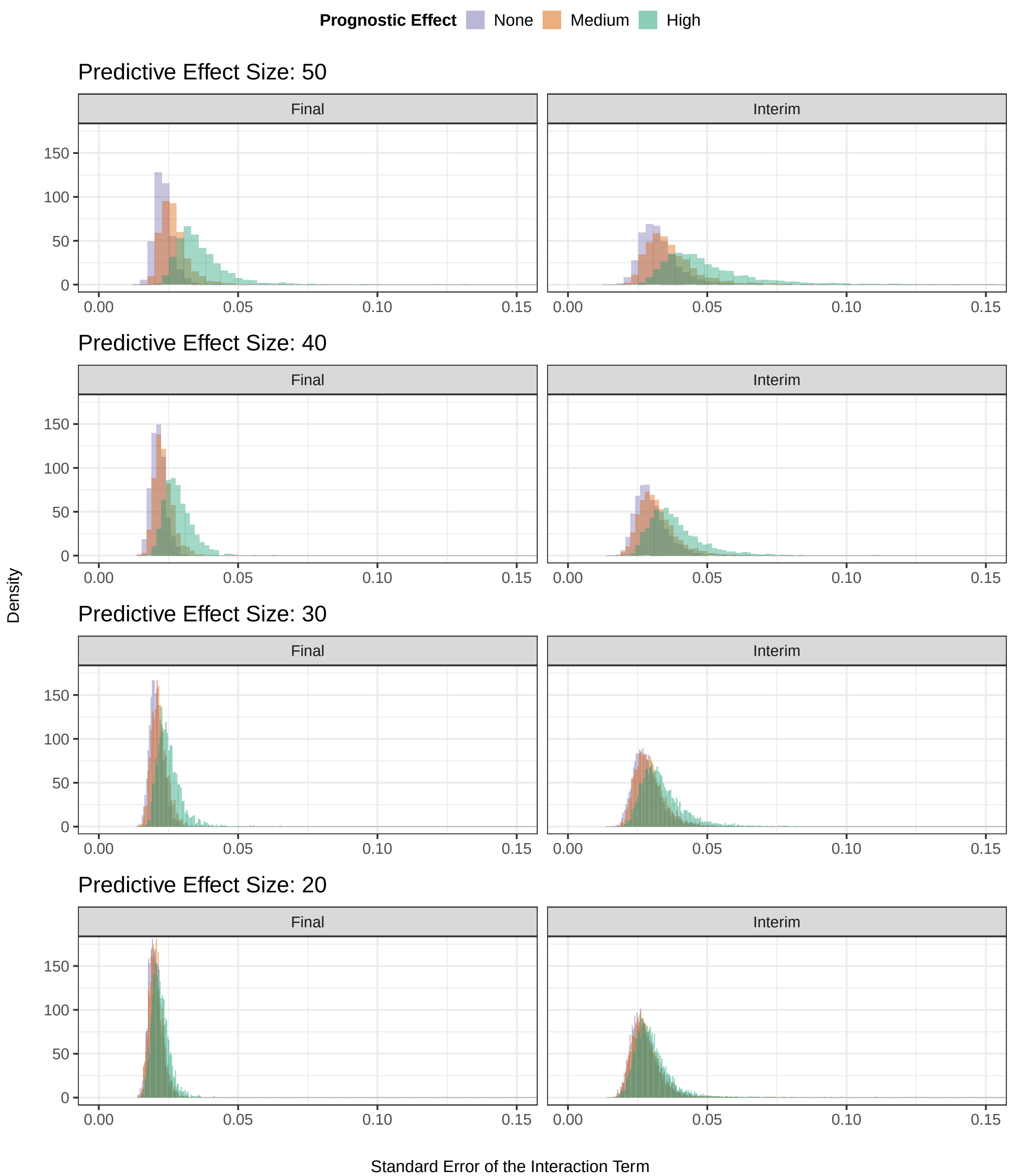

Figure 46: Standard error of the interaction term at both interim and final analysis.

These metrics were computed across the 16 scenarios for a design with 40 patients on the experimental arm, 20 on the control arm at interim, and 15 patients on each arm in the expansion phase. Results are provided in Figures 46 and 47.

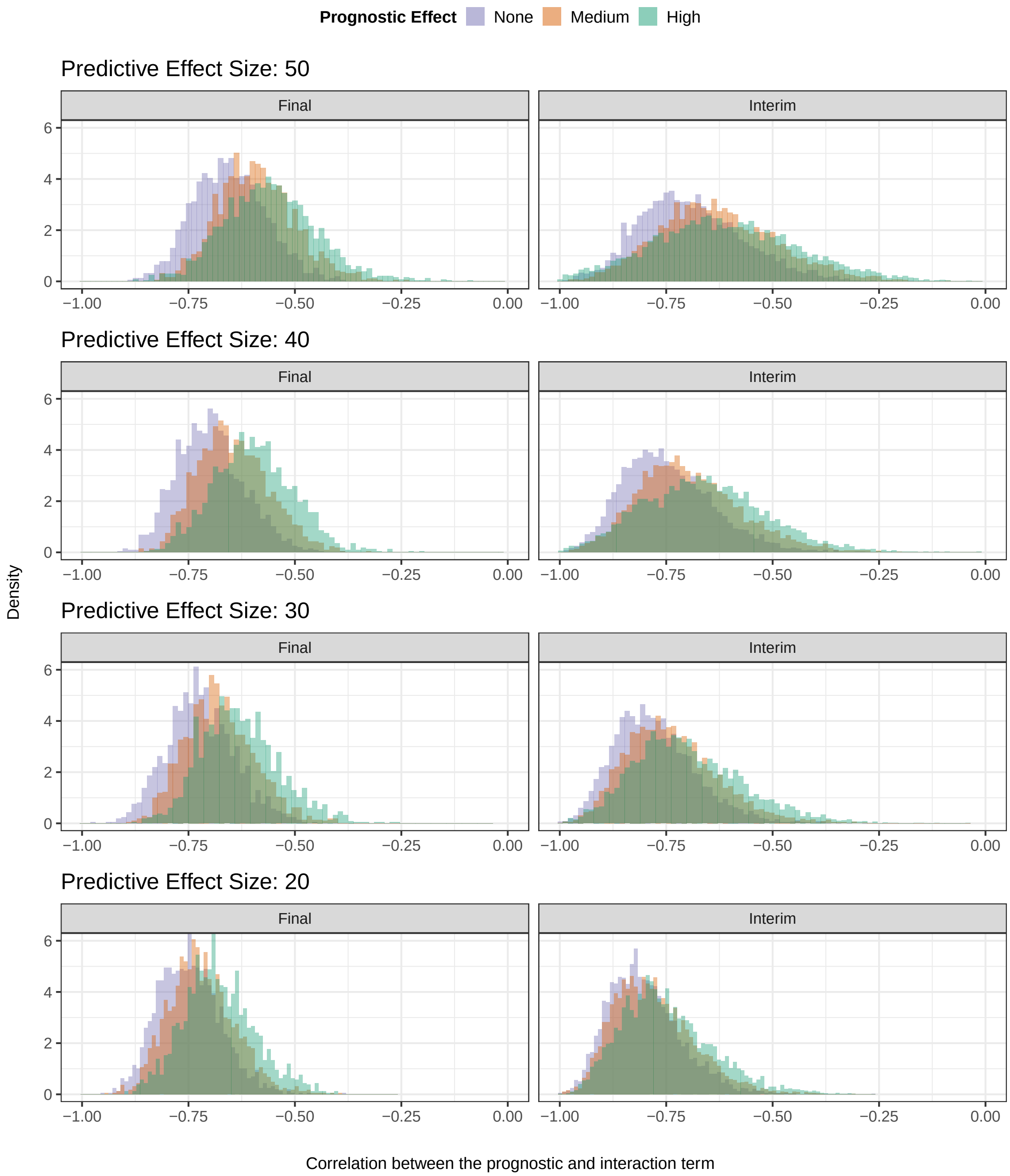

Figure 47: Correlations between the interaction and prognostic terms at both interim and final analysis.

The standard errors (SE) of the interaction term show a clear, consistent increase as the strength of the prognostic effect increases, both at interim and final analysis. Although this increase is modest (no more than 0.01 on average when comparing no prognostic effect to a high prognostic effect), given the small magnitude of the true interaction coefficients in these scenarios, which range from 0.02-0.05, this increase in SE is fairly

substantial, and contributes to why it is harder to detect a predictive effect in high-prognostic scenarios. The correlation between the prognostic and interaction coefficients, however, becomes slightly weaker (less negative) as the prognostic effect increases.

Together, these results suggest that stronger prognostic effects alter the relationship between the prognostic and interaction coefficients, as reflected by the trend in their estimated correlation, while also increasing the standard error of the interaction coefficient. Consequently, the interaction effect is estimated with reduced precision, making it more difficult to detect a predictive effect in scenarios with a strong prognostic effect and contributing to the observed reduction in power.

4 Illustrative Example

The impact of the interim analysis and the additional expansion cohort is illustrated through a single data example. Figure 48 plots the contrast curve (i.e. the difference between the predictive values under the control and experimental arm) for the analyses (where the sample sizes are 40 and 20 for the experimental and control arm respectively). In this data example, the interim data fails to result in a predictive/non-predictive conclusion, with an additional cohort recruited. Two post-interim allocation options are considered: equal allocation 20:20 to both the experimental and control arms, and unequal allocation 10:30 in favour of the control arm to ensure balance over the total sample sizes. For both options, the final sample size is $N = 100$. To generate this data example, the interim data is first generated. For both final analysis options, the post-interim data was generated separately and combined with the interim data.

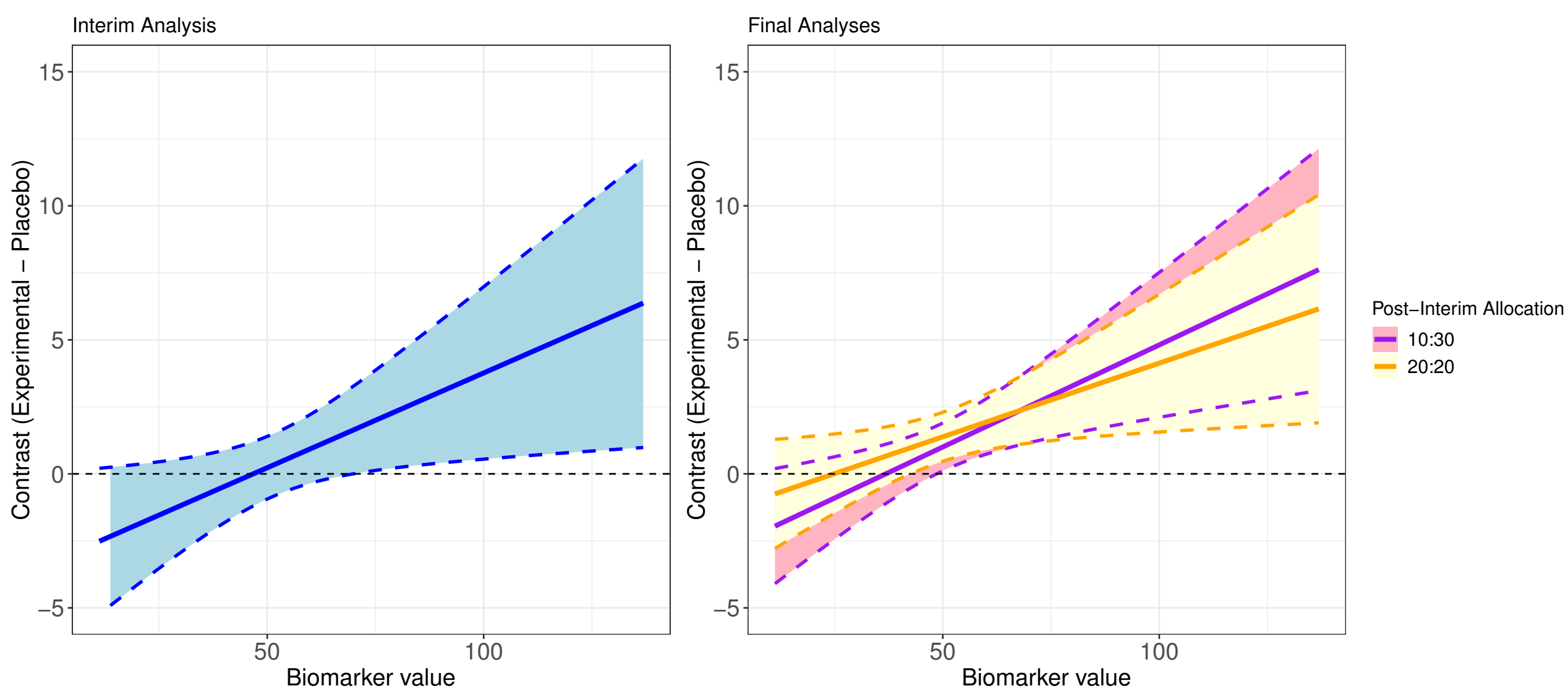

Figure 48: Example contrast curve (difference between predictive values under the control and experimental arms) for a single data-set, alongside the 95% confidence intervals. The curves are provided for the interim analysis and for two versions of the final analysis: equal allocation of 20:20 to the experimental and control arm and unequal allocation of 10:30 such that both arms have equal final sample size of $n_1 = n_2 = 50$.

It was previously demonstrated that there was no difference in power between unequal and equal post-interim allocation. The cause of this can be deduced from 48. At the final analysis stage, after interim, the contrast curves change based on the post-interim data as the additional information is used to update the regression coefficients when implementing AKSA. AKSA itself relies on comparing the shapes of the response curves (taking the contrast between the experimental and control arms) to declare predictiveness. As a result,

even though the post-interim data may slightly update the response curves and confidence intervals, the shape is fairly stable post-interim given the accrued data at the interim stage. This means that the power of detecting a predictive effect remains largely unchanged for different post-interim allocation ratios given that the total sample size N is constant.

Most importantly, AKSA detects a predictive effect using the probability that the difference between response curves ($P(D_X > 0)$) exceeds a pre-specified threshold (η_2) averaged across the biomarker range. This can lead to very similar power even if the contrast probabilities, $P(D_X > 0)$, slightly change. For the data example, the AKSA probabilities $P(D_X > 0)$, remain very similar at 0.89 under the unequal allocation and 0.86 under the equal allocation ratio. Given that the probabilities aren't directly used themselves, with predictiveness declared based on a comparison to a threshold η_2, despite differences, both lead to the same conclusion: declaring a predictive effect of the biomarker.

5 Multiple Interim Analyses

The previously presented simulation results considered a design with a single interim analysis used to determine whether an expansion phase is required for declaring that a biomarker has a predictive effect. While this was a design choice based on the motivating example, the framework is akin to a group sequential design (GSD), and therefore can accommodate any number of interim analyses.

Given the small sample size setting, with just 40 patients on the experimental arm and 20 patients on the control arm as the initial sample size, including an additional interim earlier in the study (i.e. before the initial 40:20 sample sizes are recruited) may lead to unstable estimates of the predictive effect due to the limited amount of data available. In contrast, including an additional interim in the expansion phase may pose advantages. This additional interim could be used to make an earlier assessment of the predictive effect of the biomarker compared to a single interim design that proceeds from expansion straight to final analysis, thereby saving resources. However, including additional interim analyses also increases operational complexity. Each interim requires data collection and analysis, as well as decision-making before recruitment can proceed. This could increase trial durations, administrative burden and financial costs. As inference focuses on the estimation of a biomarker effect, the cost of additional measures of the biomarker must also be considered.

To explore the impact of including an additional interim analysis within the expansion phase, an additional simulation study was conducted. In this simulation study, we assume a single additional interim analysis conducted after the initial expansion interim and before the final analysis. To simplify the setting, only equal allocation in the expansion phase was considered, with a single calibration rule of 70% at the first interim. However, all expansion sample sizes (10, 15, 20, 25, and 30) were considered. The second interim analysis was conducted when half of the expansion sample size had been recruited and full observed in each arm.

To implement such a design, additional decision parameters and calibration rules must be considered. Assume that the first interim data is denoted as Ω_0; the second interim data is denoted Ω_1, which includes interim from before and after the first interim; and the final data-set includes all patients throughout the trial (both before and after each interim) and is denoted as Ω_1. The decision path-way is structured as follows:

- If $P(D_X > 0|\Omega_0) < \eta_0$ at interim, the biomarker is said to not show a sufficient predictive effect based on the interim data. Given the clear lack of a predictive signal, no expansion is required and no further patients are recruited. The biomarker is declared as non-predictive.

- If $P(D_X > 0|\Omega_0) > \eta_1$ at interim, the biomarker is declared to have a predictive effect based on the interim data. Given that the interim sample sizes ($n_{0,1},\ n_{1,1}$) are sufficient for demonstrating a predictive effect, no further patients are required, thus no expansion occurs and the trial terminates.

- If $\eta_0 \leq P(D_X > 0|\Omega_0) \leq \eta_1$ at interim, there is insufficient evidence to declare the biomarker as predictive or non-predictive at this stage. Thus, a larger sample size is required to provide sufficient evidence, so the trial enters an expansion phase with a further $n_{0,2}/2$ and $n_{1,2}/2$ patients recruited:
  - If $P(D_X > 0|\Omega_1) < \eta_2$ at the second interim, the biomarker is said to not show a sufficient predictive effect. Recruitment is stopped and the biomarker declared as non-predictive.
  - If $P(D_X > 0|\Omega_1) > \eta_3$ at the second interim, the biomarker is declared to have a predictive effect. No further patients are required, thus the trial terminates.
  - If $\eta_2 \leq P(D_X > 0|\Omega_1) \leq \eta_3$ at the second interim, there is insufficient evidence to declare the biomarker as predictive or non-predictive. Further information is required, thus a further $n_{0,2}/2$ and $n_{1,2}/2$ patients are recruited and the trial proceeds to final analysis:
    - If $P(D_X > 0|\Omega_2) > \eta_4$, the biomarker is declared as predictive at final analysis, otherwise it is declared as non-predictive.

The trial therefore consists of five decision bounds, $\eta = \{\eta_0, \eta_1, \eta_2, \eta_3, \eta_4\}$, all of which must be carefully calibrated to control the type I error rate without compromising power. The calibration is conducted under all four null-scenarios with no predictive effect, and is specified as follows:

- η_0 is calibrated such that in at least 70% of trials, the biomarker is deemed non-predictive at the first interim under the four null scenarios.
- η_1 is calibrated such that approximately 7.5% of trials declare the biomarker as predictive at first interim across the four null scenarios. This allocates half of the overall 15% type I error is spent at the first interim.
- η_2 is calibrated such that in at least 50% of trials that proceed to expansion declare the biomarker as non-predictive at the second interim across the four null scenarios.
- η_3 is calibrated such that approximately 3.75% of trials declare the biomarker as predictive at second interim across the four null scenarios. This allocates a quarter of the overall 15% type I error is spent at the second interim.
- η_4 is calibrated such that the overall type I error rate of the trial remains at or below 15% under the four null scenarios.

The η_0, η_2 and η_4 calibration specifications align with the single interim design to allow for direct comparisons between the operating characteristics of the single and multiple interim designs. The calibrated parameters are displayed in Table 14. Note, different calibration specifications can and should be explored to investigate the optimal operating characteristics. However, to simplify the simulation study, only a single configuration is considered.

Additional metrics are considered in the multiple interim design:

- % SNP (1) and % SP (1) are the probabilities of declaring a non-predictive or predictive effect at the first interim, terminating recruitment.
- % SNP (2) and % SP (2) are the probabilities of declaring a non-predictive or predictive effect at the second interim, terminating recruitment.
- PoS (1) is the probability that the biomarker is ultimately deemed predictive given that the trial enters the expansion phase after the first interim.

Table 14: Calibrated decision bounds η_0, η_1, η_2, η_3 and η_4 under the multiple interim framework.

Decision	Post-Interim SS				
Bound	10	15	20	25	30
η_0	0.640	0.640	0.640	0.640	0.632
η_1	0.792	0.787	0.789	0.794	0.787
η_2	0.719	0.708	0.696	0.702	0.682
η_3	0.786	0.785	0.782	0.795	0.784
η_4	0.758	0.709	0.697	0.682	0.716

- PoS (2) is the probability that the biomarker is deemed predictive given that the trial enters the expansion phase and continues past the second interim to the final analysis.

The simulation is conducted under the previously considered null and high-predictive scenarios (5000 simulation replications), with results for both the single interim and multiple interim designs provided in Tables 15 and 16. Under all four null scenarios and sample sizes, the single and multiple interim designs are comparable in terms of T1E and % SP and % SNP at first interim. This is the result of fixed calibration across the two designs. Adding an additional interim analysis does uniformly reduce the average sample size by 1-6 patients, as fewer trials continue to the final analysis. In fact, 11-18% of trials under these null scenarios are terminated at the second interim stage. This reflects the benefits of the additional interim in terms of earlier termination and resource management. The probability of a predictive deceleration given expansion after the first interim (PoS (1)) is uniformly higher than than the probability of a predictive deceleration given recruitment continues past the second interim (PoS (1)). PoS (1) under the multiple interim analysis design is fairly comparable to the PoS under the single interim. This suggests that the probability of declaring a predictive effect in the expansion phase is similar regardless of the additional interim analysis.

In terms of the non-null scenarios, the additional interim analysis reduces power by up 5%, however, under several scenarios power is consistent between the two designs. The most notable differences in power are under the 10:10 expansion sample sizes, where the second interim is conducted after further 5 patients are recruited in each arm. This provides little additional evidence of a predictive effect compared to the first interim, and thus produces less accurate estimates, with more trials declaring the biomarker as non-predictive at the second interim (% SNP) compared to larger sample sizes. Generally, power would be expected to be lower due to the additional interim and the additional opportunity to wrongly declaring the biomarker as non-predictive. However, this is mitigated by the calibration of the decision bounds, which are generally less conservative for the final analysis compared to the single interim design, allowing more biomarkers to be declared as predictive.

The multiple interim design offers consistent reductions in the average sample size by up to 7 patients. This is driven by the additional early stopping opportunities at the second interim. This gain in sample size reduction is most notable for the larger expansion sample sizes. Comparing PoS under the single interim design and PoS (1) under the multiple interim design, given that the expansion sample size is greater than 10 patients per arm, both produce reasonably similar probabilities of declaring the biomarker as predictive given the trial proceeds to expansion. Under the 10:10 expansion sample size, the additional interim decreases PoS in line with the power.

Overall, the single interim design offers a marginal, but largely negligible, improvement in power over the multiple interim design. However, the introduction of a multiple interim proves advantageous in terms of average sample size. Therefore, considering the trade-off in power and trial efficiency, the multiple interim design is favourable.

While the proposed framework can naturally accommodate multiple interim analyses, careful consideration is required to balance the potential gains in efficiency against the increased operational burden. Additionally, the more interim analyses that are conducted increases the statistical burden, with two additional decision parameters required for each interim. As each decision parameters must be calibrated to achieve a desired calibration rule, computational intensity increases.

Table 15: Simulation results under the null scenarios comparing the single interim design (under the 3-bound design with 70% calibration rule) to the multiple interim design, i.e. the design with an additional interim in the expansion phase.

		Single Interim					Multiple Interim							
**Scenario**	**Post-Interim SS**	**T1E**	**% SNP**	**% SP**	**Av N**	**PoS**	**T1E**	**% SNP (1)**	**% SP (1)**	**% SNP (2)**	**% SP (2)**	**Av N**	**Pos (1)**	**Pos (2)**
NPNPT	10	15	70	7	65	35	12	69	6	14	3	63	23	37
	15	15	70	7	67	34	15	69	8	13	4	64	31	53
	20	15	70	7	69	34	14	72	8	11	3	66	33	55
	25	15	70	7	71	33	14	71	6	13	4	68	33	56
	30	15	70	7	74	33	14	71	7	13	3	68	29	55
NPPT	10	14	71	7	64	33	14	70	7	12	3	63	28	37
	15	14	72	7	66	34	14	74	9	9	2	64	31	56
	20	14	72	7	69	31	15	73	7	10	4	65	39	64
	25	14	72	7	71	33	14	72	7	12	2	67	35	74
	30	15	71	7	73	37	15	71	8	11	4	69	33	49
NPNPNT	10	15	71	7	64	37	15	68	7	14	4	63	31	54
	15	14	72	7	66	34	14	72	6	12	3	64	34	63
	20	14	70	7	69	31	14	72	7	12	3	66	35	64
	25	14	72	7	71	34	15	71	7	12	3	67	36	66
	30	15	70	7	74	35	13	70	7	13	2	69	26	46
NPPNT	10	14	72	7	64	34	13	70	8	13	3	63	22	34
	15	15	71	7	66	34	14	72	6	11	3	64	35	60
	20	14	73	7	68	32	15	71	7	12	4	66	34	55
	25	14	72	7	70	34	15	74	7	10	3	67	41	74
	30	15	72	7	72	37	14	72	7	11	3	69	31	46

Table 16: Simulation results under the high predictive scenarios comparing the single interim design (under the 3-bound design with 70% calibration rule) to the multiple interim design, i.e. the design with an additional interim in the expansion phase.

		Single Interim					Multiple Interim							
**Scenario**	**Post-Interim SS**	**Power**	**% SNP**	**% SP**	**Av N**	**PoS**	**Power**	**% SNP (1)**	**% SP (1)**	**% SNP (2)**	**% SP (2)**	**Av N**	**Pos (1)**	**Pos (2)**
HPNP_50	10	83	12	61	65	81	80	11	61	6	12	64	68	71
	15	85	12	64	67	85	83	11	64	5	11	65	74	82
	20	85	12	64	70	88	84	13	61	2	16	67	89	92
	25	86	12	62	73	92	85	12	62	3	16	68	87	96
	30	87	12	62	76	96	88	9	65	2	19	69	90	87
HPMP_50	10	76	16	53	66	74	71	16	53	9	11	64	57	62
	15	78	17	53	69	84	78	16	56	5	13	66	79	89
	20	78	17	54	72	82	78	18	56	4	14	67	84	92
	25	80	17	54	75	88	78	17	59	5	17	72	84	95
	30	82	16	55	77	92	81	15	55	3	18	72	87	87
HPHP_50	10	58	29	33	68	67	52	31	35	12	11	65	52	61
	15	62	27	35	71	72	60	29	35	9	12	67	69	85
	20	63	28	36	74	75	62	29	34	7	15	70	77	89
	25	64	28	35	79	79	62	29	32	8	15	74	78	95
	30	65	28	36	82	84	62	28	36	8	16	75	71	79
HPNP_40	10	69	21	45	67	72	64	22	47	11	8	64	55	73
	15	70	22	46	70	76	68	22	46	8	13	66	70	84
	20	72	21	47	73	80	72	22	49	5	14	68	79	91
	25	72	23	46	76	85	70	22	44	7	16	72	77	90
	30	75	21	47	79	89	73	20	48	4	17	73	79	79
HPMP_40	10	63	26	39	67	69	61	24	42	9	11	65	57	58
	15	66	25	41	70	74	65	25	44	7	12	66	69	80
	20	68	24	43	73	75	68	23	43	6	14	70	75	86
	25	68	26	41	76	82	68	23	39	8	18	73	77	92
	30	70	25	41	80	87	69	23	43	6	16	74	77	84
HPHP_40	10	53	33	30	67	64	49	31	32	14	9	65	46	58
	15	56	32	32	71	68	55	31	33	12	10	67	62	86
	20	56	32	32	74	66	56	33	34	9	12	69	66	81
	25	56	34	31	77	72	57	33	31	9	14	73	72	89
	30	59	34	31	81	80	60	30	34	7	15	75	73	77
